# Septin7 is essential in early hematopoiesis, but redundant at later stages

**DOI:** 10.26508/lsa.202603637

**Published:** 2026-07-13

**Authors:** Natalia Ronkina, Nikita A Verheyden, Prerna Gambhir, Kathrin Laass, Tatiana Yakovleva, Anneke Dörrie, Megha Abbey, Manoj B Menon, Anton Selich, Melanie Galla, Michael Rothe, Axel Schambach, Stephanie Buchheister, Andreas Krueger, Alexey Kotlyarov, Matthias Gaestel

**Affiliations:** 1 Institute of Cell Biochemistry, Hannover Medical School , Hannover, Germany; 2 Molecular Immunology, Justus-Liebig-University Giessen , Giessen, Germany; 3 CuraTeQ Biologics, Telangana, India; 4 Kusuma School of Biological Sciences, Indian Institute of Technology Delhi , New Delhi, India; 5 Institute of Experimental Hematology, Hannover Medical School , Hannover, Germany; 6 Institute for Laboratory Animal Science and Central Animal Facility, Hannover Medical School , Hannover, Germany

## Abstract

The unique cytoskeletal protein Septin7 is generally considered to be required for cytokinesis in yeast and mammals. Whole-body genetic ablation of *Septin7* in mice is embryonically lethal. *Septin7-*deficient fibroblasts and HeLa cells are defective in cytokinesis and undergo obligate multinucleation. Surprisingly, lymphocyte- and myeloid-specific deletion of Septin7 in mice caused no detectable defects in blood lineage development, indicating dispensability of Septin7 for hematopoiesis. However, *Septin7*-deficient hematopoietic stem cells failed to engraft and establish donor chimerism after transplantation. To reconcile these contradictory findings, we examined the consequences of Septin7 deletion at distinct stages of hematopoiesis. Our results demonstrate that Cre-induced deletion of the floxed *Septin7* allele is inefficient during the early stages of hematopoiesis, suggesting strong selection pressure against Septin7 deficiency at this stage. In contrast, deletion of *Septin7* at the common lymphoid progenitor stage, as well as in *Hoxb8*-immortalized hematopoietic progenitors, is efficient and does not result in any noticeable defects in cell division. Taken together, our findings indicate that Septin7 is essential during early hematopoiesis but becomes dispensable at later stages.

## Abstract (precis)

Septin7 is essential for hematopoietic stem cell development but dispensable for cytokinesis in *Hoxb8*-immortalized myeloid and lymphoid progenitors. Moreover, it is not required for lymphoid development under either steady-state conditions or physiological stress induced by oncogenic *K-Ras*-driven tumorigenesis.

## Introduction

Cytokinesis in yeast and animals depends on the contraction of the cell membrane at an equatorial furrow. This contraction is powered by a ring-like structure composed of actin, myosin, and several cytoskeleton-associated proteins. Effective cytokinesis is achieved by a comprehensive coordination of cell-cycle control, spatial organization, rearrangement of the cytoskeleton, generation of mechanical forces, and the trafficking of cellular membranes (Hartwell et al, 1970; Fededa & Gerlich, 2012; Green et al, 2012; Addi et al, 2018; Karasmanis et al, 2019; Marquardt et al, 2021). Initially, identified as essential genes for cytokinesis in budding yeast, septins have been found to interact with the mitotic spindle, the contractile ring, and the midbody, playing a crucial role in the cytokinesis process of metazoan cells (Hartwell et al, 1970; Fededa & Gerlich, 2012; Green et al, 2012; Karasmanis et al, 2019) Mammalian cells express 13 different septins, which can be divided into four groups based on sequence similarity. Three of these septin groups are represented by multiple members, but the fourth group consists only of the single member Septin7 (SEPT7). Members of the four groups assemble into septin octamers, which polymerize to linear, nonpolar polymers. In this structure, septins of the same group can substitute for each other, but SEPT7, as the only member of the fourth group, cannot be substituted by other septins. Therefore, the absence of SEPT7 prevents octamer formation, destabilizes other core septins, and causes loss of functional septin filaments. In the past, we generated and characterized *Sept7* conditional knockout mice (*Sept7*^flox/flox^) (Menon et al, 2014). *Sept7*^Δ/Δ^ embryos were detected in utero only up to embryonic day 6.5 (E6.5)-E7.0 but not later (Menon et al, 2014). SEPT7-deficient fibroblasts and HeLa cells display cytokinetic failure and undergo obligate multinucleation, indicating a crucial role of SEPT7 in cell division (Kremer et al, 2005; Menon et al, 2014). Unexpectedly, lymphocyte-specific targeting of *Sept7 (hCD2-iCre)* (Menon et al, 2014) and myeloid-specific targeting (*Lyz2-Cre*) (Menon et al, 2022) demonstrated no overt abnormality in the development of the blood cell lineages, leading us to the conclusion that SEPT7 is not required for hematopoiesis. However, a later study of *Vav1-iCre*-driven deletion of *Sept7* in hematopoietic stem and progenitor cells (HSPCs), which sit at the apex of the hematopoietic system to form the major blood lineages, demonstrated dramatically reduced engraftment potential along with characteristics of aged hematopoietic stem cells (HSCs) and decreased lymphoid-primed multipotent progenitors in the bone marrow (Kandi et al, 2021). Here, we analyze the role of SEPT7 in hematopoietic cells at different stages of development. We observe that at least one of the two *Sept7* floxed alleles remains resistant to *Vav1-iCre*–mediated recombination, whereas both alleles undergo efficient recombination with *hCD2-iCre*, resulting in efficient *Sept7* knockout in lymphoid cells but not in HSPCs. We demonstrate that SEPT7-positive “escapee” cells emerge already at embryonic day 14.5 and progressively accumulate within the lineage-negative hematopoietic progenitor compartment throughout development, pointing to a strong selective disadvantage of SEPT7-deficient progenitors. Nevertheless, SEPT7 is not essential for cytokinesis in *Hoxb8*-immortalized myeloid and lymphoid progenitors and is not required for lymphoid development under either steady-state or physiological stress induced by oncogenic *K-Ras*–driven tumorigenesis. Our results reveal a stage-specific role of SEPT7 in hematopoiesis, demonstrating that whereas SEPT7 is crucial for early hematopoiesis, it becomes dispensable in later stages.

## Results

### Hematopoietic cells escape *Vav1*-iCre*-*mediated recombination of the *Sept7*^flox/flox^ allele

**Figure 1.**
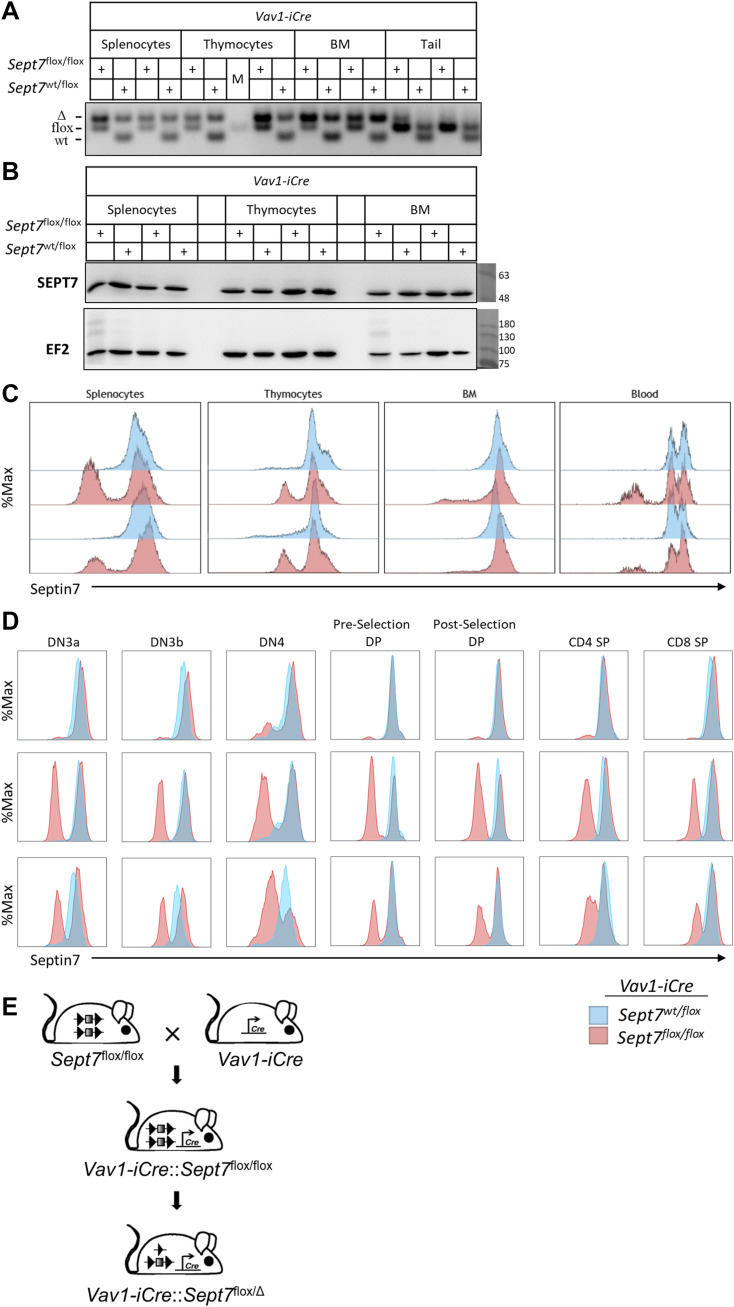
Hematopoietic cells escape *Vav1*-iCre-mediated recombination of *Sept7*^flox/flox^ allele. Splenocytes, thymocytes, bone marrow cells, and peripheral blood cells as well as tail biopsy (Tail) were isolated from *Vav1-iCre*::*Sept7*^wt/flox^ and *Vav1-iCre*::*Sept7*^flox/flox^ littermates. Representative results of three independent experiments involving a total of 19 *Vav1-iCre*::*Sept7*^flox/flox^ and 18 *Vav1-iCre*::*Sept7*^wt/flox^ mice, are shown. **(A)** Genotyping reveals complete excision of *Sept7* floxed allele in *Vav1-iCre*::*Sept7*^wt/flox^ mice and not efficient excision of *Sept7* floxed allele in *Vav1-iCre*::*Sept7*^flox/flox^ mice. Results from two different mice of each genotype are shown. **(B)** Western blot analysis demonstrates expression of SEPT7 in *Vav1-iCre*::*Sept7*^flox/flox^ cells, which are expected to be SEPT7 deficient. The same mice were used as for (A). **(C)** FACS analysis indicates only minor fraction of SEPT7-deficient thymocytes, splenocytes, bone marrow cells and peripheral blood cells isolated from *Vav1-iCre*::*Sept7*^flox/flox^ mice: most cells escape *Vav1-iCre*-induced recombination and express SEPT7. Ten *Vav1-iCre*::*Sept7*^flox/flox^ and nine *Vav1-iCre*::*Sept7*^wt/flox^ mice were analyzed. Data are representative of two mice of each genotype. **(D)** FACS analysis of SEPT7 expression at different stages of T-cell development. The escapees are detected at all stages of T-cell development in *Vav1-iCre*::*Sept7*^flox/flox^ mice. Data from three mice of each genotype are shown. A total of five *Vav1-iCre*::*Sept7*^flox/flox^ and five *Vav1-iCre*::*Sept7*^wt/flox^ mice were analyzed in this experiment. **(E)** Schematic presentation of the experimental results: incomplete excision of *Sept7* floxed allele in hematopoietic cells from *Vav1-iCre*::*Sept7*^flox/flox^ mice. In flow cytometry (FACS) analysis, SEPT7 was detected using an Alexa Fluor 488-conjugated secondary antibody in the B525-A channel. Source data are available for this figure.

**Figure S1.**
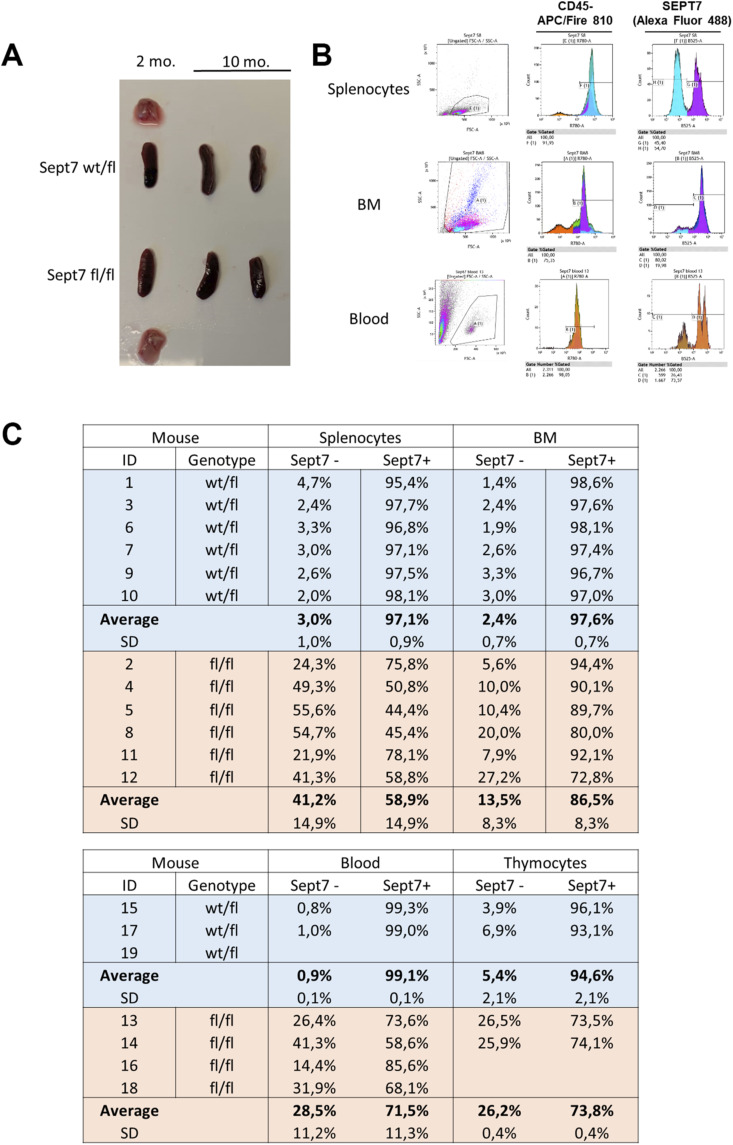
Hematopoietic cells escape *Vav1*-iCre-mediated recombination of *Sept7*^flox/flox^ allele. Representative results from the analysis of 10 *Vav1-iCre::Sept7*^flox/flox^ and nine *Vav1-iCre::Sept7*^wt/flox^ mice are presented. **(A)** Representative images of the spleen and thymus from one 2-mo-old and two 10-mo-old *Vav1-iCre::Sept7*^flox/flox^ (fl/fl) mice, as well as from one 2-mo-old and two 10-mo-old *Vav1-iCre::Sept7*^wt/flox^ (wt/fl) mice, are shown. **(B)** Splenocytes, thymocytes, bone marrow cells, and peripheral blood cells were double-stained for CD45 (R780-A) and SEPT7 (B525-A) and analyzed by FACS. R780-A corresponds to APC/Fire 810 anti-mouse CD45 staining and B525-A to SEPT7 (Alexa Fluor 488). Representative staining of splenocytes, bone marrow, and peripheral blood from one *Vav1-iCre::Sept7*^flox/flox^ mouse is shown. **(C)** Frequencies of Sept7-positive and negative cells. Rows indicate individual mice. Columns indicate populations.

**Figure S2.**
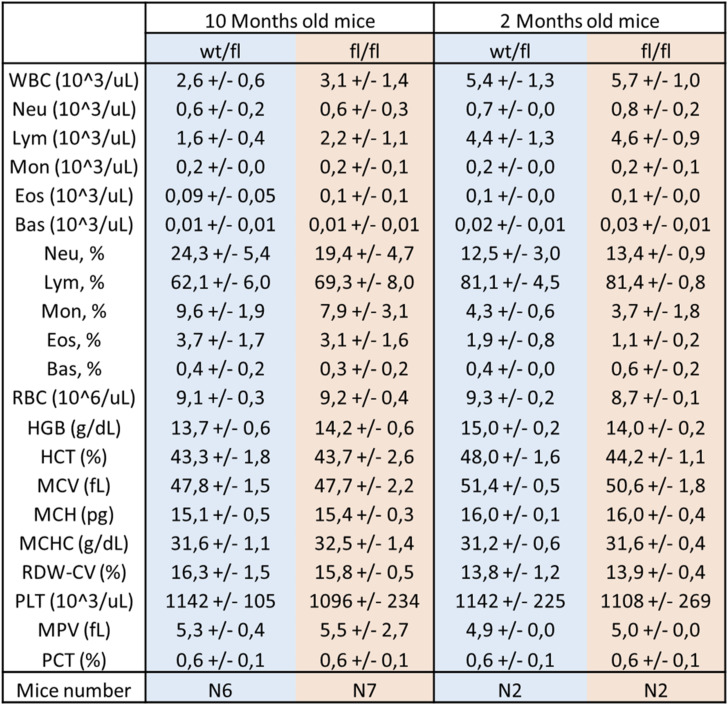
Analysis of peripheral blood from *Vav1-iCre::Sept7*^flox/flox^ vs. *Vav1-iCre::Sept7*^wt/flox^ mice. Peripheral blood samples were analyzed using an Element HT5 Veterinary Hematology Analyzer (Scil animal care company, Antech Diagnostics Germany GmbH). A total of nine *Vav1-iCre::Sept7*^flox/flox^ (fl/fl) mice and eight *Vav1-iCre::Sept7*^wt/flox^ (wt/fl) mice were analyzed, including two 2-mo-old and seven 10-mo-old fl/fl mice, as well as two 2-mo-old and six 10-mo-old wt/fl mice. Data are presented as the mean ± SD.

**Figure S3.**
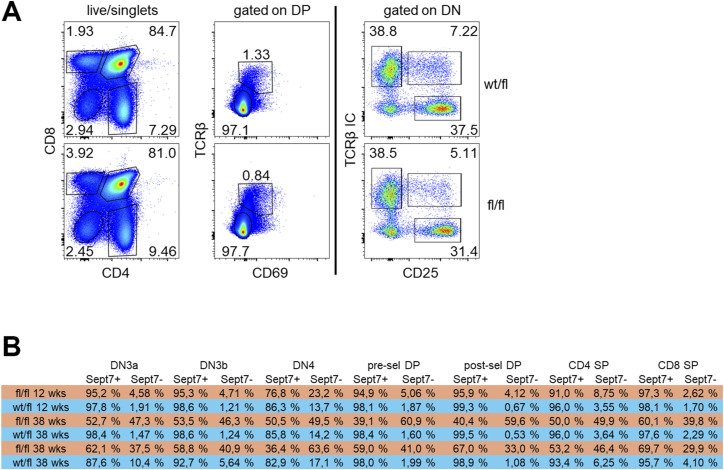
Overall development of T cells from *Vav1-iCre::Sept7*^flox/flox^ mice is unimpaired. Representative results from three independent experiments involving a total of five *Vav1-iCre*::*Sept7*^flox/flox^ and five *Vav1-iCre*::*Sept7*^wt/flox^ mice are shown. **(A)** Thymocytes from 12wk-old mice were analyzed for surface expression of CD4, CD8, TCR**β**, and CD69 (left and center) or CD4, CD8, and CD25 and intracellular expression of TCR**β** (TCR**β** IC, right). Populations are definfed as double-negative (DN), CD4^−^CD8^−^); double-positive (DP), CD4^+^CD8^+^; single-positive (SP), CD4^+^CD8^−^ and CD4^−^CD8^+^; pre-selection DP, TCR**β**-CD69^−^; post-selection DP, TCR**β**+CD69^+^; DN3a, CD25+TCR**β**ic-; DN3b, CD25+TCR**β**ic+; DN4, CD25-TCR**β**ic+. Numbers adjacent to gates indicate frequencies relative to parent gates indicated on top. Representative data from 1 12 wk and 2 38 wk old mice are shown. **(B)** Frequencies of Sept7-positive and negative cells. Rows indicate individual mice with age and genotype. Columns indicate populations.

To establish the role of SEPT7 in hematopoiesis, we aimed to conditionally delete *Sept7* in mice by taking advantage of hematopoiesis-restricted Cre recombinase (Cre) transgene expression in *Vav1*-iCre mice (de Boer et al, 2003). We crossed loxP-flanked *Sept7* (*Sept7*^flox/flox^ or *Sept7*^wt/flox^) mice with *Vav1-iCre* mice. *Vav1-iCre* expresses active Cre in HSPCs and all hematopoietic descendants (de Boer et al, 2003). Genotyping analysis of splenocytes, thymocytes, and bone marrow from *Vav1-iCre*::*Sept7*^wt/flox^ and *Vav1-iCre*::*Sept7*^flox/flox^ mice demonstrated incomplete excision of *Sept7* floxed alleles from *Vav1-iCre*::*Sept7*^flox/flox^ mice (Fig 1A). In contrast, no traces of *Sept7* floxed allele could be detected in *Vav1-iCre*::*Sept7*^wt/flox^ cells, indicating highly effective Cre-mediated excision of *Sept7* floxed allele in the cells, carrying one wild-type allele of *Sept7* (Fig 1A). Western blot analysis of SEPT7 protein expression revealed clearly detectable SEPT7 protein levels in splenocytes, thymocytes, and bone marrow isolated from *Vav1-iCre*::*Sept7*^flox/flox^ mice (Fig 1B). Intracellular SEPT7 protein expression assessed by flow cytometry demonstrated that most splenocytes, thymocytes, bone marrow cells, and peripheral blood cells from *Vav1-iCre*::*Sept7*^flox/flox^ mice still contain SEPT7 (Figs 1C and S1A–C). Analysis of peripheral blood revealed no differences in blood cell composition between *Vav1-iCre::Sept7*^flox/flox^ and *Vav1-iCre::Sept7*^wt/flox^ mice, suggesting that either SEPT7 is dispensable for the maintenance of steady-state hematopoiesis under homeostatic conditions or that hematopoiesis is progressively dominated by cells that have escaped *Sept7* deletion (Fig S2). We, therefore, focused our analysis on intrathymic T-cell development. We hypothesized that periodic strong proliferative bursts followed by cell-cycle inactivity during somatic recombination of antigen-receptor genes might reveal proliferation-driven selection pressure. Overall development of T cells from *Vav1-iCre*::*Sept7*^flox/flox^ mice was unimpaired (Fig S3A). We observed similar proportions of double-negative (DN), double-positive (DP), and single-positive (SP) thymocyte populations and similar frequencies of CD4^+^ and CD8^+^ T cells and B cells within the spleen of *Vav1-iCre*::*Sept7*^wt/flox^ and *Vav1-iCre*::*Sept7*^flox/flox^ mice. Analysis of SEPT7 protein in thymocyte subsets covering stages of T-cell receptor gene rearrangement (DN3a, pre-selection DP), proliferative stages (DN3b, DN4), and selection/maturation stages (post-selection DP) revealed biphasic distribution of SEPT7 in cells from *Vav1-iCre*::*Sept7*^flox/flox^ mice. Thus, cells with incomplete deletion of floxed *Sept7* alleles, referred to as escapees, are present at each developmental stage. However, we note that SEPT7-negative cells are maintained even through stages of high proliferative activity (DN3b and DN4) (Figs 1D and S3B). Considering the complete excision of *Sept7* floxed alleles in *Vav1-iCre*::*Sept7*^wt/flox^ mice, we conclude that hematopoietic cells are strongly driven to evade *Vav1*-iCre-mediated recombination of the *Sept7*^flox/flox^ allele because of intense selection pressure favoring the survival of SEPT7-expressing cells (Fig 1E).

### SEPT7 is effectively eliminated in T cells from *hCD2-iCre*::*Sept7*^flox/flox^ mice

**Figure 2.**
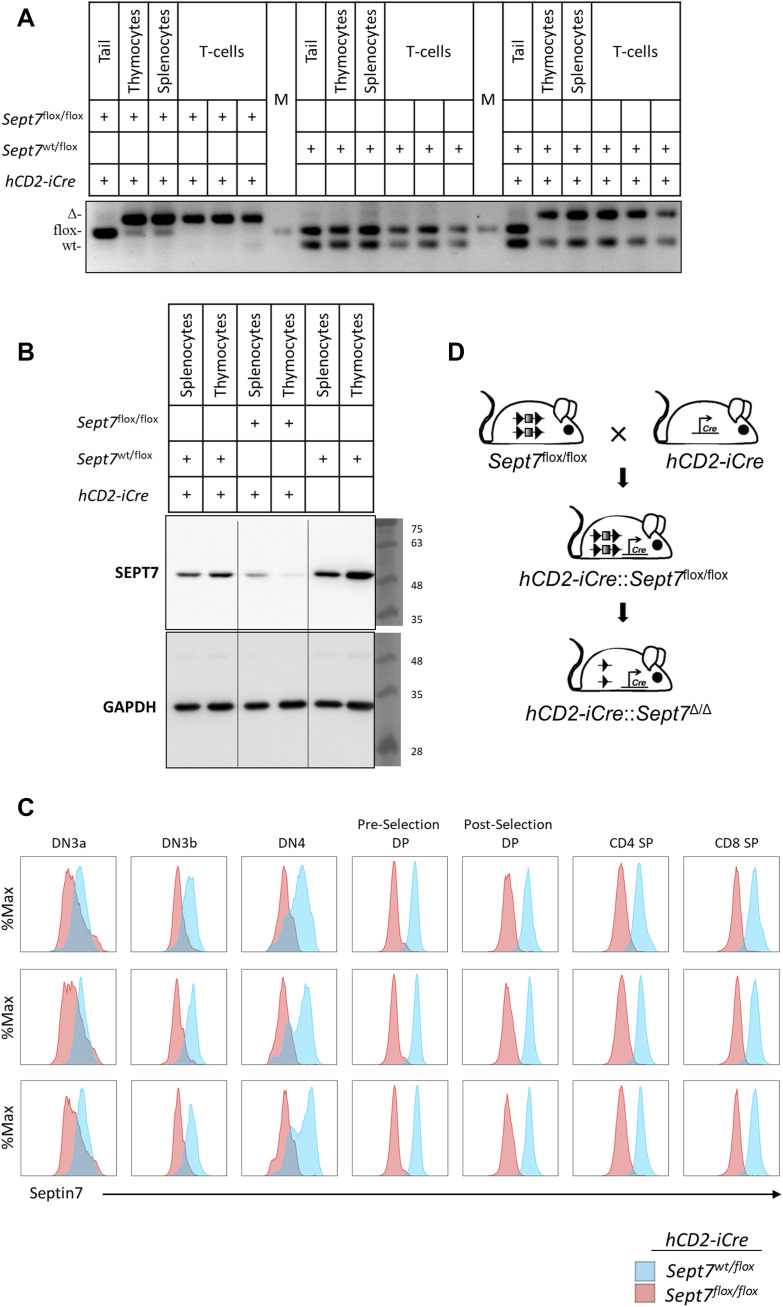
SEPT7 is effectively eliminated in T cells from *hCD2*-iCre:*:Sept7*^flox/flox^ mice. Splenocytes, thymocytes, T cells and tail biopsy (Tail) were isolated from *hCD2-iCre*::*Sept7*^wt/flox^ and *hCD2-iCre*::*Sept7*^flox/flox^ littermates and from *Sept7*^wt/flox^ mice without *hCD2-driven iCre* expression. Representative results of three independent experiments involving a total of eight *hCD2-iCre*::*Sept7*^flox/flox^ and eight *hCD2-iCre*::*Sept7*^wt/flox^ mice are shown. **(A)** Genotyping reveals effective excision of *Sept7* floxed allele in T cells from *hCD2-iCre*::*Sept7*^wt/flox^ and *hCD2-iCre*::*Sept7*^flox/flox^ mice. Data from one mouse per genotype are shown. **(B)** Western blot analysis demonstrates decreased expression of SEPT7 in *hCD2-iCre*::*Sept7*^flox/flox^ thymocytes and splenocytes. Data from one mouse per genotype are shown. **(C)** FACS analysis indicates accumulation of SEPT7-deficient cells during T-cell development. SEPT7 was detected using an Alexa Fluor 488-conjugated secondary antibody in the B525-A channel. Data from three different mice of each genotype are shown. A total of five *hCD2-iCre*::*Sept7*^flox/flox^ and five *hCD2-iCre*::*Sept7*^wt/flox^ mice were analyzed in this experiment. **(D)** Schematic presentation of the experimental results: complete excision of *Sept7* floxed allele in T cells from *hCD2-iCre*::*Sept7*^flox/flox^ mice. Source data are available for this figure.

**Figure S4.**
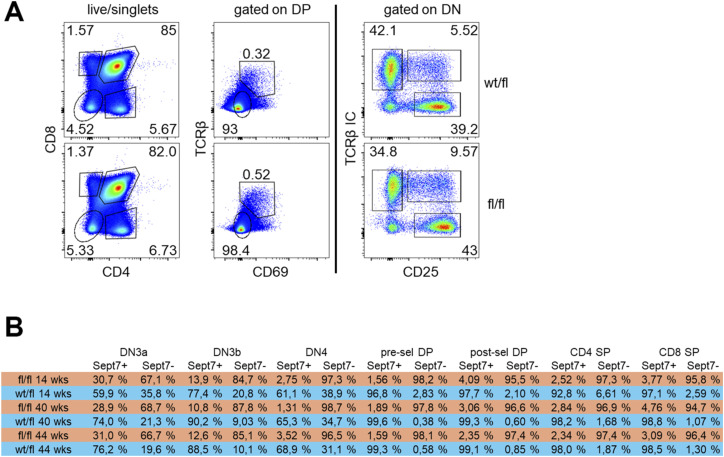
Overall development of T cells from *hCD2-iCre::Sept7*^flox/flox^ mice is unimpaired. Representative results from three independent experiments involving a total of five *hCD2-iCre*::*Sept7*^flox/flox^ and five *hCD2-iCre*::*Sept7*^wt/flox^ mice are shown. **(A)** Thymocytes from 14wk-old mice were analyzed for surface expression of CD4, CD8, TCR**β**, and CD69 (left and center) or CD4, CD8, and CD25 and intracellular expression of TCR**β** (TCR**β** IC, right). Populations are definfed as double-negative (DN), CD4^−^CD8^−^; double-positive (DP), CD4^+^CD8^+^; single-positive (SP), CD4^+^CD8^−^ and CD4^−^CD8^+^; pre-selection DP, TCR**β**-CD69^−^; post-selection DP, TCR**β**+CD69^+^; DN3a, CD25+TCR**β**ic-; DN3b, CD25+TCR**β**ic+; DN4, CD25-TCR**β**ic+. Numbers adjacent to gates indicate frequencies relative to parent gates indicated on top. Representative data from 1 14 wk and 2 40 wk old mice are shown. **(B)** Frequencies of Sept7-positive and negative cells. Rows indicate individual mice with age and genotype. Columns indicate populations.

**Figure S5.**
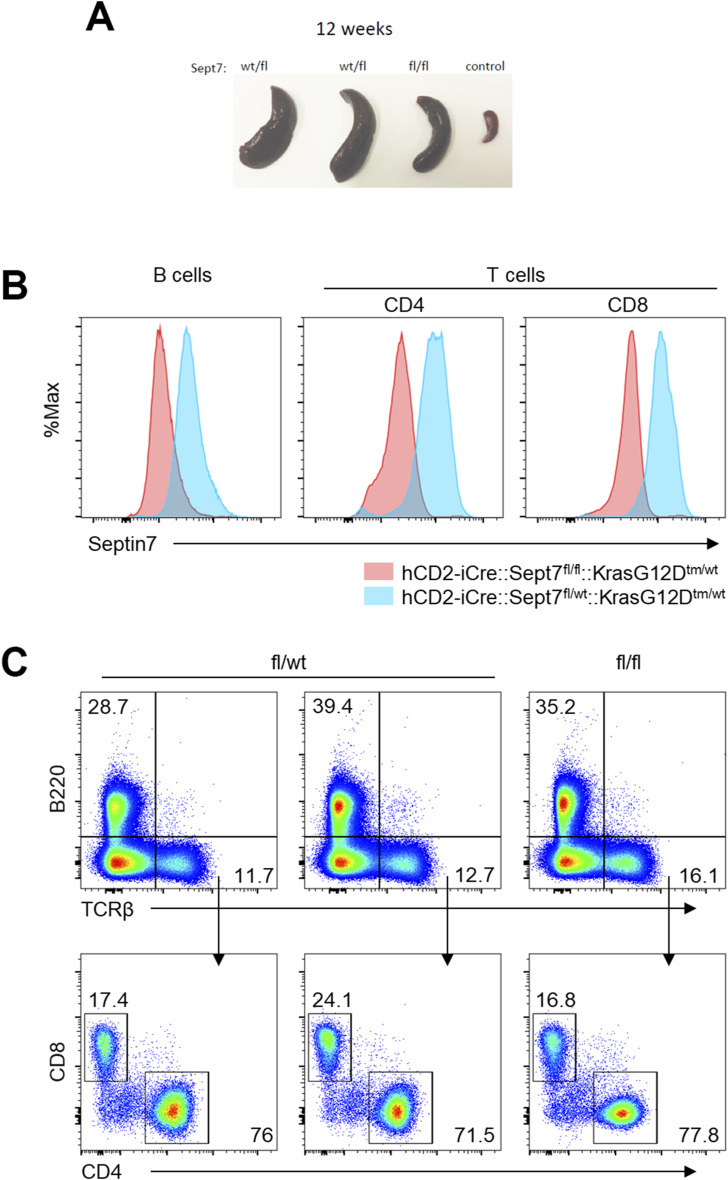
Overall composition of splenic T and B cells from *hCD2-iCre::Sept7*^flox/flox^::*K-RasG12D*^tm/wt^ mice is unimpaired. A total of five *hCD2-iCre::Sept7*^flox/flox^*::K-RasG12D*^tm/wt^ and five *hCD2-iCre::Sept7*^wt/flox^*::K-RasG12D*^tm/wt^ mice were analyzed in two independent experiments. **(A)** Spleens from *K-RasG12D*^*tm/wt*^ mice exhibit splenomegaly in comparison with control spleen without K-RasG12D expression. **(B)** Expression of Septin7 in splenic T and B cells from *hCD2-iCre::Sept7*^flox/flox^*::K-RasG12D*^tm/wt^ and *hCD2-iCre::Sept7*^wt/flox^*::K-RasG12D*^tm/wt^ mice. Each histogram represents data from one individual mouse. **(C)** B cells and T cells are defined as B220+ and TCR**β**+, respectively (upper row). Lower row: T cells were then subdivided according to expression of CD4 or CD8. Numbers adjacent to gates indicate frequencies relative to parent gates indicated on top. Representative data from two individual Septin7-sufficient and one Septin7-deficient mouse are shown.

**Figure S6.**
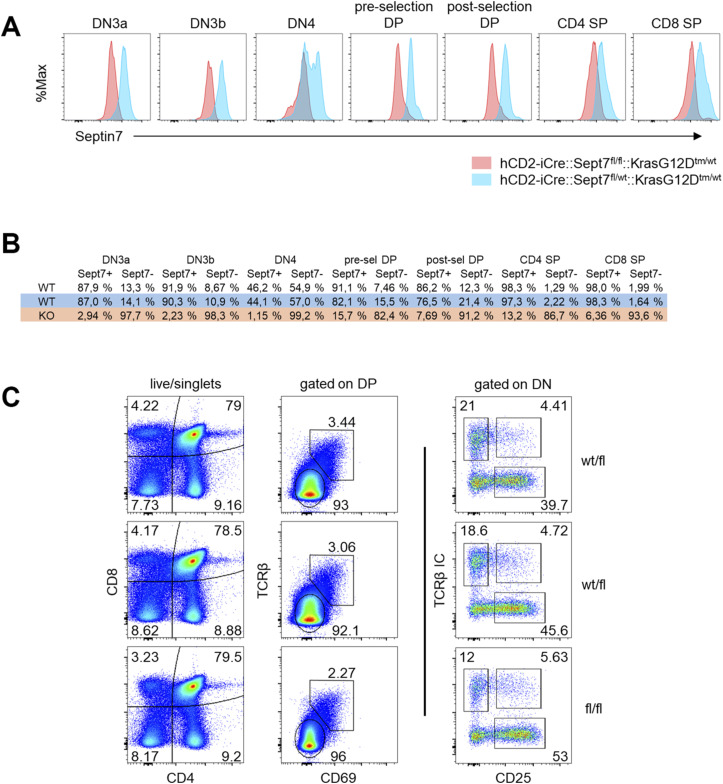
Overall development of T cells from *hCD2-iCre::Sept7*^flox/flox^*::K-RasG12D*^tm/wt^ mice is unimpaired. The same mice as in Fig S5 were analyzed in two independent experiments. **(A)** FACS analysis indicates accumulation of SEPT7-deficient cells during T-cell development. **(B)** Frequencies of Sept7-positive and negative cells. Rows indicate individual mice with genotype. Columns indicate populations. **(C)** Thymocytes were analyzed for surface expression of CD4, CD8, TCR**β**, and CD69 (left and center) or CD4, CD8, and CD25 and intracellular expression of TCR**β** (TCR**β** IC, right). Populations are defined as double-negative (DN), CD4^−^CD8^−^; double-positive (DP), CD4^+^CD8^+^; single-positive (SP), CD4^+^CD8^−^ and CD4^−^CD8^+^; pre-selection DP, TCR**β**-CD69^−^; post-selection DP, TCR**β**+CD69^+^; DN3a, CD25+TCR**β**ic-; DN3b, CD25+TCR**β**ic+; DN4, CD25-TCR**β**ic+. Numbers adjacent to gates indicate frequencies relative to parent gates indicated on top. Representative data from two individual Septin7-sufficient and one Septin7-deficient mouse (the same mice as in Fig S5) are shown.

**Figure S7.**
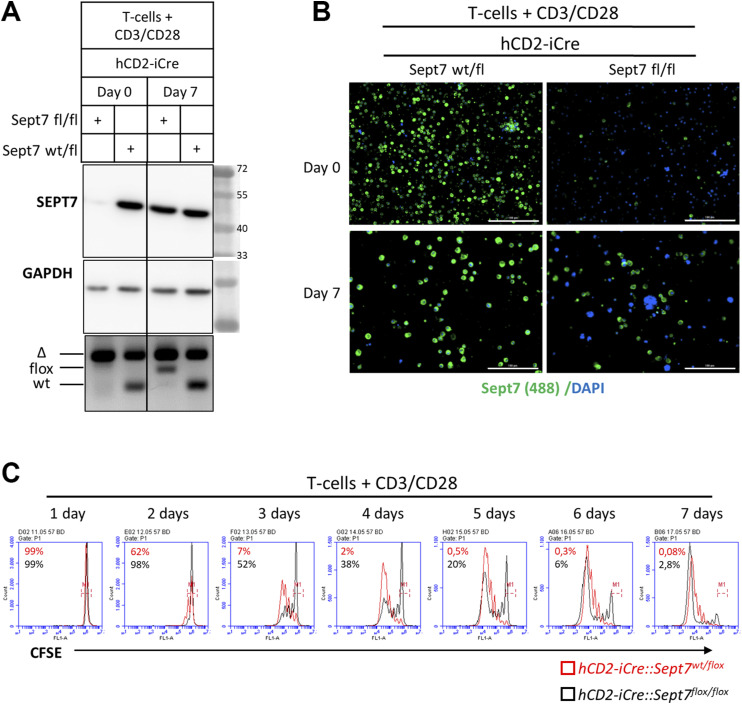
T cells from *hCD2-iCre::Sept7*^flox/flox^ and *hCD2-iCre::Sept7*^wt/flox^ mice were activated ex vivo using Dynabeads Mouse T-Activator CD3/CD28. Representative data from three independent experiments are shown, with one mouse per genotype included in each experiment. **(A)** Upper panel: Western blot analysis shows markedly reduced SEPT7 protein levels in freshly isolated T cells from *hCD2-iCre::Sept7*^flox/flox^ mice compared with *hCD2-iCre::Sept7*^wt/flox^ controls (Day 0). After 7 d of ex vivo activation and expansion, SEPT7 expression was restored to comparable levels in both genotypes (Day 7). Middle panel: GAPDH served as a loading control. Lower panel: Genotyping confirmed efficient excision of the floxed Sept7 allele in freshly isolated T cells from *hCD2-iCre::Sept7*^flox/flox^ mice (Day 0). In contrast, the remaining unexcised floxed allele became readily detectable after 7 d of ex vivo activation and expansion (Day 7), indicating selective outgrowth of SEPT7-expressing T cells. **(B)** Immunofluorescence analysis of SEPT7 expression in T cells shown in (A). Scale bar: 100 μm. **(C)** T-cell proliferation was assessed over 7 d by flow cytometric analysis of CFSE dilution. Gate M1 indicates the percentage of undivided cells.

To assess whether SEPT7-negative cells are indeed capable of undergoing T-cell development, we generated mice in which Cre-mediated excision of *Sept7* was restricted to T and B cells. We crossed loxP-flanked *Sept7* (*Sept7*^flox/flox^ or *Sept7*^wt/flox^) mice with *hCD2-iCre* mice. *hCD2-iCre* expresses active Cre in immature and mature B and T lymphocytes beginning at the common lymphoid progenitor stage (de Boer et al, 2003). Genotyping analysis of splenocytes, thymocytes, and T cells from *hCD2-iCre*::*Sept7*^wt/flox^ and *hCD2-iCre*::*Sept7*^flox/flox^ mice demonstrated effective excision of *Sept7* floxed alleles in T cells of *hCD2-iCre*::*Sept7*^flox/flox^ mice (Fig 2A). Western blot analysis of SEPT7 protein expression revealed significantly decreased SEPT7 protein levels in splenocytes and thymocytes of *hCD2-iCre*::*Sept7*^flox/flox^ when compared with *hCD2-iCre*::*Sept7*^wt/flox^ mice or to the *Sept7*^flox/flox^ mice without Cre expression (Fig 2B). However, we observed no differences in T-cell differentiation between *hCD2-iCre*::*Sept7*^flox/flox^ and *hCD2-iCre*::*Sept7*^wt/flox^ in the thymus, as all thymocyte populations were comparable (Fig S4A). Nevertheless, subtle changes in total cellularity may be masked when analyzing percentages alone. Flow cytometric analysis demonstrated that the depletion of SEPT7 at the DN2/DN3a stage was not complete (Figs 2C and S4B). In DN3b thymocytes the SEPT7 protein seems to be largely absent, and even less SEPT7 could be detected in DN4, although both populations proliferate strongly (Figs 2C and S4B). In DP cells, a small proportion of *hCD2-iCre*::*Sept7*^flox/flox^ cells were SEPT7-positive before selection, and a few CD4 SP and CD8 SP cells may have retained SEPT7 expression as assessed by flow cytometry as well (Figs 2C and S4B). The significantly more efficient deletion of the *Sept7* gene from the DP stage onward can be explained by the progressive nature of Cre-mediated recombination during T-cell development, beginning at the common lymphoid progenitor stage. Given that upon virtually complete deletion at the DN3b stage of T-cell development, no substantial frequencies of escapees emerged in this experimental model, we conclude that SEPT7 is not essential for proliferation during T-cell development at and after the DN3b stage (Figs 2D and S4B). DN3b and DN4 thymocytes are highly proliferative under physiological conditions; nevertheless, SEPT7 deficiency does not impair their expansion, indicating that SEPT7 is dispensable for proliferation at these stages. Otherwise, T cell development would be expected to result in strong selective pressure favoring SEPT7-positive cells, which we do not observe. Our observations are consistent with previous studies demonstrating that intrathymic development of SEPT7-deficient T cells remains largely unaffected (Menon et al, 2014; Mujal et al, 2016). Because our conclusion that SEPT7 is dispensable for T-cell development was based on steady-state observations, we next sought to evaluate the role of SEPT7 in T- and B-cell development under conditions of physiological stress. To mimic physiological stress in lymphoid progenitors, we used a mouse model with selective activation of oncogenic *K-Ras* in *hCD2*-iCre–expressing cells. Expression of oncogenic *K-Ras*-G12D in *hCD2*-iCre–expressing cells induced splenomegaly in both *K-Ras-G12D::hCD2-iCre::Sept7*^flox/flox^ and *K-Ras-G12D::hCD2-iCre::Sept7*^wt/flox^ mice (Fig S5A). Nevertheless, *K-Ras-G12D::hCD2-iCre::Sept7*^flox/flox^ mice showed efficient depletion of SEPT7 in both T and B cells (Figs S5B and S6A and B). However, we did not observe any differences in T- or B-cell development between *K-Ras-G12D::hCD2-iCre::Sept7*^flox/flox^ and *K-Ras-G12D::hCD2-iCre::Sept7*^wt/flox^ mice, neither in the thymus nor in the spleen (Figs S5C and S6C). This observation led us to conclude that SEPT7 is not essential for lymphoid cell development, even under conditions of physiological stress. In contrast, ex vivo proliferation assay following stimulation with T-cell activator CD3/CD28 revealed that T-cell expansion depends on SEPT7 expression. Compared with *hCD2-iCre::Sept7*^wt/flox^ controls, *hCD2-iCre::Sept7*^flox/flox^ T cells displayed delayed proliferation. PCR genotyping, Western blot analyses, and immunofluorescence staining demonstrated that this impaired expansion was associated with the selective outgrowth of SEPT7-expressing cells (Fig S7A–C). These findings are consistent with the study by Mujal et al (2016), which demonstrated that SEPT7 is required for cytokine-driven autonomous ex vivo T-cell proliferation but is dispensable for contact-dependent division mediated by antigen-presenting cells.

### *Hoxb8*-immortalized HSPCs from *Vav1-iCre*::*Sept7*^flox/flox^ mice escape Cre-mediated recombination and express SEPT7

**Figure 3.**
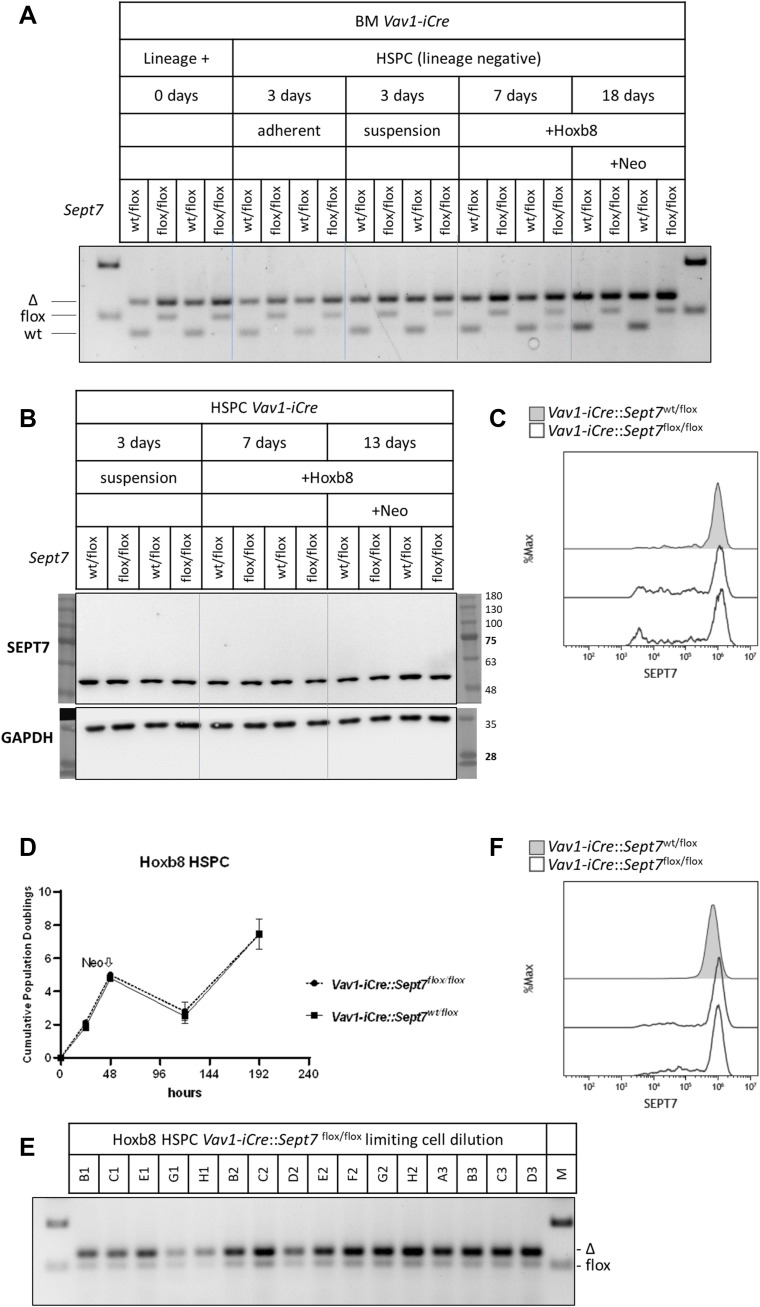
HSPCs escape *Vav1*-iCre-mediated recombination of *Sept7*^flox/flox^ alleles. Lineage-negative bone marrow cells were isolated from *Vav1-iCre*::*Sept7*^wt/flox^ and *Vav1-iCre*::*Sept7*^flox/flox^ littermates. **(A, B, C, D)** represent the experiments with HSPCs isolated from two different mice of each genotype. Cells were stably transduced with *Hoxb8* expressing construct that promotes myeloid progenitor proliferation. Representative results of three independent experiments with a total of four mice per genotype are shown. **(A)** Genotyping reveals complete excision of *Sept7* floxed from *Vav1-iCre*::*Sept7*^wt/flox^ cells and inefficient excision of *Sept7* floxed allele in *Vav1-iCre*::*Sept7*^flox/flox^ cells. **(B)** Western blot analysis demonstrates expression of SEPT7 in *Vav1-iCre*::*Sept7*^flox/flox^ cells, which are expected to be SEPT7 deficient. **(C)** FACS analysis indicates SEPT7 expression in all *Hoxb8*-immortalized HSPCs derived from *Vav1-iCre*::*Sept7*^flox/flox^ mice. **(D)** *Hoxb8*-immortalized HSPCs from *Vav1-iCre*::*Sept7*^flox/flox^ mice demonstrate proliferation rate comparable with *Vav1-iCre*::Sep7^wt/flox^ cells. **(E)** Genotyping analysis of single cell colonies derived from *Hoxb8*-immortalized *Vav1-iCre*::*Sept7*^flox/flox^ HSPCs demonstrates incomplete recombination of *Sept7* floxed allele. **(F)** FACS analysis confirms SEPT7 expression in two random clones (C1 and C3) of *Hoxb8*-immortalized *Vav1-iCre*::*Sept7*^flox/flox^ HSPCs. In flow cytometry (FACS) analysis, SEPT7 was detected using an Alexa Fluor 488-conjugated secondary antibody in the B525-A channel. HSPC, hematopoietic stem and progenitor cells. Source data are available for this figure.

To generate a stably growing, homogenous hematopoietic progenitor cell population, we immortalized early HSPCs from *Vav1-iCre*::*Sept7*^flox/flox^ and *Vav1-iCre*::*Sept7*^wt/flox^ mice by gammaretroviral delivery of estrogen-regulated *Hoxb8* (Redecke et al, 2013). Monitoring of Cre-mediated recombination in these hematopoietic cells by genotyping revealed that *Vav1-iCre*::*Sept7*^flox/flox^ cells had incomplete recombination of the *Sept7* floxed alleles, suggesting the presence of cells that had retained at least one functional *Sept7* allele (Fig 3A). No traces of *Sept7* floxed allele could be detected in *Vav1-iCre*::*Sept7*^wt/flox^ cells, indicating highly effective Cre-mediated excision of the *Sept7* floxed allele in the cells, where cell growth was protected by the presence of wild-type allele of *Sept7* (Fig 3A). Western blot analysis of SEPT7 protein expression indicated comparable SEPT7 protein levels in HSPCs of both genotypes (Fig 3B). Flow cytometry analysis of intracellular SEPT7 protein expression revealed that the *Hoxb8*-immortalized HSPCs derived from *Vav1-iCre*::*Sept7*^flox/flox^ HSPCs express SEPT7 (Fig 3C). Population doubling time of *Sept7*^wt/flox^ and *Sept7*^flox/flox^ cells was comparable (Fig 3D). We also performed limiting cell dilution of *Vav1-iCre*::*Sept7*^flox/flox^ cells to track the fate of individual cells. A total of 16 clones were genotyped after 2–3 wk of propagation, and the results confirmed that all retained the *Sept7* floxed allele (Fig 3E). Flow cytometry analysis in two random clones (C1 and C3) demonstrates that these clones express SEPT7 (Fig 3F). These results imply a critical role of SEPT7 for stem cell maintenance, and this conclusion is strongly supported by our recent study where HSPCs from *Vav1-iCre*::*Sept7*^flox/flox^ mice showed strongly impaired repopulation activity upon transplantation and failed to establish donor chimerism (Kandi et al, 2021).

### The *Sept7* floxed allele is effectively protected from Cre-mediated excision in *Vav1-iCre*::*Sept7*^flox/flox^ *Hoxb8*-immortalized HSPCs

**Figure 4.**
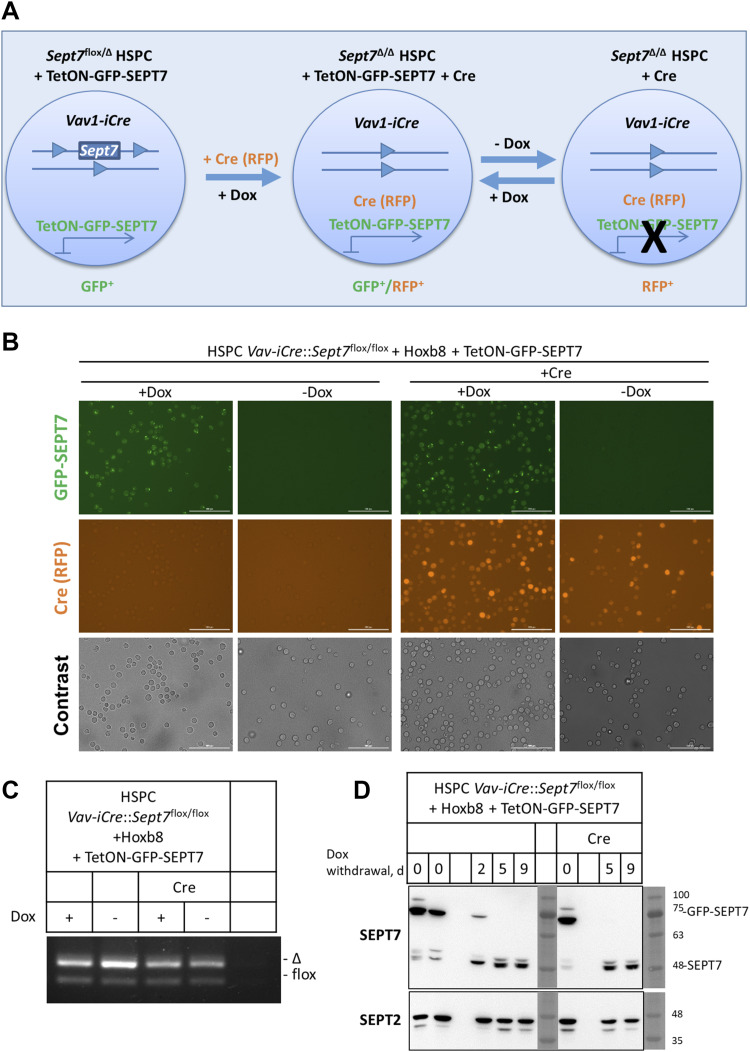
HSPCs from *Vav1-iCre::Sept7*^flox/flox^ mice become protected from Cre-mediated recombination. *Hoxb8*-immortalized HSPCs derived from *Vav1-iCre*::*Sept7*^flox/flox^ mice (Fig 3) were double transduced first by doxycycline-inducible GFP-*Sept7* and subsequently by Cre (RFP) expressing construct. Presence of doxycycline induces GFP-SEPT7 expression, whereas absence of doxycycline stops GFP-SEPT7 expression. GFP/RFP double-positive cells were sorted by FACS. Representative results of two independent experiments using HSPCs derived from two different mice (Fig 3) are shown. **(A)** Schematic representation of the GFP-SEPT7 and Cre (RFP) rescue model. **(B)** Immunofluorescence analysis of single- and double-transduced HSPCs showing dox-inducible GFP-SEPT7 and RFP (Cre) signals. Scale bar: 100 μm. **(C)** Genotyping analysis of single- and double-transduced HSPCs in the presence (+Dox) and absence (-Dox) of doxycycline indicates incomplete excision of *Sept7* floxed allele. **(D)** Immunoblot analysis showing the doxycycline-induced GFP-SEPT7 expression in the single- or double-transduced HSPCs. Upon dox deprivation GFP-SEPT7 disappears and endogenous SEPT7 protein levels are increased. SEPT2 was used as a loading control. HSPC, hematopoietic stem and progenitor cells. Source data are available for this figure.

Because the *Sept7* floxed allele is not efficiently recombined in HSPCs from *Vav1-iCre*::*Sept7*^flox/flox^ mice, possibly because of selection pressure against SEPT7 deficiency in early hematopoiesis, we decided to rescue the *Hoxb8*-immortalized cells by introducing doxycycline-inducible GFP-*Sept7*. We supposed that rescue of cells with a SEPT7-expressing vector construct will release mechanisms of protection from Cre-mediated recombination and will result in effective recombination of *Sept7* floxed allele (Fig 4A). Subsequently, we treated the cells with lentiviral vector particles encoding for Cre and red fluorescent protein (RFP) as a marker gene (Fig 4A). RFP/GFP double-positive *Hoxb8*-immortalized *Vav1-iCre*::*Sept7*^flox/flox^ HSPCs were FACS sorted and subsequently cultivated with or without doxycycline to induce or to stop GFP-*Sept7* expression, respectively (Fig 4B). We did not detect any improvement of recombination and excision of the floxed *Sept7* allele in RFP/GFP double-positive *Hoxb8*-immortalized *Vav1-iCre*::*Sept7*^flox/flox^ HSPCs (Fig 4C). Monitoring of SEPT7 expression by Western blot confirms expression of exogenous GFP-SEPT7 as well as endogenous SEPT7 even after exogenous expression of active Cre (Fig 4D). These results clearly indicate that HSPCs from *Vav1-iCre*::*Sept7*^flox/flox^ mice have developed effective mechanisms to protect at least one of the two *Sept7* floxed alleles from Cre-mediated excision. We suppose that SEPT7 is essential at the early stages of hematopoiesis, as its deletion at later stages of hematopoiesis (e.g., using *hCD2-iCre*) proves to be more effective. The resulting strong selection pressure likely drives cells to evade Cre-mediated recombination, possibly via recombinase-induced DNA methylation (He & Ecker, 2015; Liu et al, 2021). Interestingly, the expression of exogenous SEPT7 in *Hoxb8*-immortalized HSPCs reduces endogenous SEPT7 protein levels, likely because of reduced protein stability of SEPT7 monomers which could not combine to heteromeric septin complexes for stoichiometric reasons (Fig 4D).

### Generation of *Hoxb8*-immortalized *Sept7* KO HSPCs

**Figure 5.**
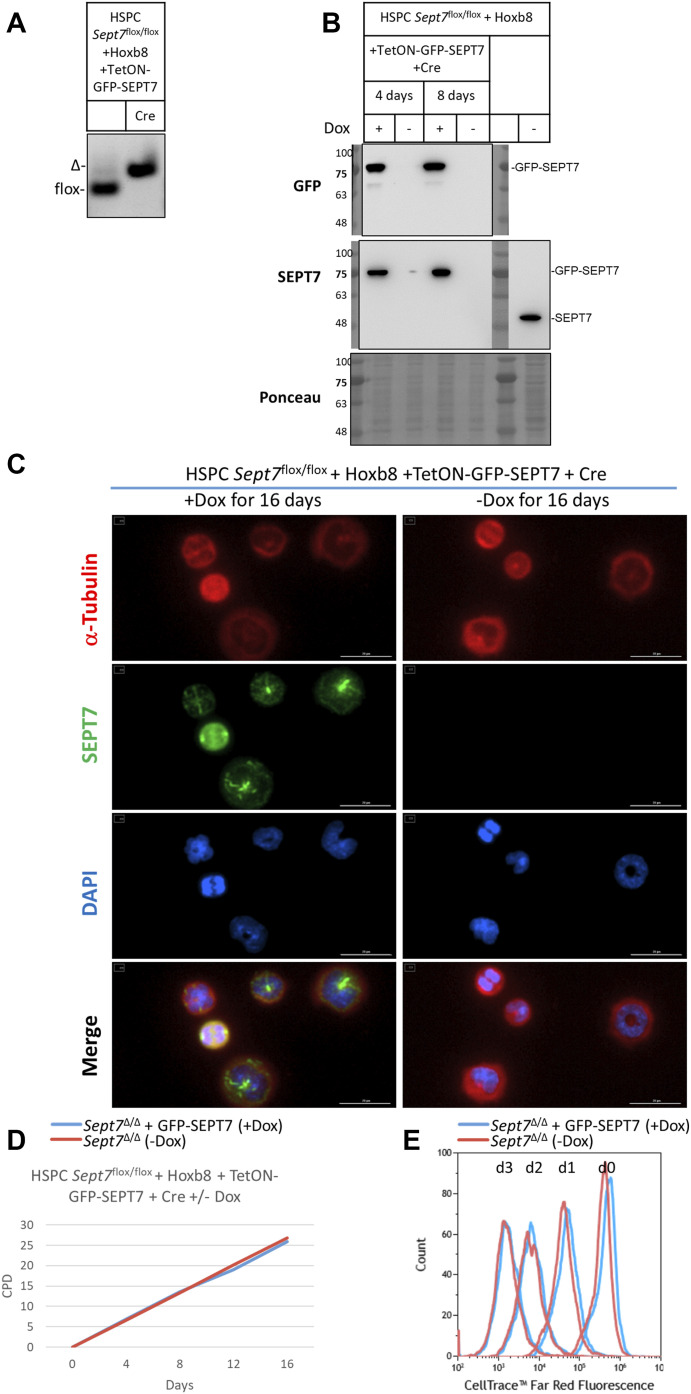
Generation of SEPT7-deficient *Hoxb8*-immortalized HSPCs. *Hoxb8*-immortalized *Sept7*^flox/flox^ HSPCs were double transduced first by doxycycline-inducible GFP-SEPT7 and subsequently by Cre (RFP) expressing construct. Presence of doxycycline induces GFP-SEPT7 expression and absence of doxycycline stops GFP-SEPT7 expression. GFP/RFP double-positive cells were sorted by FACS. Representative results of two independent experiments with HSPCs isolated from two different *Sept7*^flox/flox^ mice (one mouse per experiment) are shown. **(A)** Genotyping analysis of single- and double-transduced HSPCs in the presence of doxycycline indicates complete excision of *Sept7* floxed allele upon Cre expression. **(B)** Immunoblot analysis showing the doxycycline-induced GFP-SEPT7 expression in the double-transduced HSPCs. Upon dox deprivation for 4 or 8 d GFP-SEPT7 disappears and cells become negative for endogenous SEPT7, suggesting Cre-mediated excision of the floxed *Sept7* alleles. Original not transduced *Sept7*^flox/flox^ cells were used as a control. Ponceau staining was used as a loading control. **(C)** Immunofluorescence analysis of double-transduced HSPC demonstrating effective depletion of SEPT7 upon dox deprivation. Scale bar: 20 μm. **(D)** Cumulative population doublings based on cell counting demonstrate comparable proliferation rate of SEPT7-deficient (-Dox) and GFP-SEPT7–expressing (+Dox) *Hoxb8*-immortalized HSPCs. **(E)** Kinetics of proliferation of *Sept7*-deficient (*Sept7*^Δ/Δ^) and GFP-*Sept7–*expressing (*Sept7*^Δ/Δ^ + GFP-*Sept7*) *Hoxb8*-immortalized HSPCs monitored by Far Red dye dilution. Live cells were covalently labeled by Far Red Cell Tracer and each cell division resulted in half the fluorescence intensity of its parent cell. Far Red content was measured by flow cytometry at day 0, 1, 2, and 3 of cells propagation. HSPC, hematopoietic stem and progenitor cells. Source data are available for this figure.

Because the HSPCs from *Vav1-iCre*::*Sept7*^flox/flox^ mice were protected from recombination in vivo, we decided to test the recombination in HSPCs from *Sept7*^flox/flox^ mice ex vivo using the lentiviral Cre delivery as described above. To account for the possibility that SEPT7 is indispensable for HSPC proliferation, we decided to protect the cells with ectopic expression of SEPT7. The lineage-negative cells isolated from the bone marrow of *Sept7*^flox/flox^ mice were first immortalized by gammaretroviral delivery of an estrogen-regulated form of *Hoxb8*. The *Hoxb8*-immortalized *Sept7*^flox/flox^ HSPCs were further transduced with a gammaretroviral vector harboring a dox-inducible GFP-SEPT7 expression cassette. These cells were then treated with lentiviral particles to deliver Cre recombinase together with an RFP marker. In the double-transduced cells, Cre expression leads to the deletion of the endogenous *Sept7* allele, and these knockout cells could be specifically monitored by RFP fluorescence. We sorted RFP/GFP double-positive cells in the presence of doxycycline by FACS. Genotyping of GFP/RFP double-positive cells reveals effective recombination of *Sept7* floxed alleles (Fig 5A). Monitoring of SEPT7 expression by Western blot (Fig 5B) and by immunostaining (Fig 5C) confirmed effective depletion of SEPT7 protein. Surprisingly, we did not observe any significant difference in population doubling time between GFP-SEPT7–expressing (+Dox) and SEPT7-deficient HSPCs monitored by cell counting (Fig 5D) or by flow cytometry (Fig 5E), indicating that SEPT7 is non-essential for *Hoxb8*-immortalized HSPCs propagation ex vivo.

**Figure 6.**
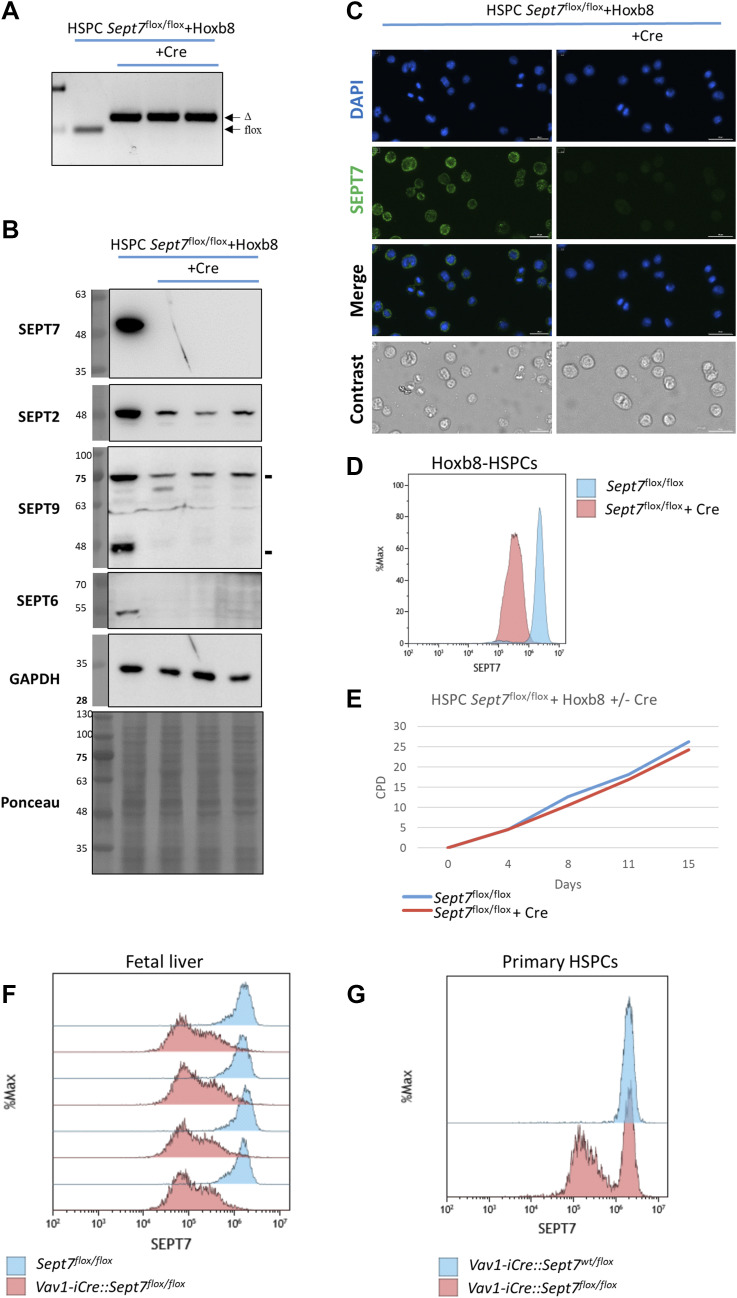
SEPT7 is non-essential for *Hoxb8*-immortalized HSPCs. *Hoxb8*-immortalized *Sept7*^flox/flox^ HSPCs (Fig 5) were transduced with the lentiviral bidirectional construct, co-expressing Cre and mCherry and sorted for mCherry-positive cells by FACS. Representative results of two independent experiments using HSPCs derived from two different mice (Fig 5) are shown. **(A)** Genotyping analysis indicates complete excision of *Sept7* floxed allele upon Cre expression. **(B)** Immunoblot analysis demonstrates that Cre expression leads to effective excision of *Sept7* floxed allele. Elimination of endogenous SEPT7 is accompanied by strong reduction in SEPT2, SEPT6 and SEPT9 levels. GAPDH and Ponceau staining were used as a loading control. **(C)** Immuno-fluorescent analysis confirms effective depletion of SEPT7 in *Sept7*^flox/flox^ HSPCs transduced with Cre. Scale bar: 20 μm. **(D)** FACS analysis confirms effective depletion of SEPT7 in *Sept7*^flox/flox^ *Hoxb8*-immortalized HSPC stably transduced with Cre. **(E)** Cumulative population doublings based on cell counting demonstrate comparable proliferation rate of SEPT7-deficient (*Sept7*^flox/flox^ + Cre) and SEPT7 expressing (*Sept7*^flox/flox^) *Hoxb8*-immortalized HSPCs. **(F)** Expression of Septin7 in CD45-positive fetal liver cells. Data are shown for eight E14.5 embryos (four embryos per genotype) obtained from a single pregnant mouse. Each histogram represents an individual embryo. In total, 13 embryos (seven *Vav1-iCre*::*Sept7*^flox/flox^ and six *Sept7*^flox/flox^) from two pregnant mice were analyzed. **(G)** Expression of Septin7 in primary HSPCs from Vav1-iCre::*Sept7*^flox/flox^ and *Vav1*-iCre::*Sept7*^wt/flox^ mice. In flow cytometry (FACS) analysis, SEPT7 was detected using an Alexa Fluor 488-conjugated secondary antibody in the B525-A channel. HSPC, hematopoietic stem and progenitor cells. Source data are available for this figure.

**Figure S8.**
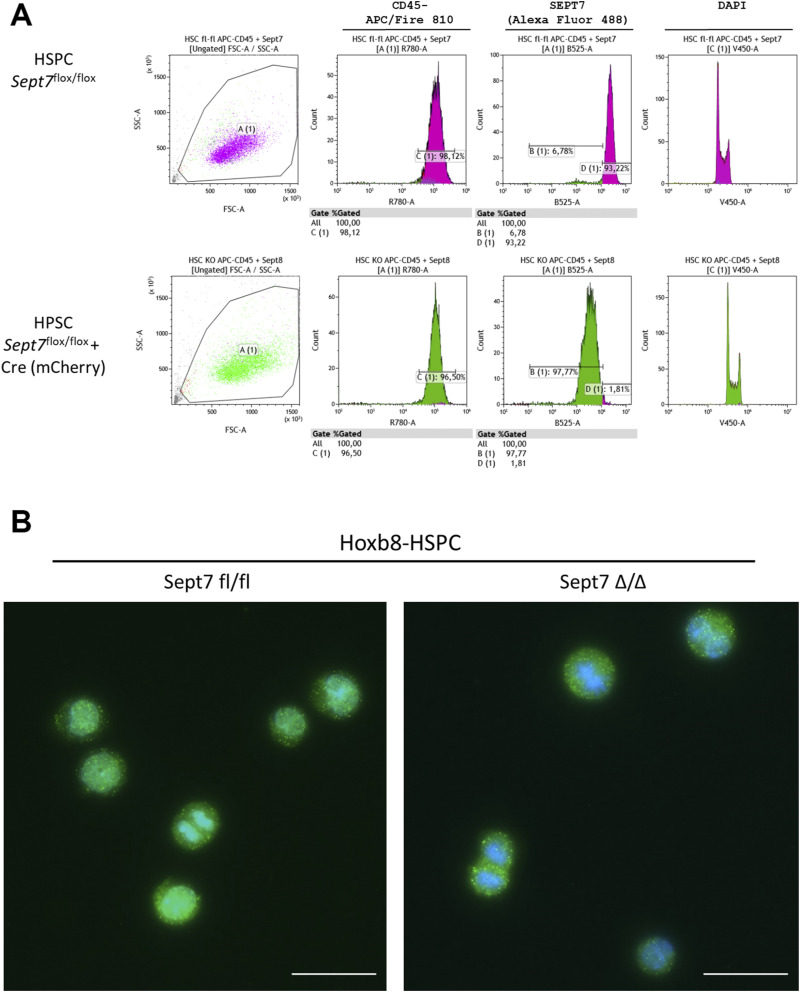
SEPT7 and CDC42 expression in Hoxb8-immortalized HSPCs. Representative results of two independent experiments with HSPCs prepared from two different *Sept7*^flox/flox^ mice (one mouse per experiment) are shown. The same cells as in Fig 6 were used. **(A)** FACS analysis indicates efficient depletion of SEPT7 in *Sept7*^flox/flox^ Hoxb8-HSPCs transduced with a Cre-expressing construct. R780-A corresponds to APC/Fire 810 anti-mouse CD45 staining, B525-A to SEPT7 (Alexa Fluor 488), and V450-A to DAPI nuclear staining. **(B)** Hoxb8-immortalized SEPT7-deficient (Sept7 Δ/Δ) and SEPT7-expressing control (Sept7 flox/flox) cells were stained for the polarity marker CDC42 (green). Nuclei were counterstained with DAPI (blue). Scale bar: 20 μm. HSPC, hematopoietic stem and progenitor cells.

To exclude any possibility of leakage of dox-inducible GFP-SEPT7 in the absence of doxycycline, we decided to produce SEPT7-deficient HSPCs without prior reconstitution of the cells with SEPT7-expressing constructs. For that, we transduced *Hoxb8*-immortalized HSPCs from *Sept7*^flox/flox^ mice with a lentiviral vector expressing mCherry and Cre from a bidirectional constitutive promoter (pLBid.pA.CTE.nlsCre.minCMV.SF.mCherry.PRE) (Maetzig et al, 2010). In the transduced cells, Cre expression leads to the deletion of the endogenous *Sept7* allele, and these knockout cells could be specifically monitored by mCherry fluorescence. We sorted mCherry-positive cells expressing active Cre recombinase and confirmed efficient excision of *Sept7* floxed allele by genotyping (Fig 6A). In Cre-expressing cells, SEPT7 was not detectable in Western blot (Fig 6B), immunostaining (Fig 6C), or flow cytometry (Figs 6D and S8A). Consistent with previous reports (Menon et al, 2014), loss of SEPT7 is accompanied by a significant reduction in septins from all three remaining septin subgroups—SEPT2, SEPT6, and SEPT9 (Fig 6B)—indicating that SEPT7 function is not compensated by other septins. Population doubling time of *Hoxb8*-immortalized *Sept7* KO HSPCs was comparable with the original *Sept7*^flox/flox^ cells, confirming dispensability of *Sept7* for ex vivo proliferation of *Hoxb8*-HSPCs (Fig 6E). Previous studies have shown that *Hoxb8*-immortalized HSPCs lose self-renewal capacity and megakaryocyte/erythroid differentiation potential when retaining myeloid and lymphoid potential (Redecke et al, 2013). The self-renewal capacity of HSCs is closely linked to cell polarity, which is characterized by the asymmetric distribution of polarity-associated proteins such as CDC42 (Florian et al, 2018). Consistent with these findings, both SEPT7-deficient and SEPT7-expressing *Hoxb8*-immortalized HSPCs exhibited a diffuse distribution of CDC42, including during cell division (Fig S8B). Based on these observations, we propose that although SEPT7 is critical for maintaining cell polarity and HSC function (Kandi et al, 2021), it is non-essential for the proliferation of unpolarized progenitors such as *Hoxb8*-immortalized HSPCs.

**Figure S9.**
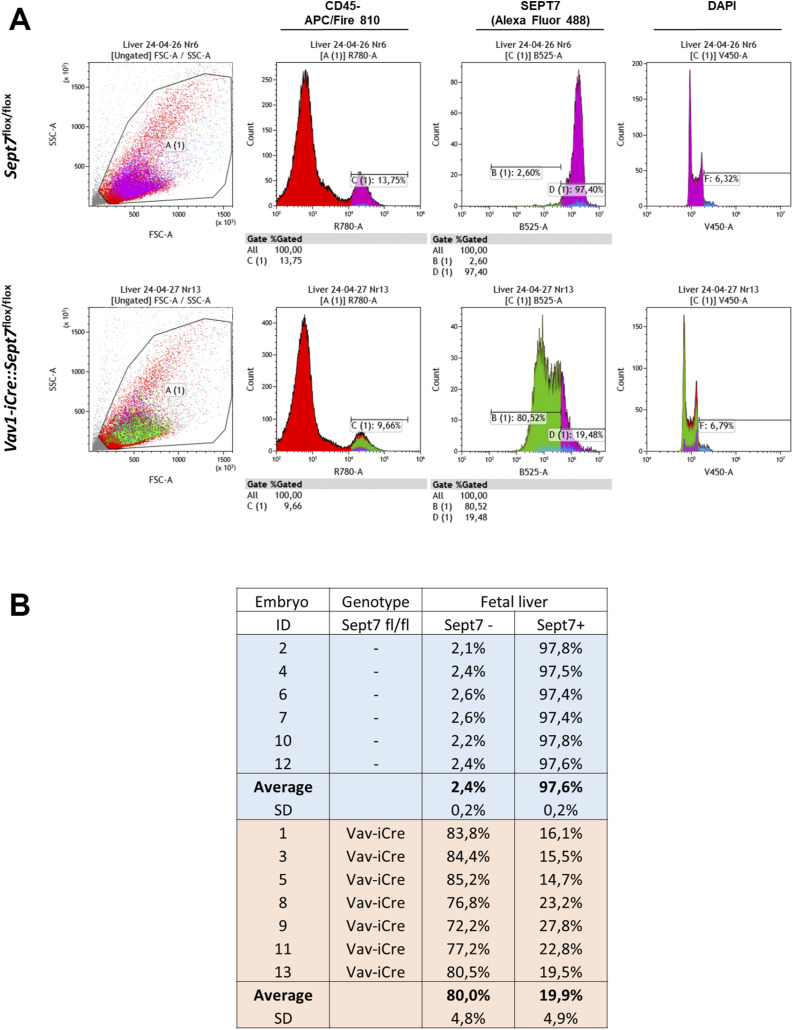
SEPT7 expression in fetal liver. Representative data from two independent experiments, each including one pregnant mouse, are shown. **(A)** FACS analysis indicates incomplete excision of SEPT7 in hematopoietic (CD45-positive) fetal liver cells expressing *Vav1*-iCre. R780-A corresponds to APC/Fire 810 anti-mouse CD45 staining, B525-A to SEPT7 (Alexa Fluor 488), and V450-A to DAPI nuclear staining. Representative staining of one embryo per genotype is shown. **(B)** Frequencies of SEPT7-positive and SEPT7-negative cells in fetal liver. Rows represent individual embryos, and columns represent cell populations. Data were obtained from 13 E14,5 embryos from two pregnant mice, generated by crossing two *Sept7*^flox/flox^ females with two *Vav1-iCre*^tm/wt^::*Sept7*^flox/flox^ males.

**Figure S10.**
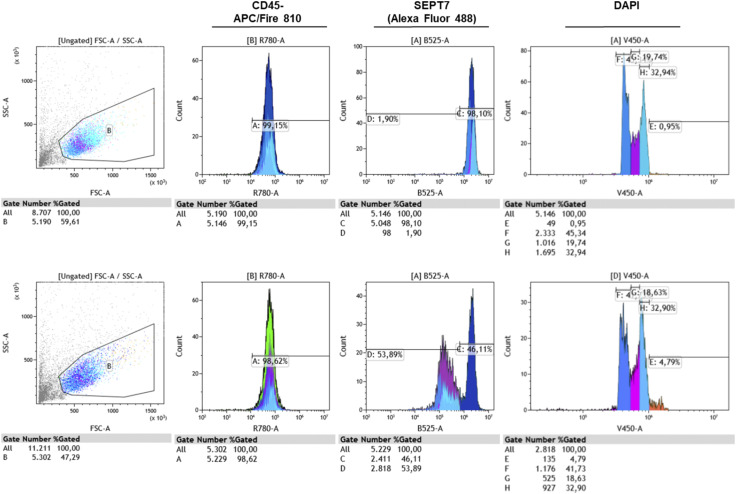
SEPT7 expression in primary HSPCs. Representative data from two independent experiments are shown, each including one *Vav1-iCre::Sept7*^flox/flox^ and one *Vav1-iCre::Sept7*^wt/flox^ mouse. FACS analysis indicates incomplete excision of SEPT7 in primary HSPCs isolated from *Vav1-iCre::Sept7*^flox/flox^ mice (lower panel). Upper panel represents FACS analysis of SEPT7 expression in primary HSPCs isolated from *Vav1-iCre::Sept7*^wt/flox^ mice. R780-A corresponds to APC/Fire 810 anti-mouse CD45 staining, B525-A to SEPT7 (Alexa Fluor 488), and V450-A to DAPI nuclear staining.

To test our hypothesis that SEPT7 is required during early hematopoiesis, we examined its expression in nucleated hematopoietic (CD45-positive) fetal liver cells. *Vav1*-iCre is strongly expressed and active in the fetal liver from embryonic day 9.5 (E9.5) to approximately E11.5 and later, enabling efficient Cre-mediated recombination in HSCs and all downstream hematopoietic lineages (He et al, 2008). At E14.5, we observed a marked reduction, although not a complete loss, of SEPT7 expression in CD45-positive fetal liver cells (Fig 6F). Notably, approximately 20% of hematopoietic (CD45-positive) fetal liver cells remained SEPT7-positive at this stage, suggesting incomplete SEPT7 deletion in a subset of hematopoietic cells during embryonic development (Fig S9A and B). Interestingly, in the bone marrow of adult mice, approximately 46% of lineage-negative HSPCs remained SEPT7-positive, indicating a substantial increase in the proportion of SEPT7-expressing escapees among hematopoietic progenitors during ontogenesis (Figs 6G and S10). Notably, the proportion of SEPT7-positive escapees further increased to 86.5% in the overall bone marrow cell population (Figs 1C and S1C), suggesting progressive enrichment of SEPT7-positive cells during hematopoiesis. This observation supports the existence of selective pressure against SEPT7 deficiency, potentially because of impaired long-term maintenance of SEPT7-deficient progenitors.

## Discussion

**Figure 7.**
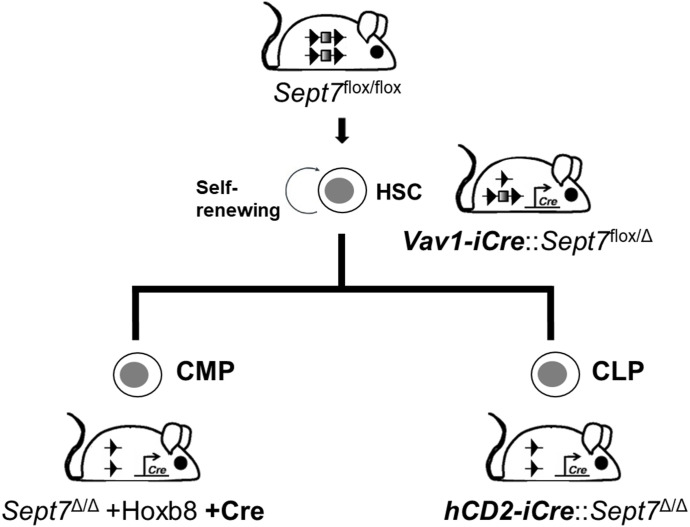
SEPT7 is indispensable at early, but not later stages of hematopoiesis. Schematic representation of the experimental results. Hematopoietic cells escape *Vav1-iCre*-mediated recombination of *Sept7*^flox/flox^ allele, indicating some stable mechanism of protection at early stages of hematopoiesis. *Sept7* is efficiently eliminated at later stages of hematopoiesis, for example, in *hCD2-iCre*::*Sept7*^flox/flox^ T cells or in *Hoxb8*-immortalized *Sept7*^flox/flox^ myeloid progenitors upon recombinant Cre expression.

The role of SEPT7 in cytokinesis during hematopoiesis is not well understood so far (Menon & Gaestel, 2015). Here, we have shown that *Vav1*-driven Cre-mediated recombination is accompanied by a considerable number of *Sept7*^flox/flox^ hematopoietic cells that had not undergone recombination (escapees). These escapees are found in thymocytes, splenocytes, and bone marrow and are detected at all stages of T-cell development, irrespective of higher or lower proliferative rates. In contrast, *Vav1-iCre*::*Sept7*^wt/flox^ mice demonstrated an efficiently deleted floxed allele in HSPCs and all other blood cells analyzed, indicating high efficiency of *Vav1*-driven Cre-mediated recombination. *Vav1-iCre* expresses active Cre in fetal and adult HSCs and all descendants (Siegemund et al, 2015). Incomplete deletion of SEPT7 from hematopoietic cells might indicate impaired repopulation activity of SEPT7-deficient cells and selective advantages of SEPT7-expressing hematopoietic cells. On the other hand, SEPT7 is efficiently eliminated at later stages of hematopoiesis, as observed in T and B cells from *hCD2-iCre*::*Sept7*^flox/flox^ mice, as well as in *Hoxb8*-immortalized hematopoietic progenitors from *Sept7*^flox/flox^ animals after transduction with a Cre-expressing construct. *hCD2-iCre* expresses active Cre at later stages of hematopoiesis if compared with *Vav1-iCre*, namely, in immature and mature B and T lymphocytes (Siegemund et al, 2015). These observations suggest a requirement of SEPT7 for early stages of hematopoiesis and SEPT7 redundancy at later stages of hematopoiesis and in differentiated (mature) blood cells (Fig 7). The aging-associated changes in HSCs and hematopoiesis result from both intrinsic alterations within HSCs and extrinsic influences from the bone marrow (BM) niche (Kamminga & de Haan, 2006; Geiger et al, 2007; Wang et al, 2011; Guidi et al, 2017; Florian et al, 2018; Saçma et al, 2019; Mejia-Ramirez et al, 2020). During early hematopoiesis in the fetal liver, HSCs exhibit high proliferative activity, undergoing more than a 100-fold expansion within ∼5 d during embryogenesis (Morrison et al, 1995; Ema & Nakauchi, 2000). In contrast, in adulthood, HSCs primarily remain in a quiescent state, which restricts self-renewal activity and helps to maintain a largely stable population of functional HSCs (Cheshier et al, 1999; Wilson et al, 2008). The distinct kinetics of HSC self-renewal across different life stages may explain our observations regarding the essential role of SEPT7 in earlier but not later stages of hematopoiesis. Polarity within HSCs is closely linked to their mode of division: young, polarized HSCs predominantly undergo asymmetric division, whereas aged, apolar HSCs tend to divide symmetrically (Florian et al, 2018). Growing evidence indicates the crucial role of septins in establishing molecular asymmetry and cell polarity in fungi and animals (Spiliotis & McMurray, 2020). Thus, in budding yeast, septin barriers are dispensable for cytokinesis (Wloka et al, 2011) but are crucial for maintaining mother–daughter asymmetry, playing a fundamental role in yeast aging (Luedeke et al, 2005; Shcheprova et al, 2008; Gehlen et al, 2011; Khmelinskii et al, 2011; Clay et al, 2014; Denoth-Lippuner et al, 2014). Previous studies have shown that the Cdc42-Borg4-Septin7 axis is essential for maintaining polarity in HSCs and that Septin7-deficient HSCs exhibit reduced engraftment potential along with characteristics of aged HSCs (Kandi et al, 2021). Short-term homing assays using HSCs derived from *Vav1-iCre::Sept7*^flox/flox^ mice demonstrated that homing to the bone marrow is not impaired, indicating that defective homing is unlikely to account for the failure to establish donor chimerism (Kandi et al, 2021). In the current study, we provide evidence that indirectly supports an essential role for SEPT7 in HSC function when demonstrating that SEPT7 is non-essential for in vivo lymphoid development and ex vivo proliferation of *Hoxb8*-immortalized progenitors. We show that SEPT7-expressing escapees arise as early as embryonic day 14.5 and that their proportion among lineage-negative hematopoietic progenitors increases during ontogenesis, indicating strong selection pressure against SEPT7 deficiency in hematopoietic progenitors. The inefficiency of *Vav1-iCre*–mediated *Sept7*^*flox/flox*^ recombination, which could not be overcome by additional Cre expression, suggests that HSCs evade Cre-mediated deletion of *Sept7*. Accordingly, the reduced engraftment of HSCs from *Vav1-iCre::Sept7*^flox/flox^ mice (Kandi et al, 2021) likely reflects exhaustion of repopulating potential during selection against *Sept7* loss.

The data obtained in our current work confirm and expand the previously obtained data on the critical role of septin7 in HSPC function. We demonstrate that septin7 is necessary at early stages of hematopoiesis, at the time when intensive self-renewal of HSPCs is observed. However, at later stages of hematopoiesis, when lymphoid and myeloid precursors are predominantly proliferating, the role of SEPT7 in hematopoietic cell division becomes redundant.

## Materials and Methods

### Experimental animals

*Sept7*^flox/flox^ mice (*Sept7*^tm1Mgl^) targeting the exon 4 of the *Sept7* gene were reported previously (Menon et al, 2014). Lymphocyte-specific *Sept7* knockouts were generated by mating floxed animals with B6-*hCD2-iCre* mice (de Boer et al, 2003; Menon et al, 2014). To obtain a hematopoietic-specific knockout of *Sept7* gene, *Sept7*^flox/flox^ mice were crossed with hematopoietic-specific *Vav1-iCre* transgene-containing mice (Kandi et al, 2021). In animal experiments sex-matched *Sept7*^wt/flox^ and *Sept7*^flox/flox^ littermates were compared. The conditional deletion of *Sept7* was tested by PCR with primer P1: 5′-GGT​ATA​GGG​GAC​TTT​GGG​G-3′, primer P2: 5′-CTT​TGC​ACA​TAT​GAC​TAA​GC-3′, and primer P3: 5′-GCT​TCT​TTT​ATG​TAA​TCC​AGG-3′ as described earlier (Menon et al, 2014). All procedures were in accordance with the German Animal Welfare Legislation, approved by the local Institutional Animal Care and Research Advisory Committee, and covered by the permission of the local veterinary authorities and Lower Saxony State Office for Consumer Protection and Food Safety (reference number 17/2593 and 2024-291).

### Antibodies and reagents

Rabbit anti-human Septin7, C Terminus (Cat# JP18991; IBL; 0.1 mg/ml), Polyclonal rabbit anti-septin 2 (Cat# 11397-1-AP; Acris; 0.18 mg/ml), Polyclonal rabbit anti-Cdc42 (Cat# 2462; Cell Signaling; 0.1 mg/ml), Polyclonal rabbit anti-Septin 6 (sc-20180; Santa Cruz Biotech; 0.1 mg/ml), Polyclonal rabbit anti-septin 9 (Cat# 10769-1-AP; Acris; 0.24 mg/ml), Polyclonal goat anti-EF-2 (C-14) (sc-13004-R; Santa Cruz Biotech; 0.2 mg/ml), Monoclonal mouse anti-GFP (sc-9996; Santa Cruz Biotech; 0.2 mg/ml), Monoclonal mouse anti-GAPDH (Cat# MAB374; Millipore; 1.0 mg/ml), Monoclonal mouse anti-tubulin-α (Cat# T6199; Sigma-Aldrich; 2.0 mg/ml), Monoclonal anti-mouse CD4 (GK1.5) Alexa Fluor 647 (Cat# 100424; BioLegend, RRID:AB_389324; 0.2 mg/ml), Monoclonal anti-mouse CD4 (GK1.5) PerCP-Cy5.5 (Cat# 100434; BioLegend, RRID:AB_893324; 0.2 mg/ml), Monoclonal anti-mouse CD8α (53-6.7) APC (Cat# 100712; BioLegend, RRID:AB_312751; 0.2 mg/ml), Monoclonal anti-mouse CD44 (IM7) PE-Cy7 (Cat# 103030; BioLegend, RRID:AB_830787; 0.2 mg/ml), Monoclonal anti-mouse CD25 (PC61) PerCP-Cy5.5 (Cat# 102030; BioLegend, RRID:AB_893288; 0.2 mg/ml), Monoclonal anti-mouse TCRβ (H57-597) BV421 (Cat# 109230; BioLegend, RRID:AB_2562562; 0.2 mg/ml), Monoclonal anti-mouse CD69 (H1.2F3) PE (Cat# 104508; BioLegend, RRID:AB_313111; 0.2 mg/ml), APC/Fire 810 anti-mouse CD45 antibody (Clone 30-F11; Catalog No. 103173; BioLegend; 0.2 mg/ml), Donkey anti-rabbit IgG Alexa Fluor 488 (A-21206; Invitrogen; 2.0 mg/ml), Goat anti-mouse IgG Alexa Fluor 680 (A21057; Invitrogen; 2.0 mg/ml), HRP-labeled goat anti-mouse IgG (Cat# 115-035-003; Dianova; 0.8 mg/ml), HRP-labeled goat anti-rabbit IgG (Cat# 111-035-003; Dianova; 0.8 mg/ml), HRP-labeled mouse anti-goat IgG (sc-2354; Santa Cruz Biotech; 0.4 mg/ml), DAPI (Cat# 6335.1; Carl Roth), Polybrene (Cat# H9268; Sigma-Aldrich), Doxycycline (Cat# D9891; Sigma-Aldrich), β-Estradiol (Cat# E8875; Sigma-Aldrich), mIL-3 (Cat# 213-13; Peprotech), mIL-11 (Cat# 220-11; Peprotech), mSCF (Cat# 250-03; Peprotech), and mFlt-3L (Cat# 250-31L; Peprotech). For Western blot analysis, primary antibodies were used at a dilution of 1:1,000, and secondary antibodies were used at a dilution of 1:2,000. For immunostaining, primary antibodies were used at a dilution of 1:200, and secondary antibodies were used at a dilution of 1:500. For FACS analysis, primary antibodies were used at a dilution of 1:100, and secondary antibodies were used at a dilution of 1:500.

### Cloning of gammaretroviral dox-inducible GFP-*Sept7*

For cloning of the gammaretroviral pSERS11-T11-GFP-*Sept7*-PGK-M2 construct, which harbor a GFP-fused *Sept7* downstream of a doxycycline-inducible promoter, we used mouse *Sept7* transcript variant 2 (NM_001205367.1) cDNA.

### Gammaretroviral virus production

The plasmids pSERS11-T11-GFP-*Sept7*-PGK-M2 (coding dox-inducible GFP-Sept7) or MSCV-ERHBD-*Hoxb8* (Redecke et al, 2013) were co-transfected together with the ecotropic packaging vector pCL-Eco (Imgenex) into BD EcoPack 2-293 packaging cell line using polyethylenimine. Eighteen hours after transfection, the supernatant was replaced by fresh growth medium (DMEM [Gibco]), supplemented with 10% (vol/vol) FBS (Capricorn) and antibiotics penicillin G (100 IU/ml) and streptomycin sulfate (100 IU/ml). Virus-containing supernatant was collected for 3–4 d every 24 h and pooled together. Supernatant was filtered through a 0.45 μm filter (Sarstedt) and concentrated by ultracentrifugation in SW40-Ti rotor from Beckman Coulter: 20,000 rpm, 90 min, 4°C. Pellet was resuspended in 2 ml IMDM (12440-053; Gibco) supplemented with 15% FBS and antibiotics (P/S) and was filtered through 0.45 μm filter.

### pLenti-Cre virus production

The following plasmids: pLBid.pA.CTE.nlsCre.minCMV.SF.mCherry.PRE, pcDNA3.NovB2p (Sullivan & Ganem, 2005), pRSV.Rev (kindly provided by Thomas J. Hope, Northwestern University, Chicago, IL), LentiGag/Pol (Schambach et al, 2006), and vesicular stomatitis virus glycoprotein G (Yang et al, 1995) (5 μg each) were co-transfected into a 10-cm plate with 90% confluent HEK293 cells as described elsewhere (Maetzig et al, 2010). Eighteen hours after transfection, the supernatant was replaced by fresh growth medium (DMEM [Gibco]), supplemented with 10% (vol/vol) FBS (Capricorn) and antibiotics penicillin G (100 IU/ml) and streptomycin sulfate (100 IU/ml). Virus-containing supernatant was collected for 3–4 d every 24 h and pooled together. Supernatant was filtered through 0.45 μm filter (Sarstedt) and concentrated by ultracentrifugation in SW40-Ti Beckman: 25,000 rpm, 120 min, 4°C. Pellet was resolved in 2 ml IMDM (12440-053; Gibco) supplemented with 15% FBS and antibiotics (P/S) and was filtered through 0.45 μm filter. In some experiments commercially available pre-made lentiviral particles expressing nuclear-permeant Cre recombinase under the CMV early enhancer/chicken β-actin promoter and RFP-Blasticidin fusion dual marker under RSV promoter, which allows the selection of the positive transduced stable cells for long-term culture via RFP cell sorting or via antibiotic selection (LVP577-PBS Gentarget).

### Generation and cell culture of *Hoxb8* progenitor cell line

Bone marrow lineage-negative cells were isolated by the MagCellect Mouse Hematopoietic Cell Lineage Depletion Kit (R&D: # MAGM209). Cells were cultured in StemSpan Serum-Free Expansion Medium (Cat. 09600; STEMCELL) supplemented with P/S; 20 ng/ml IL-3; 100 ng/ml IL-11; 50 ng/ml Stem Cell Factor (SCF) and 100 ng/ml Flt-3L for 2 d before virus transduction. Cells were transduced on the third day: 1 ml *Hoxb8* virus supplemented with 8 μg/ml Polybrene was added to 100 μl of cell suspension in 12-well plate followed by centrifugation at 1013 x g, 30 min, 32°C. After centrifugation, 20 ng/ml IL-3; 100 ng/ml IL-11; 100 ng/ml SCF; 100 ng/ml Flt-3L and 1 μM β-Estradiol were added to the cells. At 24 h after transduction virus was replaced with growth medium: IMDM (12440-053; Gibco) supplemented with 15% FBS; P/S; 20 ng/ml IL-3; 100 ng/ml IL-11; 100 ng/ml SCF; 100 ng/ml Flt-3L und 1 μM β-Estradiol. Selection of transduced cells with 0.5–1 mg/ml Geneticin (Gibco) was started 72 h after transduction. Cells were dispensed every 3–4 d in fresh medium and transferred into new wells. Once the cell populations were stably expanding, cells were kept at concentrations between 1 × 10^4^ cells/ml medium and 1 × 10^6^ cells/ml medium.

### In vitro T cell activation assay

T cells were isolated from the spleen of 5-mo-old mice using the EasySep Mouse T Cell Isolation Kit (Catalog # 19851; STEMCELL Technologies). Isolated T cells were labeled with 10 μM CFSE (carboxyfluorescein diacetate succinimidyl ester; Catalog # 65-0850-84; eBioscience) for 20 min at 37°C and cultured in 24-well plates at a density of 1.3 × 10^6^ cells/ml in complete Roswell Park Memorial Institute medium supplemented with 10% FCS, P/S, 50 ng/ml IL-2 (=250 U/ml [Cat. 212-12; PeproTech]), and 50 μM ß-Me. T cells were stimulated with 25 µl of Dynabeads Mouse T-Activator CD3/CD28 (Catalog # 11456D; Gibco) per sample and harvested at the time indicated.

### Western immunoblotting

Protein extracts were prepared by direct lysis of the cells with 2× Laemmli SDS sample buffer. Protein lysates were separated by SDS-PAGE on 7.5–16% gradient gels and transferred by wet blotting to Hybond ECL nitrocellulose membranes (GE Healthcare). Western blots were blocked with 5% powdered skim milk in PBS with 0.1% Tween 20 for 1 h at room temperature, followed by overnight incubation with the primary antibody at 4 °C. After intensive washes with PBS containing 0.1% Tween 20, membranes were incubated for 1 h with horseradish peroxidase-conjugated secondary antibodies at room temperature. Digital chemiluminescence images were taken by a Luminescent Image Analyzer LAS-3000 (Fujifilm).

### Intracellular immunofluorescence staining

Glass coverslips were treated with 1% Alcian blue solution for 20 min in a 12-well plate and washed with PBS. Suspension cells were added in PBS, and the plate was centrifuged at 1013 x g for 10 min at 4°C. Cells were fixed in 1 ml 4% PFA/5% Methanol/PBS for 15 min, washed with PBS, and permeabilized with 0.25% Triton X-100–PBS for 30 min at room temperature. Blocking was performed using 4% bovine serum albumin (BSA) for 1 h at 4°C. Primary antibodies were used at a 1:200 dilution in 1% BSA–PBS for 1 h at room temperature. Secondary antibodies or Alexa Fluor 647-conjugated phalloidin was used at a 1:500 dilution in 1% BSA–PBS. Imaging was performed using Cytation1 (Agilent BioTek) with standard settings. Objective, 20× Olympus Plan Fluorite (20×/0.45 NA), and objective, 4× Olympus Plan Fluorite (4×/0.13 NA). GFP filter cube (EX 469/35 nm, EM 525/39 nm), DAPI filter cube (EX 377/50 nm, EM 447/60 nm), RFP filter cube (EX 531/40 nm, EM 593/40 nm), and CY5.5 filter cube (EX 647/57 nm, EM 794/160 nm).

### Flow cytometry analysis

Thymocyte single cell suspension were generated by mashing thymi through a 100 μm sterile nylon cell strainer (Corning). The cell suspension was further filtered through a 30 μm filter, to ensure maximum removal of tissue debris. Immature thymocyte populations were characterized by staining for CD4 (Alexa Fluor 647), CD8α (APC), CD44 (PE-Cy7), CD25 (PerCP-Cy5.5) surface markers, followed by intracellular staining with TCRβ (BV421) and unconjugated anti-human Septin7 (see below). Mature thymocytes were characterized by staining for CD4 (PerCP-Cy5.5), CD8α (APC), CD69 (PE), TCRβ (BV421) followed by intracellular staining with unconjugated anti-human Septin7. For SEPT7 staining, cells were fixed by adding three volumes of 4% PFA and incubating for 20 min at room temperature. Cells were subsequently washed with PBS and resuspended in FACS buffer (3% FCS in PBS). Where indicated, surface staining was performed using APC/Fire 810 anti-mouse CD45 antibody (1:100 dilution) for 15 min at 4°C in the dark. After one wash with FACS buffer, cells were permeabilized in permeabilization/saponin buffer (3% FCS and 0.25 % saponin in PBS) for 15 min at room temperature in the dark. After permeabilization, cells were incubated with primary antibodies diluted 1:100 in saponin buffer for 30 min at room temperature in the dark. Cells were then washed once with saponin buffer and incubated with secondary antibodies (anti-rabbit Alexa Fluor 488 diluted 1:500 in saponin buffer) for an additional 30 min in the dark. After a further wash with saponin buffer, cells were incubated in FACS buffer supplemented with 0.25 μg/ml DAPI for 5 min, filtered through nylon 75 μm mesh and subsequently analyzed by flow cytometry using a Beckman Coulter CytoFLEX. All centrifugation steps were performed at 300 *g* for 5 min at 4°C. Data are presented as total counts (count) or as percentage of maximum (% Max), where the highest peak (mode) of each sample is set to 100%, allowing easier visual comparison of shapes between samples when total cell counts differ.

### Peripheral blood analysis

Mice were sacrificed by CO2 Inhalation and subsequent exsanguination via cardiac puncture. The hereby collected blood samples were sampled in tubes covered with ethylenediaminetetraacetic acid (Microtube 1.3 ml, Sarstedt) and subjected to differential blood count and analysis with Element HT5 Veterinary Hematology Analyzer (Scil animal care company, Antech Diagnostics Germany GmbH).

### Far red cell proliferation

The CellTrace Far Red kit (Cat. No. C34564; Thermo Fisher Scientific) was used to monitor cell proliferation by dye dilution. 100,000 cells were washed with PBS and were resuspended in 200 μl of 1 μM FarRed solution in PBS followed by incubation for 20 min at 37°C. Reaction was stopped by addition of 1 ml of complete medium. After 5 min incubation at room temperature, the cells were resuspended in 650 μl of growth medium and distributed 200 μl (30,000 cells) pro well in 96-well well plate. Cells were analyzed by flow cytometer with 638 nm excitation and a 780 nm emission filter using BeckmanCoulter Cytoflex. A mean Far Red peak number was quantified using Kaluza GraphPad.

### Population doubling time

2 × 10^4^ cells were repeatedly seeded in 1 ml growth medium every 3–4 d, collected and counted. Population doubling time was estimated after formula: Doubling Time = (duration * log(2))/(log(final concentration) − log(initial concentration)), where “log” is the logarithm to base n.

## Supplementary Material

Reviewer comments

## Data Availability

All data supporting the findings of this study are available within the paper and its Supplementary Information. Any additional information required is available from the lead contact upon request. This paper reports no original code.

## References

[bib1] Addi C, Bai J, Echard A (2018) Actin, microtubule, septin and ESCRT filament remodeling during late steps of cytokinesis. Curr Opin Cell Biol 50: 27–34. 10.1016/j.ceb.2018.01.007 29438904

[bib2] Cheshier SH, Morrison SJ, Liao X, Weissman IL (1999) In vivo proliferation and cell cycle kinetics of long-term self-renewing hematopoietic stem cells. Proc Natl Acad Sci U S A 96: 3120–3125. 10.1073/pnas.96.6.3120 10077647 PMC15905

[bib3] Clay L, Caudron F, Denoth-Lippuner A, Boettcher B, Buvelot Frei S, Snapp EL, Barral Y (2014) A sphingolipid-dependent diffusion barrier confines ER stress to the yeast mother cell. Elife 3: e01883. 10.7554/eLife.01883 24843009 PMC4009826

[bib4] de Boer J, Williams A, Skavdis G, Harker N, Coles M, Tolaini M, Norton T, Williams K, Roderick K, Potocnik AJ, (2003) Transgenic mice with hematopoietic and lymphoid specific expression of Cre. Eur J Immunol 33: 314–325. 10.1002/immu.200310005 12548562

[bib5] Denoth-Lippuner A, Krzyzanowski MK, Stober C, Barral Y (2014) Role of SAGA in the asymmetric segregation of DNA circles during yeast ageing. Elife 3: e03790. 10.7554/elife.03790 25402830 PMC4232608

[bib6] Ema H, Nakauchi H (2000) Expansion of hematopoietic stem cells in the developing liver of a mouse embryo. Blood 95: 2284–2288. 10.1182/blood.v95.7.2284.007k14_2284_2288 10733497

[bib7] Fededa JP, Gerlich DW (2012) Molecular control of animal cell cytokinesis. Nat Cell Biol 14: 440–447. 10.1038/ncb2482 22552143

[bib8] Florian MC, Klose M, Sacma M, Jablanovic J, Knudson L, Nattamai KJ, Marka G, Vollmer A, Soller K, Sakk V, (2018) Aging alters the epigenetic asymmetry of HSC division. PLoS Biol 16: e2003389. 10.1371/journal.pbio.2003389 30235201 PMC6168157

[bib9] Gehlen LR, Nagai S, Shimada K, Meister P, Taddei A, Gasser SM (2011) Nuclear geometry and rapid mitosis ensure asymmetric episome segregation in yeast. Curr Biol 21: 25–33. 10.1016/j.cub.2010.12.016 21194950

[bib10] Geiger H, Koehler A, Gunzer M (2007) Stem cells, aging, niche, adhesion and Cdc42: A model for changes in cell-cell interactions and hematopoietic stem cell aging. Cell Cycle 6: 884–887. 10.4161/cc.6.8.4131 17404508

[bib11] Green RA, Paluch E, Oegema K (2012) Cytokinesis in animal cells. Annu Rev Cell Dev Biol 28: 29–58. 10.1146/annurev-cellbio-101011-155718 22804577

[bib12] Guidi N, Sacma M, Ständker L, Soller K, Marka G, Eiwen K, Weiss JM, Kirchhoff F, Weil T, Cancelas JA, (2017) Osteopontin attenuates aging-associated phenotypes of hematopoietic stem cells. EMBO J 36: 840–853. 10.15252/embj.201694969 28254837 PMC5376966

[bib13] Hartwell LH, Culotti J, Reid B (1970) Genetic control of the cell-division cycle in yeast. I. Detection of mutants. Proc Natl Acad Sci U S A 66: 352–359. 10.1073/pnas.66.2.352 5271168 PMC283051

[bib14] He Y, Ecker JR (2015) Non-CG methylation in the human genome. Annu Rev Genomics Hum Genet 16: 55–77. 10.1146/annurev-genom-090413-025437 26077819 PMC4729449

[bib15] He C, Hu H, Braren R, Fong SY, Trumpp A, Carlson TR, Wang RA (2008) c-myc in the hematopoietic lineage is crucial for its angiogenic function in the mouse embryo. Development 135: 2467–2477. 10.1242/dev.020131 18550710 PMC2597486

[bib16] Kamminga LM, de Haan G (2006) Cellular memory and hematopoietic stem cell aging. Stem Cells 24: 1143–1149. 10.1634/stemcells.2005-0345 16456126

[bib17] Kandi R, Senger K, Grigoryan A, Soller K, Sakk V, Schuster T, Eiwen K, Menon MB, Gaestel M, Zheng Y, (2021) Cdc42-Borg4-Septin7 axis regulates HSC polarity and function. EMBO Rep 22: e52931. 10.15252/embr.202152931 34661963 PMC8647144

[bib18] Karasmanis EP, Hwang D, Nakos K, Bowen JR, Angelis D, Spiliotis ET (2019) A septin double ring controls the spatiotemporal organization of the ESCRT machinery in cytokinetic abscission. Curr Biol 29: 2174–2182.e7. 10.1016/j.cub.2019.05.050 31204162 PMC6620605

[bib19] Khmelinskii A, Meurer M, Knop M, Schiebel E (2011) Artificial tethering to nuclear pores promotes partitioning of extrachromosomal DNA during yeast asymmetric cell division. Curr Biol 21: R17–R18. 10.1016/j.cub.2010.11.034 21215928

[bib20] Kremer BE, Haystead T, Macara IG (2005) Mammalian septins regulate microtubule stability through interaction with the microtubule-binding protein MAP4. Mol Biol Cell 16: 4648–4659. 10.1091/mbc.E05-03-0267 16093351 PMC1237071

[bib21] Liu R, Long Q, Zou X, Wang Y, Pei Y (2021) DNA methylation occurring in Cre-expressing cells inhibits loxP recombination and silences loxP-sandwiched genes. New Phytol 231: 210–224. 10.1111/nph.17353 33742463

[bib22] Luedeke C, Frei SB, Sbalzarini I, Schwarz H, Spang A, Barral Y (2005) Septin-dependent compartmentalization of the endoplasmic reticulum during yeast polarized growth. J Cell Biol 169: 897–908. 10.1083/jcb.200412143 15967812 PMC2171641

[bib23] Maetzig T, Galla M, Brugman MH, Loew R, Baum C, Schambach A (2010) Mechanisms controlling titer and expression of bidirectional lentiviral and gammaretroviral vectors. Gene Ther 17: 400–411. 10.1038/gt.2009.129 19847204

[bib24] Marquardt J, Chen X, Bi E (2021) Septin assembly and remodeling at the cell division site during the cell cycle. Front Cell Dev Biol 9: 793920. 10.3389/fcell.2021.793920 34901034 PMC8656427

[bib25] Mejia-Ramirez E, Geiger H, Florian MC (2020) Loss of epigenetic polarity is a hallmark of hematopoietic stem cell aging. Hum Mol Genet 29: R248–R254. 10.1093/HMG/DDAA189 32821941

[bib26] Menon MB, Gaestel M (2015) Sep(t)arate or not - how some cells take septin-independent routes through cytokinesis. J Cell Sci 128: 1877–1886. 10.1242/jcs.164830 25690008

[bib27] Menon MB, Sawada A, Chaturvedi A, Mishra P, Schuster-Gossler K, Galla M, Schambach A, Gossler A, Förster R, Heuser M, (2014) Genetic deletion of SEPT7 reveals a cell type-specific role of septins in microtubule destabilization for the completion of cytokinesis. PLoS Genet 10: e1004558. 10.1371/journal.pgen.1004558 25122120 PMC4133155

[bib28] Menon MB, Yakovleva T, Ronkina N, Suwandi A, Odak I, Dhamija S, Sandrock I, Hansmann F, Baumgärtner W, Förster R, (2022) *Lyz2*-Cre-mediated genetic deletion of *Septin7* reveals a role of septins in macrophage cytokinesis and *Kras*-driven tumorigenesis. Front Cell Dev Biol 9: 795798. 10.3389/fcell.2021.795798 35071236 PMC8772882

[bib29] Morrison SJ, Hemmati HD, Wandycz AM, Weissman IL (1995) The purification and characterization of fetal liver hematopoietic stem cells. Proc Natl Acad Sci U S A 92: 10302–10306. 10.1073/pnas.92.22.10302 7479772 PMC40784

[bib30] Mujal AM, Gilden JK, Gérard A, Kinoshita M, Krummel MF (2016) A septin requirement differentiates autonomous and contact-facilitated T cell proliferation. Nat Immunol 17: 315–322. 10.1038/ni.3330 26692174 PMC4755847

[bib31] Redecke V, Wu R, Zhou J, Finkelstein D, Chaturvedi V, High AA, Häcker H (2013) Hematopoietic progenitor cell lines with myeloid and lymphoid potential. Nat Methods 10: 795–803. 10.1038/nmeth.2510 23749299 PMC4131762

[bib32] Saçma M, Pospiech J, Bogeska R, de Back W, Mallm JP, Sakk V, Soller K, Marka G, Vollmer A, Karns R, (2019) Haematopoietic stem cells in perisinusoidal niches are protected from ageing. Nat Cell Biol 21: 1309–1320. 10.1038/s41556-019-0418-y 31685996

[bib33] Schambach A, Bohne J, Chandra S, Will E, Margison GP, Williams DA, Baum C (2006) Equal potency of gammaretroviral and lentiviral SIN vectors for expression of O6-methylguanine-DNA methyltransferase in hematopoietic cells. Mol Ther 13: 391–400. 10.1016/j.ymthe.2005.08.012 16226060

[bib34] Shcheprova Z, Baldi S, Frei SB, Gonnet G, Barral Y (2008) A mechanism for asymmetric segregation of age during yeast budding. Nature 454: 728–734. 10.1038/nature07212 18660802

[bib35] Siegemund S, Shepherd J, Xiao C, Sauer K (2015) *hCD2-iCre* and *Vav-iCre* mediated gene recombination patterns in murine hematopoietic cells. PLoS One 10: e0124661. 10.1371/journal.pone.0124661 25884630 PMC4401753

[bib36] Spiliotis ET, McMurray MA (2020) Masters of asymmetry - lessons and perspectives from 50 years of septins. Mol Biol Cell 31: 2289–2297. 10.1091/mbc.E19-11-0648 32991244 PMC7851956

[bib37] Sullivan CS, Ganem D (2005) A virus-encoded inhibitor that blocks RNA interference in mammalian cells. J Virol 79: 7371–7379. 10.1128/jvi.79.12.7371-7379.2005 15919892 PMC1143619

[bib38] Wang J, Geiger H, Rudolph KL (2011) Immunoaging induced by hematopoietic stem cell aging. Curr Opin Immunol 23: 532–536. 10.1016/j.coi.2011.05.004 21872769

[bib39] Wilson A, Laurenti E, Oser G, van der Wath RC, Blanco-Bose W, Jaworski M, Offner S, Dunant CF, Eshkind L, Bockamp E, (2008) Hematopoietic stem cells reversibly switch from dormancy to self-renewal during homeostasis and repair. Cell 135: 1118–1129. 10.1016/j.cell.2008.10.048 19062086

[bib40] Wloka C, Nishihama R, Onishi M, Oh Y, Hanna J, Pringle JR, Krauss M, Bi E (2011) Evidence that a septin diffusion barrier is dispensable for cytokinesis in budding yeast. Biol Chem 392: 813–829. 10.1515/BC.2011.083 21824009

[bib41] Yang Y, Vanin EF, Whitt MA, Fornerod M, Zwart R, Schneiderman RD, Grosveld G, Nienhuis AW (1995) Inducible, high-level production of infectious murine leukemia retroviral vector particles pseudotyped with vesicular stomatitis virus G envelope protein. Hum Gene Ther 6: 1203–1213. 10.1089/hum.1995.6.9-1203 8527479

