# Supplementary material for: Septin7 is essential in early hematopoiesis, but redundant at later stages
Source: Life Sci Alliance. 2026 Jul 13;9(10):e202603637. doi: 10.26508/lsa.202603637 (PMC13365248; doi:10.26508/lsa.202603637)
Supplement: Supplementary file 1 [file LSA_2026_03637_SdataF1_F2_F3_F4_F5_F6.pdf]

Full-size images of the gels/blots shown in Figures 1 to 6

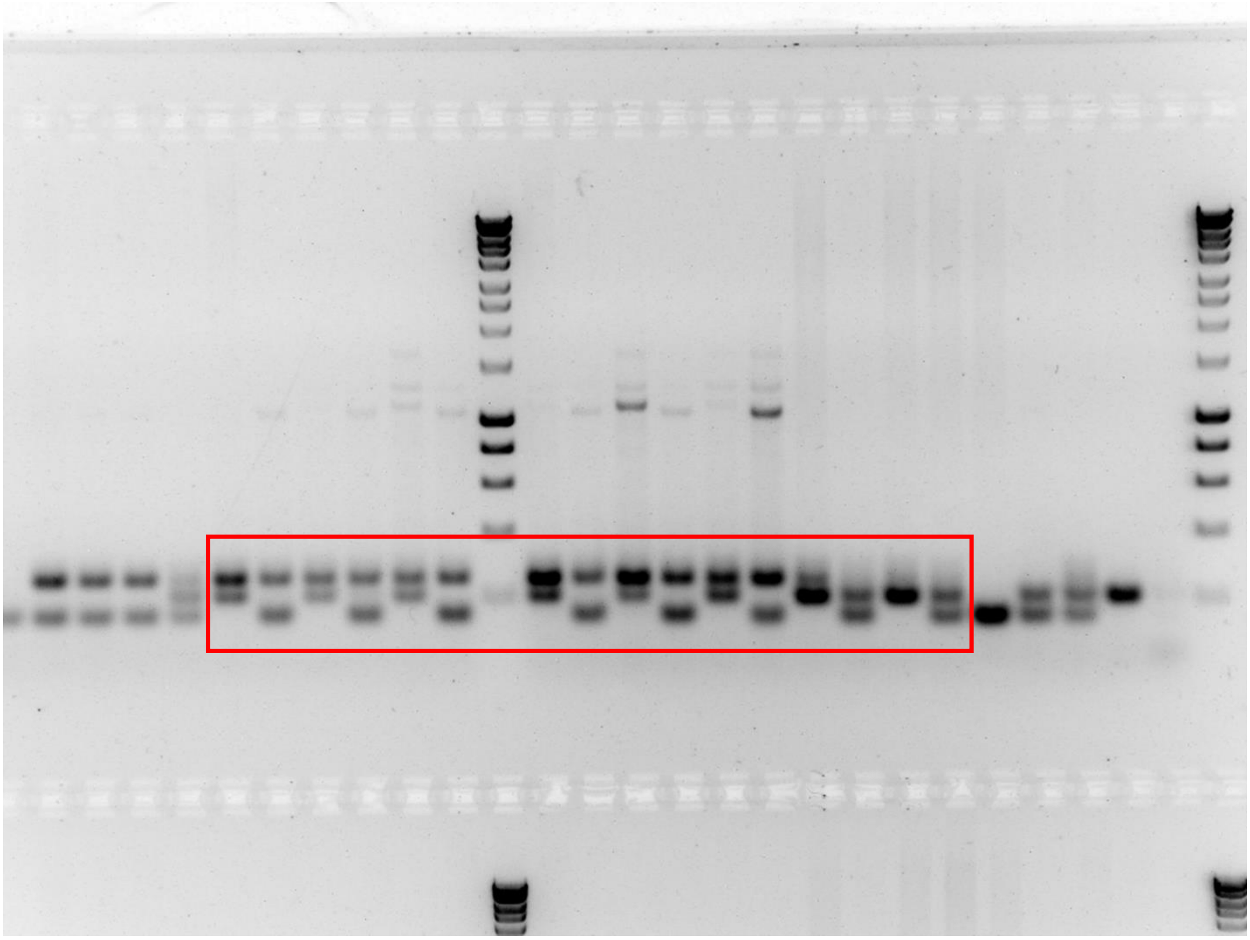

Full-size image of the agarose gel shown in Fig.1a

SEPT7

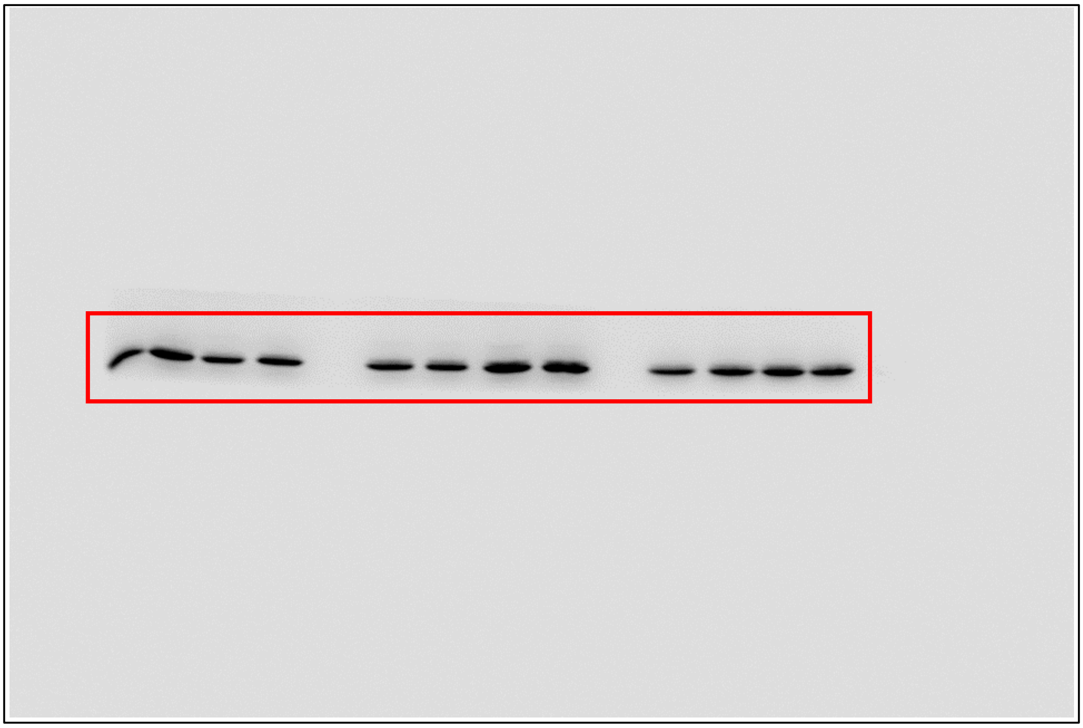

EF2

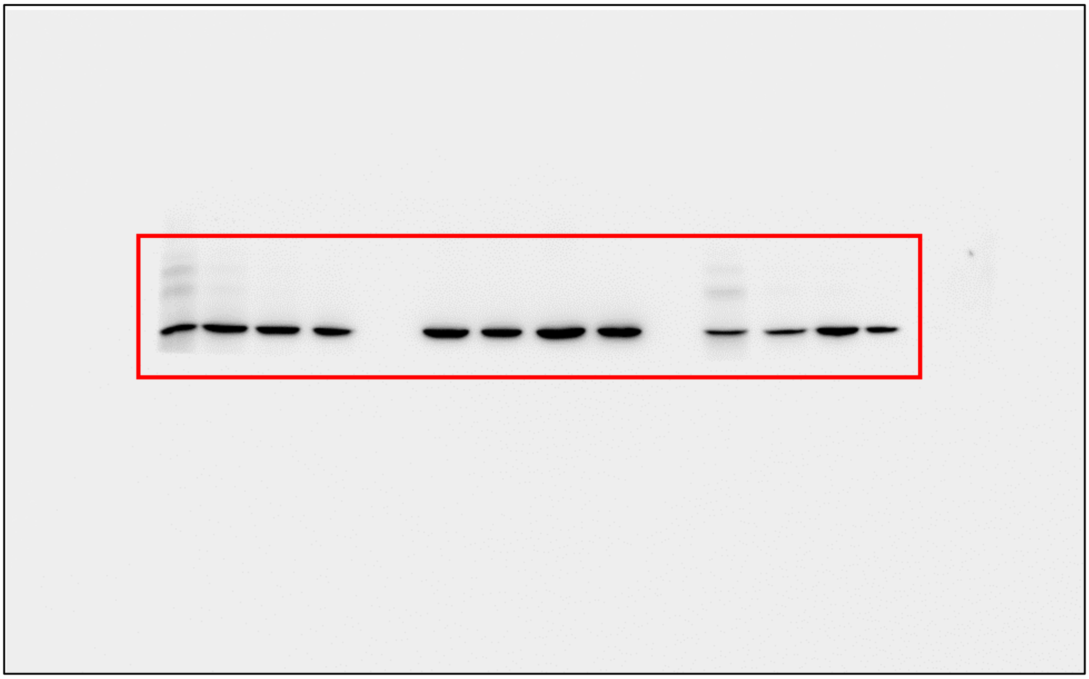

Full-size images of the blots shown in Fig.1b

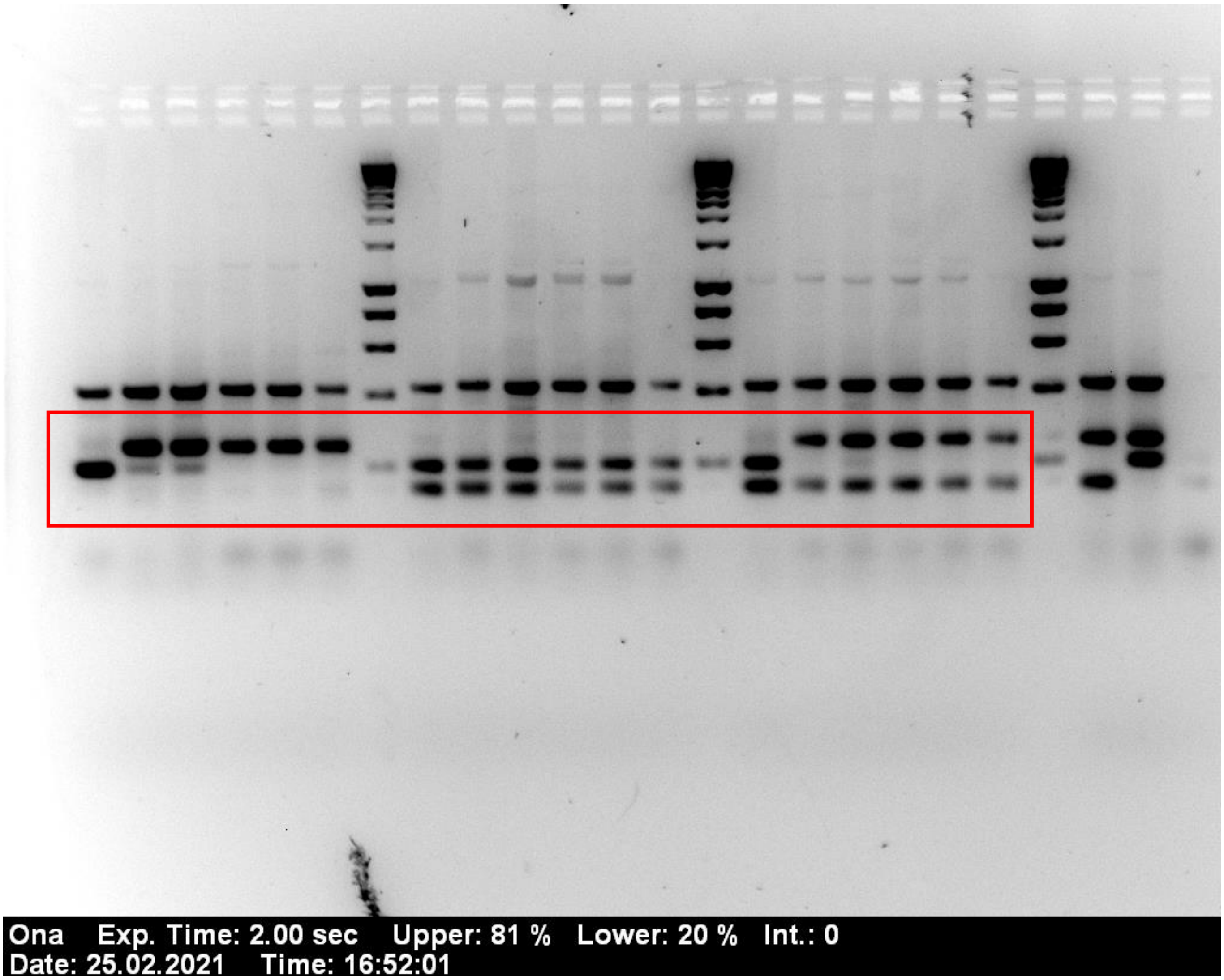

Full-size image of the agarose gel shown in Fig.2a

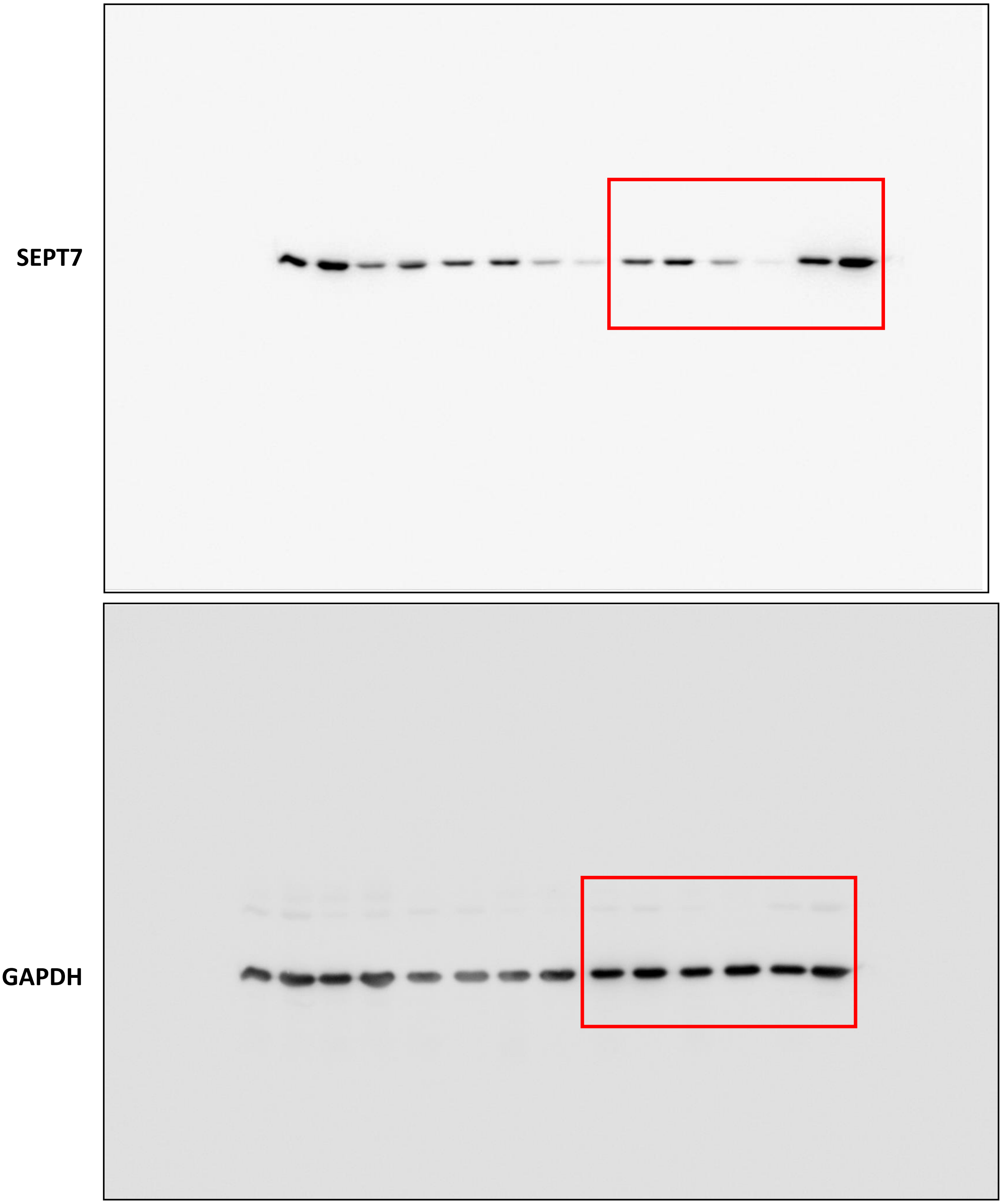

Full-size images of the blots shown in Fig.2b

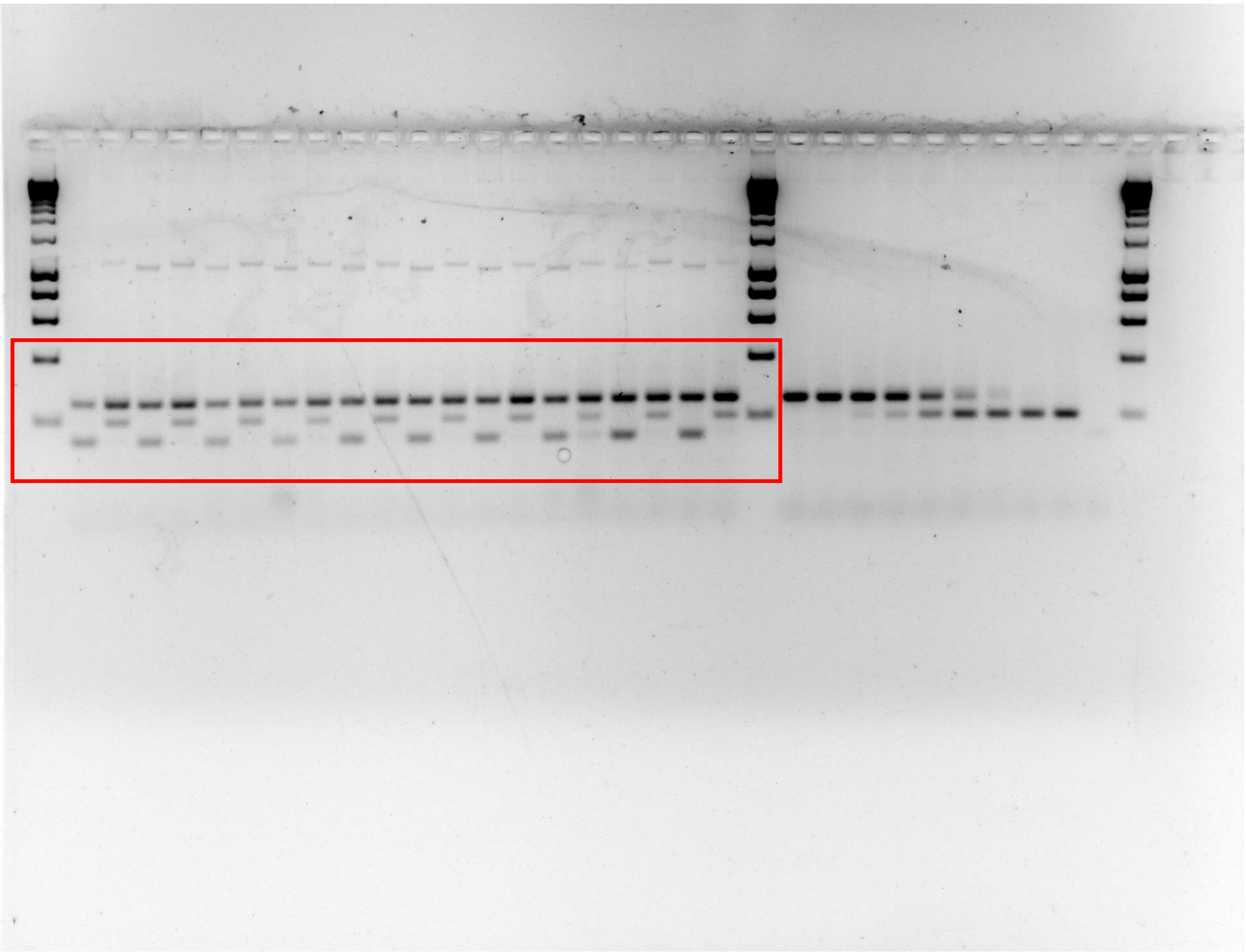

Full-size image of the agarose gel shown in Fig.3a

SEPT7

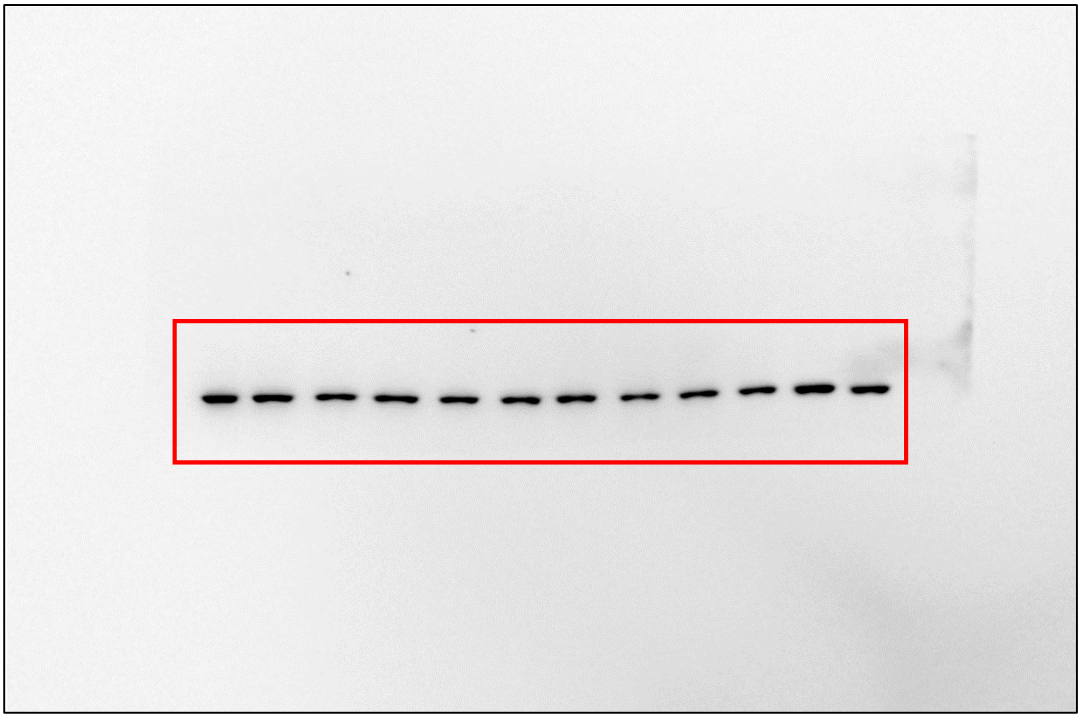

GAPDH

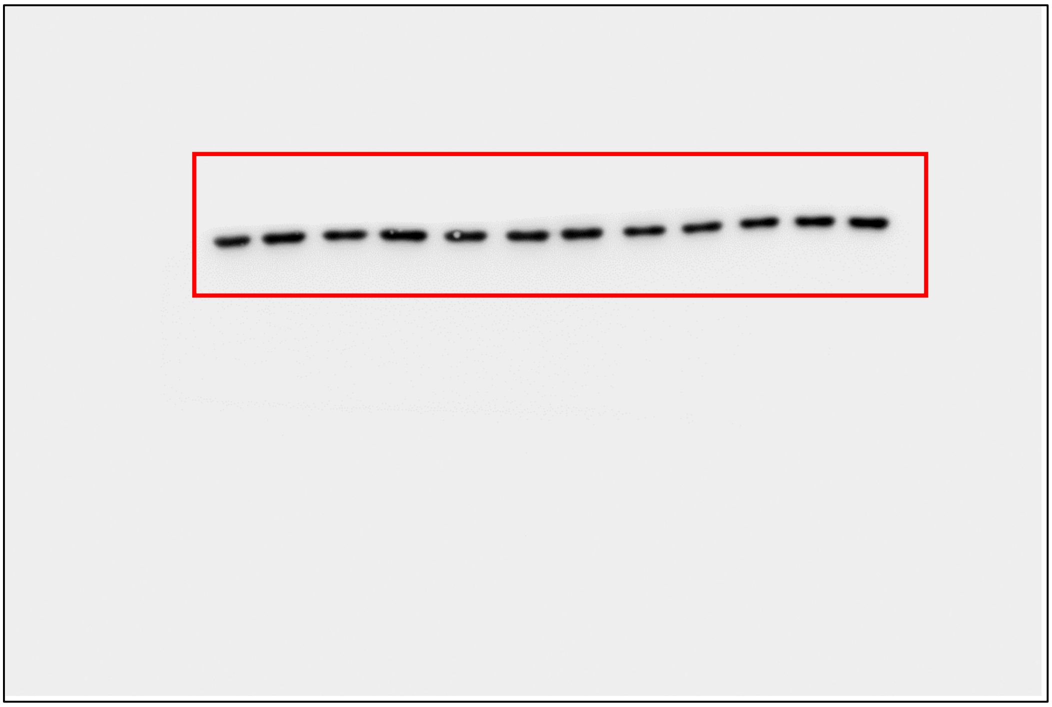

Full-size images of the blots shown in Fig.3b

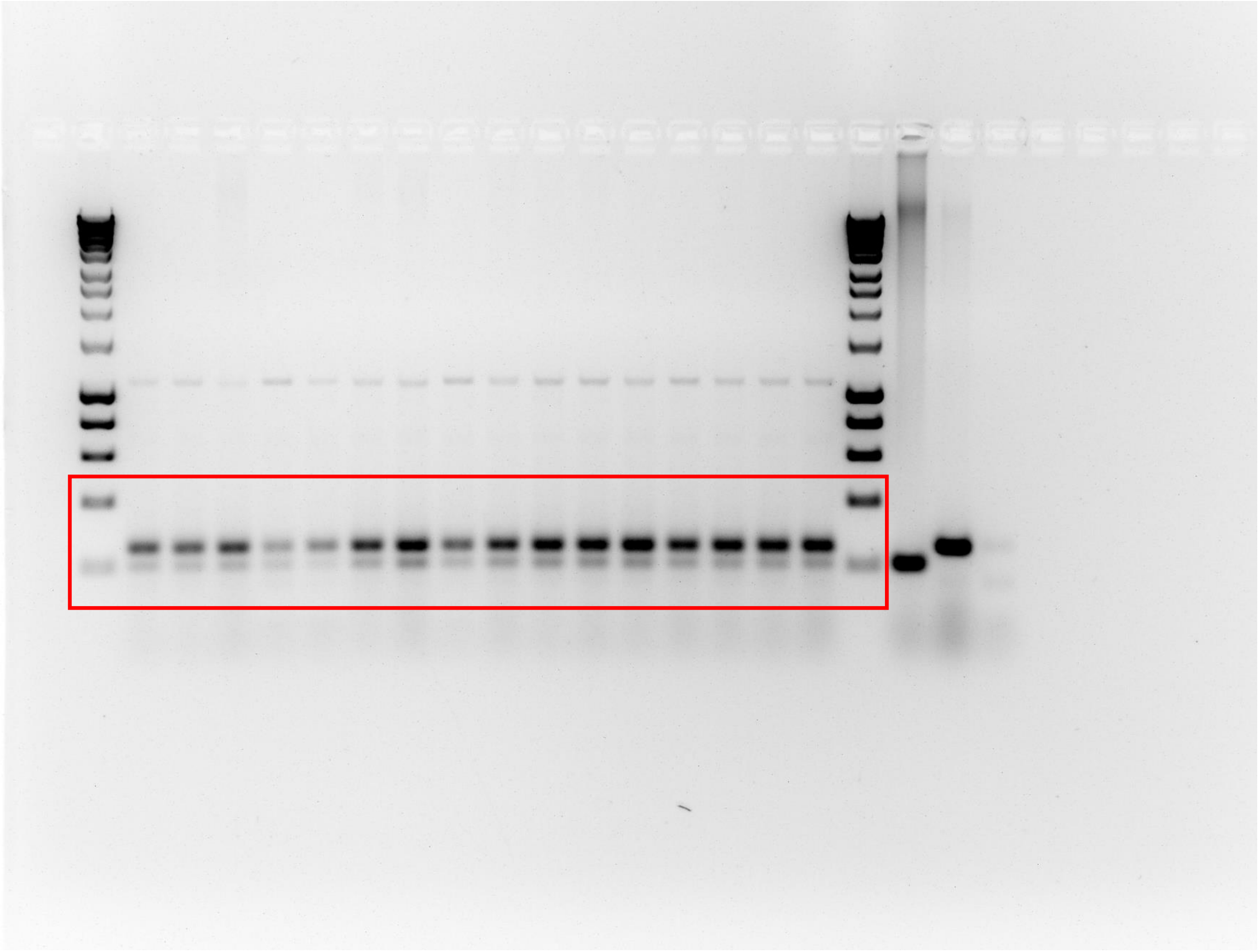

Full-size image of the agarose gel shown in Fig.3e

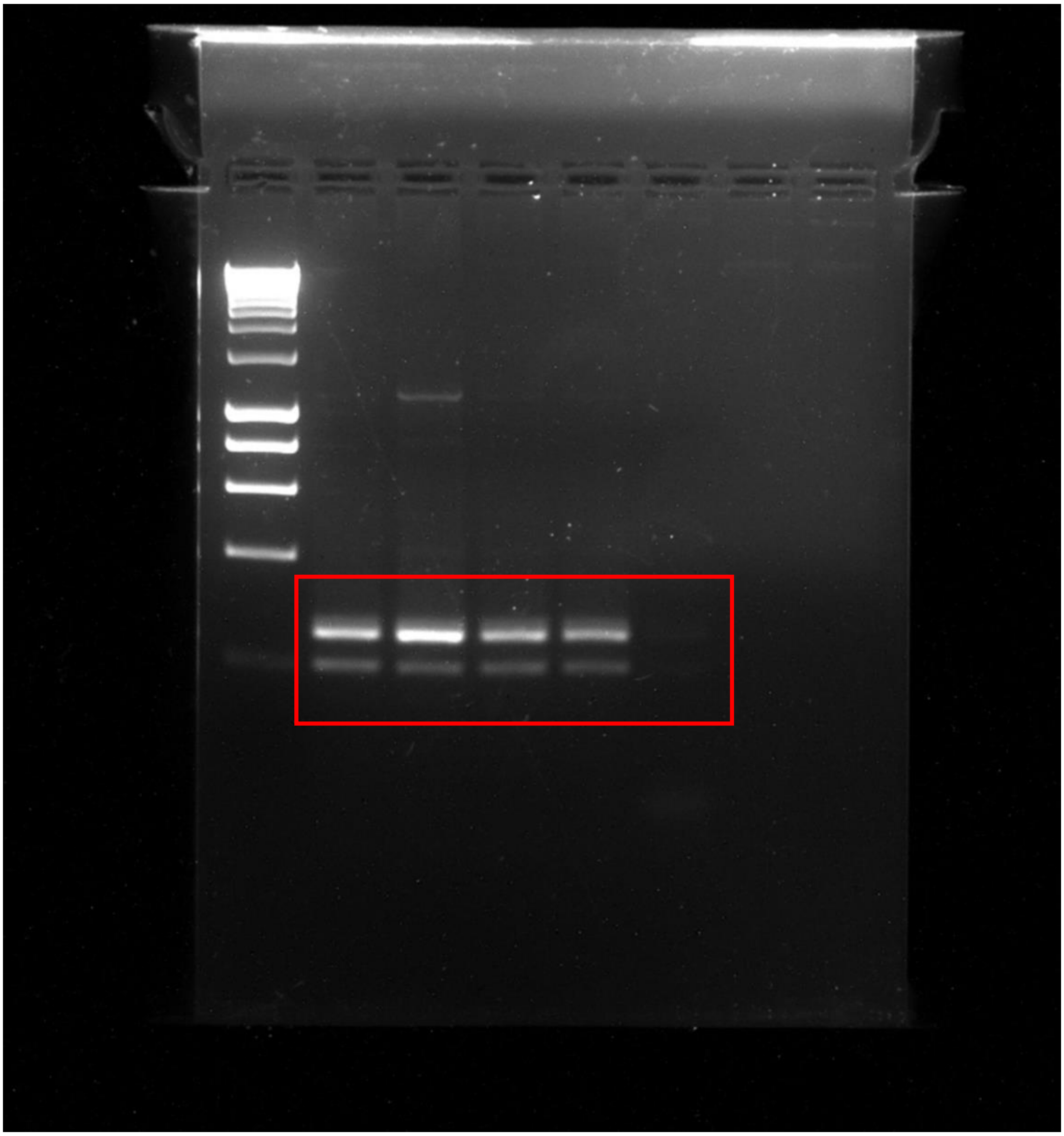

Full-size image of the agarose gel shown in Fig.4c

SEPT7

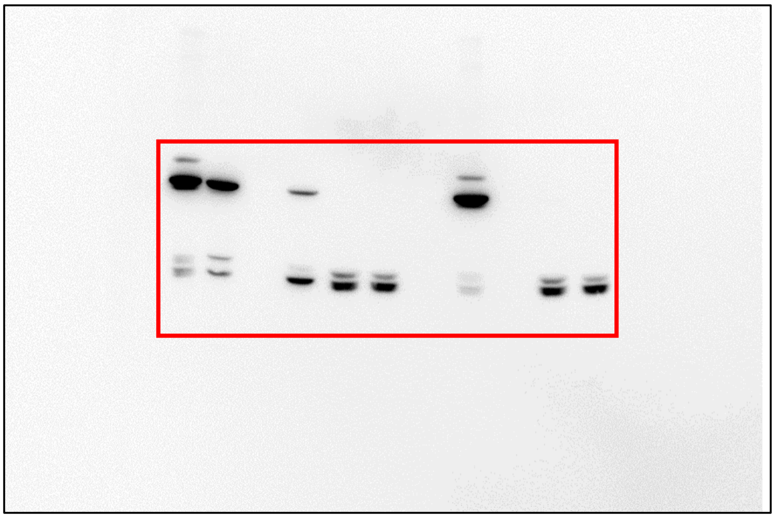

SEPT2

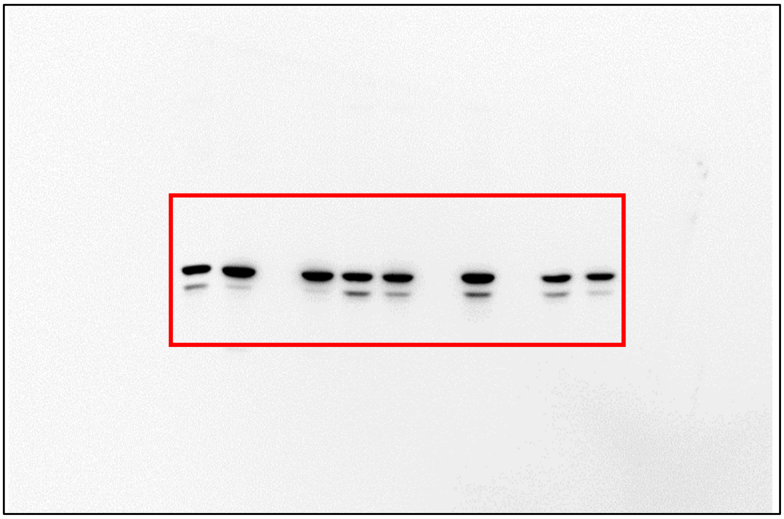

Full-size images of the blots shown in Fig.4d

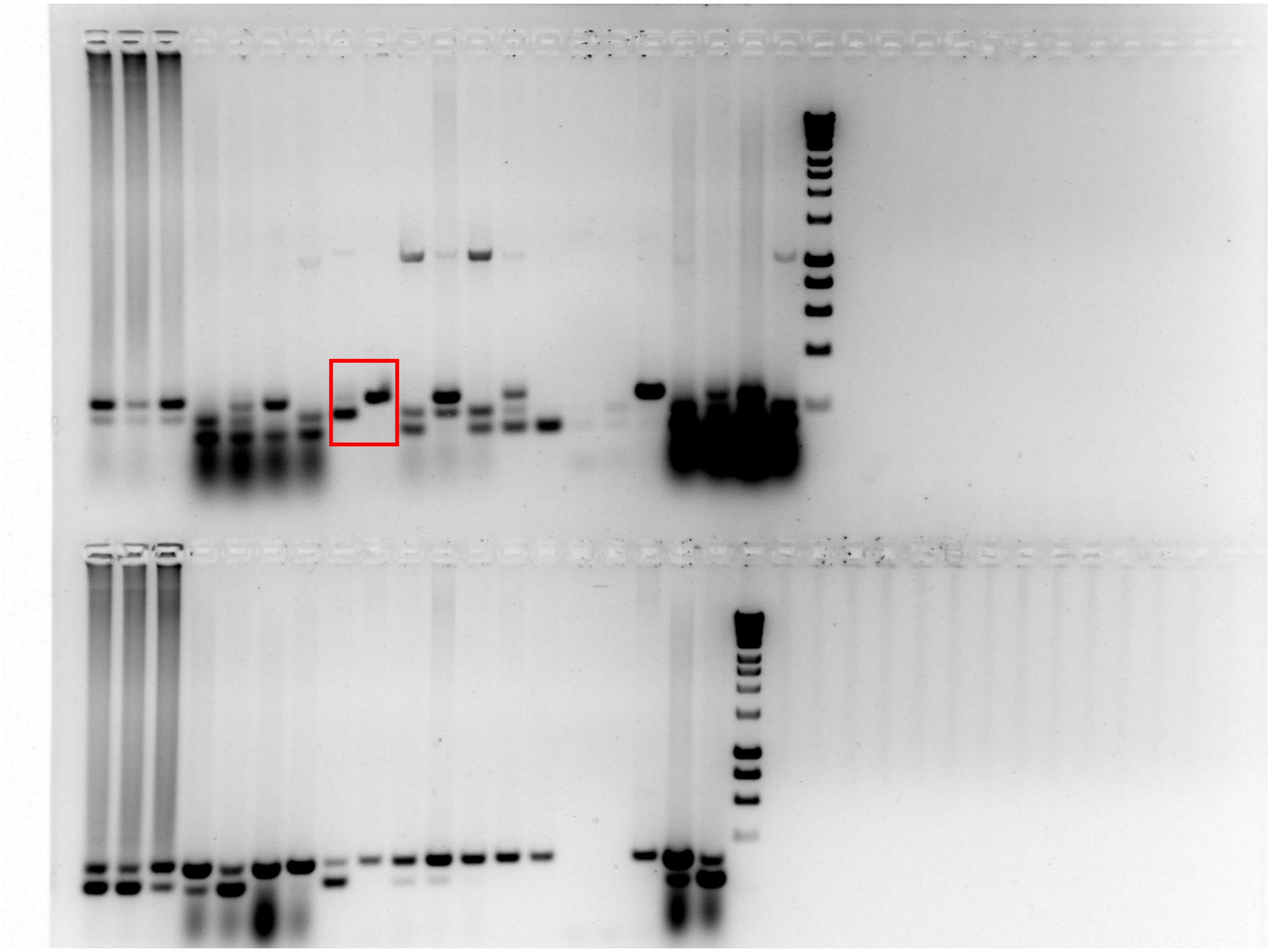

Full-size image of the agarose gel shown in Fig.5a

GFP

SEPT7

Ponceau

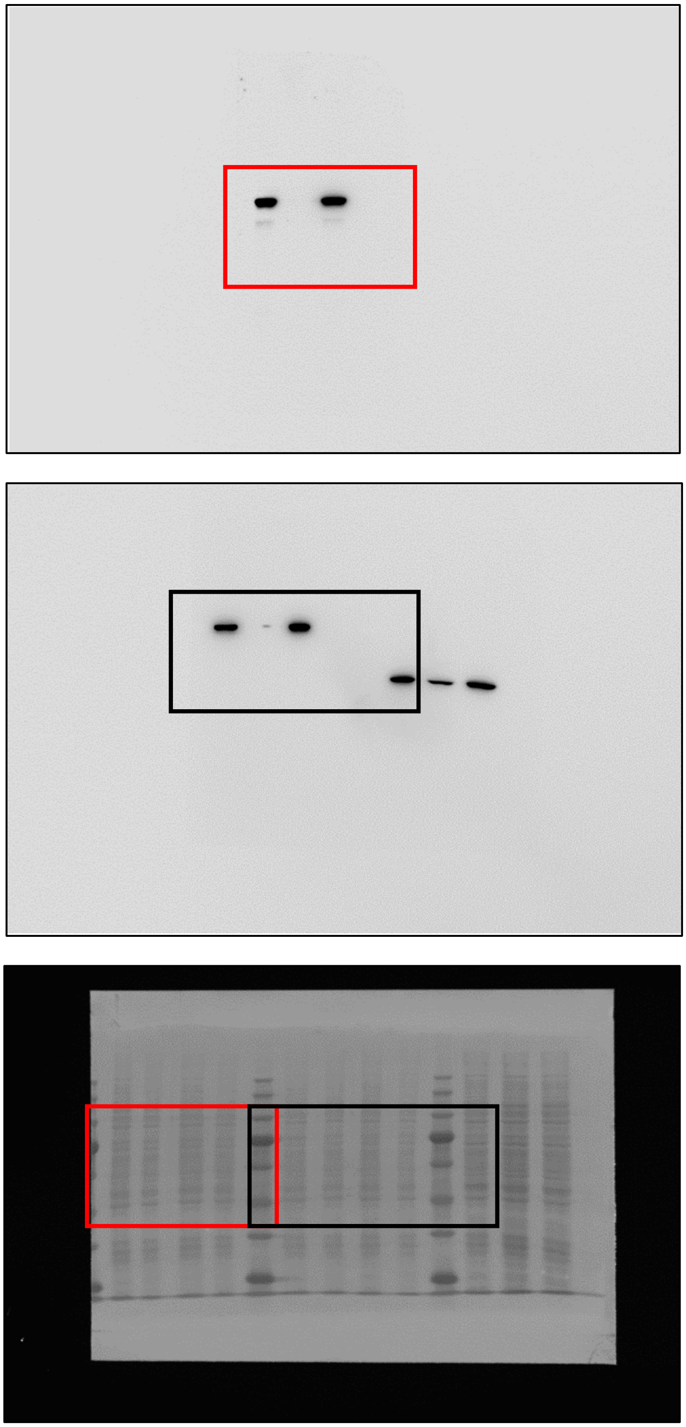

Full-size images of the blots shown in Fig.5b

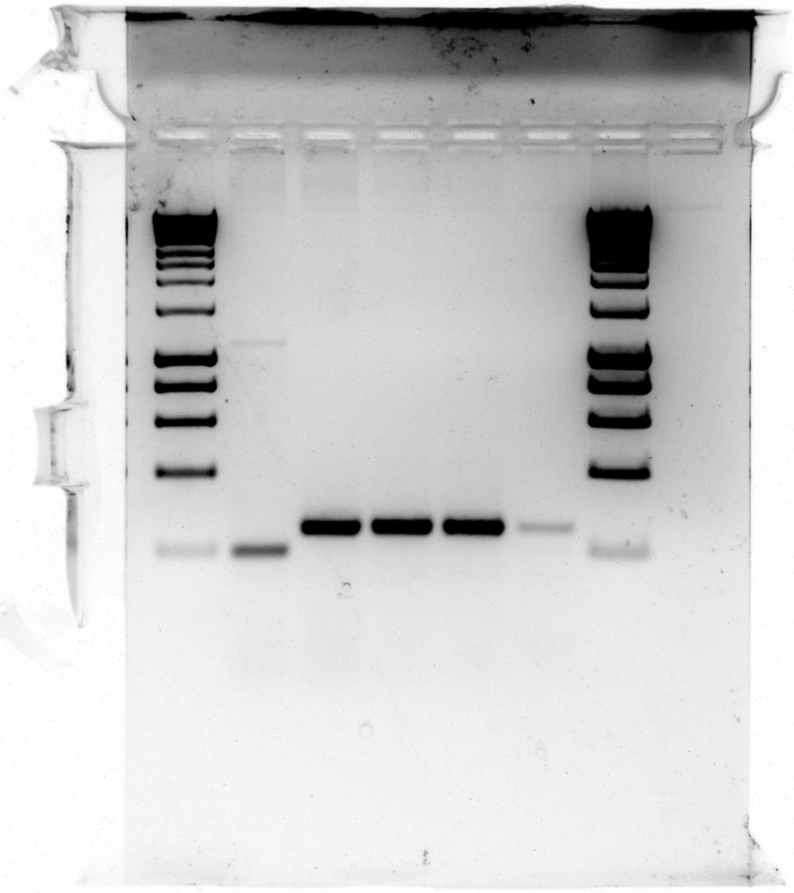

Full-size image of the agarose gel shown in Fig.6a

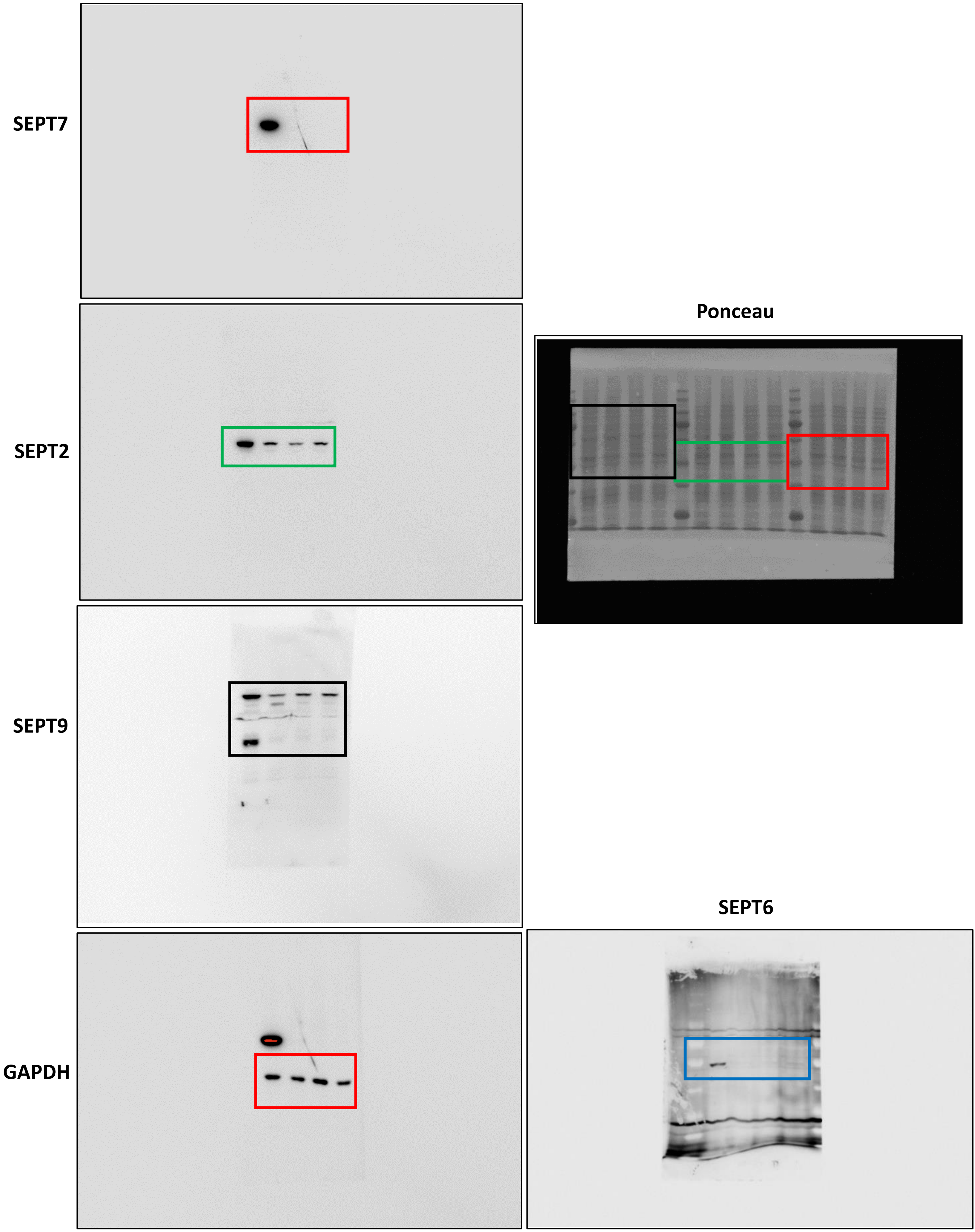

Full-size images of the blots shown in Fig.6b
