# Supplementary material for: Septin7 is essential in early hematopoiesis, but redundant at later stages
Source: Life Sci Alliance. 2026 Jul 13;9(10):e202603637. doi: 10.26508/lsa.202603637 (PMC13365248; doi:10.26508/lsa.202603637)
Supplement: Reviewer comments [file LSA-2026-03637_review_history.pdf]

Septin7 is essential in early hematopoiesis, but redundant at later stages

Natalia Ronkina, Nikita Verheyden, Prerna Gambhir, Kathrin Laass, Tatiana Yakovleva, Anneke Doerrie, Megha Abbey, Manoj Menon, Anton Selich, Melanie Galla, Michael Rothe, Axel Schambach, Stephanie Buchheister, Andreas Krueger, Alexey Kotlyarov, and Matthias Gaestel
DOI: https://doi.org/10.26508/lsa.202603637

Corresponding author(s): Natalia Ronkina, MHH

Review Timeline:

Submission Date:	2026-01-26
Editorial Decision:	2026-03-06
Revision Received:	2026-05-20
Editorial Decision:	2026-06-22
Revision Received:	2026-06-24
Accepted:	2026-07-01

Scientific Editor: Sarita Hebbar

Transaction Report:

(Note: With the exception of the correction of typographical or spelling errors that could be a source of ambiguity, letters and reports are not edited. The original formatting of letters and referee reports may not be reflected in this compilation.)

March 6, 2026

Re: Life Science Alliance manuscript #LSA-2026-03637-T

Dr. Natalia Ronkina
MHH
Biochemistry
Carl-Neuberg-Straße 1
Hannover, Lower Saxony 30625
Germany

Dear Dr. Ronkina,

Thank you for submitting your manuscript entitled "Septin7 is essential in early hematopoiesis, but redundant at later stages" to Life Science Alliance.

Your manuscript was assessed by three reviewers whose comments are appended below. As you will read, all the reviewers note the importance of your study on extending our understanding on the consequences of Septin 7 loss in hematopoietic populations. That said they have also raised concerns that (1) your major claims are not sufficiently supported with evidence and (2) a more nuanced discussion is needed. We agree with the reviewers that these concerns preclude publication of this work for now.

In line with their general evaluation, we invite you to submit a revised manuscript addressing their concerns. We agree that a revised manuscript must address points raised by the reviewers on:
-Selection pressure/heterogenity of Sept7 expression
-Repopulation in the chimera assay
-Characterisation of Sept7flox/flox Hoxb8 cells
-Improving the discussion

When submitting the revision, please include a letter addressing the reviewers' comments point by point. While a rebuttal must respond to all points in some form, additional data beyond those mentioned above are not required in a revision.

I would be happy to discuss the revision in more detail via email or phone/videoconferencing. Please let me know which option you prefer, if any.

To upload the revised version of your manuscript, please log in to your account: https://lsa.msubmit.net/cgi-bin/main.plex

You will be guided to complete the submission of your revised manuscript and to fill in all necessary information. Please get in touch in case you do not know or remember your login name.

The typical timeframe for revisions is three months. Please note that papers are generally considered through only one revision cycle, so strong support from the referees on the revised version is needed for acceptance.

While you are revising your manuscript, please also attend to the below editorial points to help expedite the publication of your manuscript. Please direct any editorial questions to the journal office. When submitting the revision, please include a letter addressing the reviewers' comments point by point.

Thank you for this interesting contribution to Life Science Alliance. We hope that the comments below will prove constructive as your work progresses, and we are looking forward to receiving your revised manuscript.

Sincerely,

Sarita Hebbar, PhD
Scientific Editor
Life Science Alliance
http://www.lsajournal.org

A. THESE ITEMS ARE REQUIRED FOR REVISIONS

-- A letter addressing the reviewers' comments point by point.

-- An editable version of the final text (.DOC or .DOCX) is needed for copyediting (no PDFs).

-- High-resolution figure, supplementary figure and video files uploaded as individual files: See our detailed guidelines for preparing your production-ready images, https://www.life-science-alliance.org/authors

-- Summary blurb (enter in submission system): A short text summarizing in a single sentence the study (max. 200 characters including spaces). This text is used in conjunction with the titles of papers, hence should be informative and complementary to the title and running title. It should describe the context and significance of the findings for a general readership; it should be written in the present tense and refer to the work in the third person. Author names should not be mentioned.

-- By submitting a revision, you attest that you are aware of our payment policies found here: https://www.life-science-alliance.org/copyright-license-fee

B. MANUSCRIPT ORGANIZATION AND FORMATTING:

Full guidelines are available on our Instructions for Authors page, https://www.life-science-alliance.org/authors

We encourage our authors to provide original source data, particularly uncropped/-processed electrophoretic blots and spreadsheets for the main figures of the manuscript. If you would like to add source data, we would welcome one PDF/Excel-file per figure for this information. These files will be linked as supplementary "Source Data" files.

IMPORTANT: It is Life Science Alliance policy that if requested, original data images must be made available. Failure to provide original images upon request will result in unavoidable delays in publication. Please ensure that you have access to all original microscopy and blot data images before submitting your revision.

Reviewer #1 (Comments to the Authors (Required)):

The manuscript from Ronkina N et al. expores the role of Septin 7 in hematopoiesis. Septin7 has been shown to play crucial role in cytokinesis in many cell types with Septin7-null mice showing embryo lethality. Previous work from this group reported that lymphoid- or myeloid-specific Sept7 knockouts have normal blood cell production. However, Sept7-deficient hematopoietic stem cells (HSCs) showed poor engraftment upon transplantation. To resolve this paradox, the authors extend the exploration of the role of Septin in different stages of hematopoietic diffrerentiation. The authors used hCD2-iCre (for lymphoid lineage) line and Vav1-iCre (pan-hematopoietic lineage) for cell-specific deletion of Sept7 in mice. They also generated Hoxb8-immortalized hematopoietic progenitors with inducible Sept7 deletion and explored the function in vitro. The manuscript presents some new data that will be useful to tackle the question in a but more detail. The contrast between the Vav-iCre and hCD2 mediated deletion is very informative and show a stage specific role of Septin7 that might not be linked to cytokinesis. However, key questions still remain and would be important to address. Major concern is that most of the conclusions are made on the basis of partial deletion of the gene at best.

1. Vav1-iCre::Sept7flox/flox animals showed incomplete deletion of the floxed Sept7 allele in bone marrow, thymus and spleen. PCR genotyping and Western blots revealed that most hematopoietic cells retained Sept7 expression despite Cre presence. In contrast, heterozygous (Sept7wt/flox) littermates showed complete excision of the floxed allele, indicating that cells with one intact Sept7 allele dominated the HSC pool pool. The authors interpret this as strong selection pressure in early hematopoiesis against the loss of Sept7. Importantly, T-cell development appeared normal in these mice (similar thymocyte subsets and peripheral T/B cell frequencies, presumably because only cells that escaped recombination survived. With intact protein expression in both genotypes, concluding a role for Septin7 is really hard. Do the authors see expression of Septin 7 in all HSCs? If there are HSCs lacking Septin7 expression, a competitive repopulation experiments will be informative.
2. There seems to be little evidence that the proportion of Sept7 +ive and -ive cells competed. Can the authors examine the heterogeneity of Sept7 expression in HSC population.
3. It will also be very informative if Sept7 deletion could be tested in fetal liver derived HSCs.
4. It is not very clear, how the results from Vav-iCre mediated deletion of Sept7 reported by the same group (Kandi R et al. 2021) stand against the results presented in this manuscript.
5. Contrasting statement about Septin7 expression are made in line 113-114 and 121-122. In fact, as mentioned in line 113-114, the deletion of Septin7 in DN3a cells is only partial. Therefore, it is hard to interpret the results.
6. How do the authors explain a significantly better deletion of the gene in DP cells onwards.
7. Was any defect in any terminally differentiated cell type observed following hCD2-iCre or Vav1-iCre mediated deletion of Sept7? Bloodm cell counts could add value to the results presented.
8. Generation of Sept7flox/flox Hoxb8 cells expressing Cre under doxycycline control resulted in complete deletion of both alleles and an absence of Sept7 protein. However, the Sept7-null progenitors proliferate normally. In this light how do the authors see their hypothesis of selection pressure on Sept7-null cells?
9. Do these cells with Sept7 deletion home and engraft well? In fact, it will be informative to test if Sept7-null HSCs are not

retained well in the BM. Do the authors see activation of extramedullary hematopoiesis in spleen?
10. Do the Sept7-null cells differentiate normally? Can the authors perform myeloid and lymphoid colony assays to test this?
11. The suggestion of DNA methylation at LoxP sites is intriguing. Can methylation status of the floxed Sept7 locus in escapee cells tested?
12. Overall, a direct evidence of the importance of Sept7 in early stages of hematopoiesis is missing. The conclusions are drawn mostly on the basis of expression of Sept7 in a heterogeneous BM cell population despite an efficient Vav-iCre mediated deletion.
13. The fundamental question remains if Septin7 was linked to cytokinesis at all in hematopoiesis. If the proliferation of HSCs lacking Sept7 expression is normal what seems to be the function of Sept7 in general? Can the authors comment on how it connects to the differentiation process?
Minor comments:
1. In the introduction section, it will be informative if the authors could add the genotype of the mouse lines for conditional and cell-specific deletion of septin 7.
2. Results presented in many figures lack quantification and statistical testing. Information such as number of repeats is missing in most of the results.
3. The HoxB8 cells are not the primitive HSCs so the results must be seen in that light.
4. Can some of the results presented point towards compensation from other septin proteins?

Reviewer #2 (Comments to the Authors (Required)):

This manuscript investigates the role of the cytoskeletal protein Septin7 in hematopoietic development and lineage-specific cytokinesis. Using conditional knockout mouse models (Vav1-iCre and hCD2-iCre) and an in vitro Hoxb8-immortalized progenitor system, the authors demonstrate that while Septin7 is essential for HSPCs and engraftment, it becomes redundant in later lymphoid and myeloid lineages. Through the observation of escaper clones and the use of inducible deletion models, they propose that the requirement for Septin7 is context-dependent, potentially linked to the mode of cell division. While the findings challenge the dogma that Septin7 is an obligate requirement for mammalian cytokinesis, the conclusions are weakened by the lack of mechanistic evidence regarding the escaper selection, insufficient exploration of compensatory Septin members, and the absence of direct visualization of polarity defects in HSCs. Additional biochemical profiling and high-resolution imaging of the cytoskeleton are needed to substantiate the proposed transition from essentiality to redundancy. Overall, the study provides significant insights into tissue-specific cytoskeletal requirements but requires major revision to support its core mechanistic claims.
Major comments
1. Figures 1-3: The authors show that Septin7-null HSCs fail to establish chimerism, and in the Vav1-iCre model, escaper clones completely dominate the hematopoietic system. However, is this because they cannot reach the niche, or because they fail at the first division post-entry? The authors should provide short-term homing assays (16-24h) and temporal flow cytometry data (Annexin V/7-AAD and Hoechst/Pyronin Y) on early HSPCs to determine if Septin7 is essential for the initial seeding of the bone marrow or for the subsequent cytokinesis and survival of the stem cell pool.
2. The authors attribute the engraftment defect primarily to cytokinesis failure, but septins are also key regulators of cortical tension and membrane mechanics. Given that primary HSCs must undergo extreme deformation during homing, impaired deformability or mechanical fragility could equally explain the failure of Septin7-null cells to engraft. The authors should directly test the biomechanical properties of mutant HSCs by using small-pore transwell migration assays or microfluidic deformation assays
3. The authors conclude that Septin7 is essential for early HSCs but redundant for lymphoid and myeloid lineages. The conclusion is based on steady-state observations (based on hCD2-iCre). However, cytoskeletal defects often only manifest under physiological stress. The authors should challenge the hCD2-iCre; Sept7 fl/fl mice with a 5-FU treatment or a viral challenge. Does the redundancy still hold when T or B cells are forced into rapid, stress-induced expansion? A competitive bone marrow transplantation assay using hCD2-iCre donors would also be a much more sensitive assay for subtle fitness defects.
4. The discrepancy between the in vitro success of Hoxb8 cells and the in vivo failure of HSCs needs a mechanistic explanation. The authors hypothesize that Septin7 might be specifically required for asymmetric cell division in HSCs, which is less critical in the symmetric expansion of Hoxb8 cells. To support this, the authors should perform immunofluorescence staining on Hoxb8 cells or primary HSPCs to examine the localization of polarity markers (e.g., CDC42, Scribble, or Numb) during mitosis.
5. Figures 3: The in vitro proliferation of Septin7-null Hoxb8 cells challenges the dogma of Septin-dependent cytokinesis. Is this due to compensatory mechanisms? The authors show that Septin2 and Septin9 levels are reduced (Fig. 6), but they do not rule out the compensatory upregulation of other Septins. The authors should perform RNA-seq or proteomic profiling on Septin7-null vs. WT Hoxb8 cells to check for the upregulation of Septin6, Septin8, or other cytoskeletal regulators. More importantly, they should use high-resolution microscopy to check if any filamentous Septin structures remain in Septin7-null Hoxb8 cells.
6. Figures 4-5: The escaper phenomenon in the Vav1-iCre model strongly suggests a lethal selective pressure, but the timing of this pressure is not well-defined. The authors should provide a more granular temporal analysis of the recombination efficiency across different progenitor stages (MPP, CMP, CLP).
7. The authors rule out a role for Septin7 in T-cell development based on the hCD2-iCre model, but does Septin7 regulate TCR signaling or immunological synapse formation? Cytoskeletal proteins are often crucial for the spatial organization of receptors. Even if T-cell numbers are normal, their functionality might be impaired. The authors should include data on T-cell activation

(CD69/CD25 upregulation after anti-CD3/CD28 stimulation) and synapse formation.

Minor comments
1. Figure 6 & S1: The Western Blot data shows a significant reduction in Septin2 and Septin9 proteins upon Septin7 deletion. The authors should clarify if this is due to protein instability or transcriptional downregulation by providing RT-qPCR data.

2. Figure S2: The frequencies of thymocyte subsets (DN3a, DN3b, DN4) are shown, but the absolute cell numbers are missing. Since Septin7 is implicated in cytokinesis, a subtle reduction in total cellularity might be masked by looking only at percentages.

3. Some experiments appear to have a small sample size (n=2 in Figure 1, S1, S2). For biological replicates, an n of at least 5 is generally expected in hematopoietic studies to account for individual mouse variability. The authors should justify the small n or supplement these datasets.

Estimated timeframe: The additional in vivo and mechanistic experiments requested above would likely require several months (approximately 3-6 months) to complete.

Reviewer #3 (Comments to the Authors (Required)):

Septin7 is essential in early hematopoiesis, but redundant at later stages
Natalia Ronkina, Nikita A. Verheyden, Prerna Gambhir, Kathrin Laaß, Tatiana Yakovleva, Anneke Dörrie, Megha Abbey, Manoj B. Menon, Anton Selich, Melanie Galla, Michael Rothe, Axel Schambach, Andreas Krueger, Alexey Kotlyarov, Matthias Gaestel

The manuscript investigates the consequences of Sept7 loss on early haematopoietic progenitors and downstream lymphoid populations.
I consider it important to report negative and unexpected data, as these findings add transparency and context to a study's conclusions. However, many phenotypes associated with Sept7 disruption have been reported previously. To strengthen the rationale and overall impact, the study would benefit from a clearer articulation of why further interrogation of Sept7 was necessary, and-crucially-what novel insights this work provides beyond existing knowledge. Highlighting these specific contributions would help clarify the unique value of the present study.

The authors should clarify whether the samples presented in Figure 1c (splenocytes, thymocytes, and bone marrow) were collected from the same individual mice.
The manuscript states that thymocytes show normal T cell development. Are these thymocytes expressing SEPT7? How do the authors confirm that the thymic cells analysed are derived from Sept7-null progenitors rather than WT cells?
The western blot in Figure 1b appears cropped. The authors should explain the rationale for cropping, provide full blots in supplementary material, and clearly indicate all included lanes.
Has Sept7 deletion efficiency been quantified for each mouse shown in Figure 1d? If so, these data should be presented or referenced.
Figures throughout the manuscript require more coherent annotation. When multiple mice are shown, the corresponding rows or plots should be labelled with mouse identifiers. Axis labels should be clearer, and the authors should define terms such as "normalised to mode" and "max %". For transparency, actual values (e.g., percentages, cell numbers) should be provided.
Figures 1e and 2d do not appear to add value to the central narrative and can be removed without loss of clarity.
The two cell populations within Figure 5e should be displayed using distinguishable colours rather than grey and white, which are difficult to differentiate. Even if the conclusion is that the populations are similar, this should be visually apparent from the figure.
Axis labels (Figure 5e) should include the fluorophore names rather than only wavelengths to improve interpretability.
In the Methods section (lines 326, 330, and 333), "Floxt-3L" should be corrected to "Flt-3L".
The Hoxb8-Fl system typically supports multilineage potential. Have the authors characterised the Hoxb8-Septflox/flox cells to confirm whether they retain multilineage output, or have they inadvertently selected for cells with lymphoid bias?
The manuscript would benefit from overall simplification and clarification. Several sections are unnecessarily complex and obscure the main findings.

Several claims are speculative and not supported by the presented data. Examples include:
"Incomplete deletion of SEPT7 from hematopoietic cells might indicate impaired repopulation activity... and selective advantages of SEPT7-expressing cells."
No evidence is provided for repopulation defects or selective advantage. Alternative explanations (e.g., cell death or failure to proliferate) are equally plausible.

"These observations suggest a requirement of SEPT7 for early stages of hematopoiesis and redundancy at later stages..."
The authors examine primarily lymphoid cells, which can differentiate without extensive cell cycling. The statement should be made population-specific rather than applying to "later stages of hematopoiesis" broadly.

"The distinct kinetics of HSC self-renewal across life stages may explain the essential role of SEPT7 in early but not later hematopoiesis."
This statement is not connected to the preceding discussion and appears inconsistent with the data. The authors do not examine fetal hematopoiesis, nor do they detect Sept7 deletion in adult bone marrow cells (Figure 1), making this interpretation unsupported.

"At later stages... the role of SEPT7 in cell division becomes redundant."
No evidence is provided to support redundancy of SEPT7 in proliferating hematopoietic populations. Hematopoietic cells continue to divide to maintain homeostasis. The authors should clarify that they are referring to specific cell types rather than the hematopoietic system as a whole.

Dear Dr. Hebbar,

Thank you for your letter and for the reviewers' comments for our manuscript entitled "Septin7 is essential in early hematopoiesis, but redundant at later stages" (Manuscript ID: #LSA-2026-03637-T). Herewith, we resubmit our revised manuscript for further consideration as an Original Research Article in Life Science Alliance.

We would like to sincerely thank the reviewers for their constructive comments and valuable suggestions, which helped us to improve the quality and clarity of our work.

We have made substantial efforts to improve the coherence, clarity, and overall quality of the manuscript. Our revision strategy was guided by the major concerns and criticisms raised during the review process. We believe that the extensive set of new experiments performed over the past three months has enabled us to comprehensively address the key points raised by all reviewers.

The principal changes and additions to the manuscript are summarized below:

1. Analysis of Septin7 expression in fetal liver cells from Vav1-iCre::Sept7flox/flox versus control embryos.
2. Analysis of Septin7 expression in primary HSPCs isolated from Vav1-iCre::Sept7flox/flox and Vav1-iCre::Sept7wt/flox mice.
3. Analysis of peripheral blood cell composition in Vav1-iCre::Sept7flox/flox and Vav1-iCre::Sept7wt/flox mice.
4. Analysis of Septin7 expression in peripheral blood cells from Vav1-iCre::Sept7flox/flox and Vav1-iCre::Sept7wt/flox mice.
5. Extended analyses of Septin7 expression in splenocytes and bone marrow cells from Vav1-iCre::Sept7flox/flox and Vav1-iCre::Sept7wt/flox mice.
6. Use of a mouse model with selective activation of oncogenic K-Ras in hCD2-iCre–expressing cells.
7. Staining of Hoxb8-HSPCs with the polarity marker CDC42.
8. Ex vivo activation of T cells isolated from hCD2-iCre::Sept7flox/flox and hCD2-iCre::Sept7wt/flox mice using Dynabeads® Mouse T-Activator CD3/CD28.
9. Inclusion of extensive statistical analyses in the Supplementary Information section.

We have carefully addressed all concerns raised during the review process and revised the manuscript accordingly. All changes introduced in the revised version have been highlighted in red.

Below, we provide a detailed point-by-point response to the reviewers' comments.

Kind regards,

Natalia Ronkina
Corresponding author
E-mail: ronkina.natalia@mh-hannover.de

Point-by-point response to the reviewers' comments

Reviewer #1 (Comments to the Authors (Required)):

The manuscript from Ronkina N et al. explores the role of Septin 7 in hematopoiesis. Septin7 has been shown to play crucial role in cytokinesis in many cell types with Septin7-null mice showing embryo lethality. Previous work from this group reported that lymphoid- or myeloid-specific Sept7 knockouts have normal blood cell production. However, Sept7-deficient hematopoietic stem cells (HSCs) showed poor engraftment upon transplantation. To resolve this paradox, the authors extend the exploration of the role of Septin in different stages of hematopoietic diffrerentiation. The authors used hCD2-iCre (for lymphoid lineage) line and Vav1-iCre (pan-hematopoietic lineage) for cell-specific deletion of Sept7 in mice. They also generated Hoxb8-immortalized hematopoietic progenitors with inducible Sept7 deletion and explored the function in vitro. The manuscript presents some new data that will be useful to tackle the question in a but more detail. The contrast between the Vav-iCre and hCD2 mediated deletion is very informative and show a stage specific role of Septin7 that might not be linked to cytokinesis. However, key questions still remain and would be important to address. Major concern is that most of the conclusions are made on the basis of partial deletion of the gene at best.

We thank Reviewer #1 for the helpful and constructive evaluation of our manuscript. Our answers to the specific questions of the reviewer are below:

1. Vav1-iCre::Sept7flox/flox animals showed incomplete deletion of the floxed Sept7 allele in bone marrow, thymus and spleen. PCR genotyping and Western blots revealed that most hematopoietic cells retained Sept7 expression despite Cre presence. In contrast, heterozygous (Sept7wt/flox) littermates showed complete excision of the floxed allele, indicating that cells with one intact Sept7 allele dominated the HSC pool. The authors interpret this as strong selection pressure in early hematopoiesis against the loss of Sept7. Importantly, T-cell development appeared normal in these mice (similar thymocyte subsets and peripheral T/B cell frequencies), presumably because only cells that escaped

recombination survived. With intact protein expression in both genotypes, concluding a role for Septin7 is really hard. Do the authors see expression of Septin 7 in all HSCs? If there are HSCs lacking Septin7 expression, a competitive repopulation experiments will be informative.

Answer: This is a very important question which we addressed in our previous study (Kandi et al. 2021). Competitive repopulation experiments performed with Vav1-iCre::Sept7flox/flox mice indicated, that hematopoietic stem cells generated from these mice failed to engraft and establish donor chimerism following transplantation, indicating that Septin7 is essential for hematopoietic stem cell function under stress conditions. We now performed additional experiments, where we analyze intracellular Sept7 expression in fetal liver and in adult freshly isolated HSPCs. We added the following information to the manuscript: "To test our hypothesis that SEPT7 is required during early hematopoiesis, we examined its expression in nucleated hematopoietic (CD45-positive) fetal liver cells. Vav1-iCre is strongly expressed and active in the fetal liver from embryonic day 9.5 (E9.5) to approximately E11.5 and later, enabling efficient Cre-mediated recombination in hematopoietic stem cells and all downstream hematopoietic lineages (He et al, 2008). At E14.5, we observed a marked reduction, although not a complete loss, of SEPT7 expression in CD45-positive fetal liver cells (new Figure 6F). Notably, approximately 20% of hematopoietic (CD45-positive) fetal liver cells remained SEPT7-positive at this stage, suggesting incomplete SEPT7 deletion in a subset of hematopoietic cells during embryonic development (new Figure S9). Interestingly, in the bone marrow of adult mice, approximately 46% of lineage-negative HSPCs remained SEPT7-positive, indicating a substantial increase in the proportion of SEPT7-expressing escapees among hematopoietic progenitors during ontogenesis (new Figures 6G and S10). Notably, the proportion of SEPT7-positive escapees further increased to 86.5% in the overall bone marrow cell population (modified Figure 1C and new Figure S1C), suggesting progressive enrichment of SEPT7-positive cells during hematopoiesis. This observation supports the existence of selective pressure against SEPT7 deficiency, potentially due to impaired long-term maintenance of SEPT7-deficient progenitors." We have incorporated this data into the main text in the section entitled "Generation of Hoxb8-immortalized Sept7 KO HSPCs."

2. There seems to be little evidence that the proportion of Sept7 +ive and -ive cells competed. Can the authors examine the heterogeneity of Sept7 expression in HSC population.

Answer: This is a key question that we initially addressed in Fig. 3, where FACS analysis demonstrated SEPT7 expression in all Hoxb8-immortalized HSPCs derived from Vav1-iCre::Sept7flox/flox mice (Fig. 3c,e,f). Notably, in three independent immortalization experiments, we were unable to establish Hoxb8-immortalized cultures from SEPT7-deficient HSPCs. To further investigate this observation, we performed an additional experiment analyzing intracellular SEPT7 expression in freshly isolated lineage-negative primary HSPCs (see our response to the previous question). Surprisingly, approximately 54% of primary lineage-negative HSPCs were SEPT7-negative. Thus, while SEPT7-deficient HSPCs are clearly present in the primary progenitor compartment, they appear to be strongly underrepresented following Hoxb8-mediated immortalization. These findings suggest that SEPT7-positive progenitors are preferentially immortalized under Hoxb8 culture conditions. One possible explanation is that SEPT7 deficiency compromises ex vivo proliferative potential or survival during the immortalization process, even though SEPT7

is dispensable for steady-state proliferation once immortalized progenitor lines have been established.

3. It will also be very informative if Sept7 deletion could be tested in fetal liver derived HSCs.

Answer: We fully agree with the reviewer that this information would be highly valuable. We have now performed additional experiments assessing Sept7 deletion in hematopoietic (CD45-positive) fetal liver cells. These new data have been incorporated into the main text of the manuscript in the section "Generation of Hoxb8-immortalized Sept7 KO HSPCs". See our response to the first point of reviewer 1.

4. It is not very clear, how the results from Vav-iCre mediated deletion of Sept7 reported by the same group (Kandi R et al. 2021) stand against the results presented in this manuscript.

Answer: We thank the reviewer for pointing on this aspect. Our current results do not contradict with, but rather extend our previously published results. In Kandi R et al. 2021 we demonstrated that general hematopoiesis in Vav1-iCre::Sept7flox/flox mice in steady state conditions is almost unaffected. Upon competitive transplantation HSCs derived from Vav1-iCre::Sept7flox/flox mice revealed a lack of repopulation potential, and a skewing of differentiation, with enhanced lymphoid differentiation at the cost of myeloid cells and a higher frequency of LT-HSCs at the cost of the frequency of LMPPs. The supplementary Figure EV4 D of the above publication shows results of genotyping of lineage negative cells derived from Vav1-iCre::Sept7flox/flox mice demonstrating incomplete deletion of the Sept7 fl/fl allele which we observe also in our current work. We suppose that reduced repopulation capacity of HSCs derived from Vav1-iCre::Sept7flox/flox demonstrated in our previous study could be explained by exhaustion of repopulation capacity of HSCs via a selection process against Sept7 deficiency, leading to survival of Sept7 retaining cells. We extended our discussion to clarify this point more deeply.

5. Contrasting statement about Septin7 expression are made in line 113-114 and 121-122. In fact, as mentioned in line 113-114, the deletion of Septin7 in DN3a cells is only partial. Therefore, it is hard to interpret the results.

Answer: We thank the reviewer for this indication. We have corrected this part by replacing DN3a with DN3b.

6. How do the authors explain a significantly better deletion of the gene in DP cells onwards.

Answer: hCD2-iCre expresses active Cre in immature and mature B and T lymphocytes beginning at the common lymphoid progenitor stage (CLP) (de Boer et al. 2003). The significantly more efficient deletion of the Sept7 gene from the DP stage onward can be explained by the progressive nature of Cre-mediated recombination during T-cell development, beginning at the CLP stage. We included this passage now in the main text of the manuscript in the section "SEPT7 is effectively eliminated in T-cells from hCD2-iCre::Sept7flox/flox mice".

7. Was any defect in any terminally differentiated cell type observed following hCD2-iCre or Vav1-iCre mediated deletion of Sept7? Blood cell counts could add value to the results presented.

Answer: We agree with Reviewer #1 that analysis of peripheral blood cells would add value to the presented findings. Previous analysis of peripheral blood cell counts in hCD2-iCre::Sept7flox/flox mice (Figure S8 in Menon et al., 2014, PLoS Genetics) revealed no differences compared with hCD2-iCre::Sept7wt/wt mice, and we now reference these data in the manuscript. In addition, we performed new experiments comparing peripheral blood cell composition between Vav1-iCre::Sept7flox/flox and Vav1-iCre::Sept7wt/flox mice. These new data have been incorporated into the main text of the manuscript in the section "Hematopoietic cells escape Vav1-iCre-mediated recombination of the Sept7 flox/flox allele.": There we state: *"Analysis of peripheral blood revealed no differences in blood cell composition between Vav1-iCre::Sept7flox/flox and Vav1-iCre::Sept7wt/flox mice, suggesting that either SEPT7 is dispensable for the maintenance of steady-state hematopoiesis under homeostatic conditions or that hematopoiesis is progressively dominated by cells that have escaped Sept7 deletion. (new Figure S2)".*

8. Generation of Sept7flox/flox Hoxb8 cells expressing Cre under doxycycline control resulted in complete deletion of both alleles and an absence of Sept7 protein. However, the Sept7-null progenitors proliferate normally. In this light how do the authors see their hypothesis of selection pressure on Sept7-null cells?

Answer: Indeed, we were very surprised to see, that Sept7 deficient Hoxb8 immortalized HSPCs proliferate normally ex vivo. "Previous studies have shown that Hoxb8-immortalized HSPCs lose self-renewal capacity and megakaryocyte/erythroid differentiation potential while retaining myeloid and lymphoid potential (Redecke et al, 2013). The self-renewal capacity of hematopoietic stem cells (HSCs) is closely linked to cell polarity, which is characterized by the asymmetric distribution of polarity-associated proteins such as CDC42 (Florian et al, 2018). Consistent with these findings, both SEPT7-deficient and SEPT7-expressing Hoxb8-immortalized HSPCs exhibited a diffuse distribution of CDC42, including during cell division (new Figure S8B). Based on these observations, we propose that although SEPT7 is critical for maintaining cell polarity and HSC function (Kandi et al, 2021), it is dispensable for the proliferation of unpolarized progenitors such as Hoxb8-immortalized HSPCs." We included this passage now in the main text of the manuscript in the section "Generation of Hoxb8 immortalized Sept7 KO HSPCs".

9. Do these cells with Sept7 deletion home and engraft well? In fact, it will be informative to test if Sept7-null HSCs are not retained well in the BM. Do the authors see activation of extramedullary hematopoiesis in spleen?

Answer: We previously demonstrated that both Vav1-iCre::Sept7wt/flox and Vav1-iCre::Sept7flox/flox HSCs exhibit comparable homing efficiency to the bone marrow (Fig. EV5H in Kandi et al., 2021). In addition, we did not observe any differences in spleen size between Vav1-iCre::Sept7wt/flox and Vav1-iCre::Sept7flox/flox mice (new Figure S1A),

further arguing against the presence of splenic extramedullary hematopoiesis. We now discuss this point in the "Discussion" section.

10. Do the Sept7-null cells differentiate normally? Can the authors perform myeloid and lymphoid colony assays to test this?

Answer: Our previous study (Menon et al. 2014) demonstrated that Sept7 deficient leukocytes exhibited subnormal, but sufficient proliferative activity in (IL)-3/IL-6/SCF-dependent myeloid colony formation assay. In our current work we observe no differences in T-cell differentiation between hCD2-iCre::Sept7flox/flox and hCD2-iCre::Sept7wt/flox in thymus, as all thymocyte populations were comparable (Figure S4A). We mention this observation now in the main text of the manuscript in the section "SEPT7 is effectively eliminated in T-cells from hCD2-iCre::Sept7flox/flox mice".

11. The suggestion of DNA methylation at LoxP sites is intriguing. Can methylation status of the floxed Sept7 locus in escapee cells tested?

Answer: This is a very intriguing question. To address this issue, we compared DNA isolated from Hoxb8-HSPCs derived from Sept7flox/flox and Vav1-iCre::Sept7flox/flox mice. Genomic DNA was successfully subjected to bisulfite conversion using the EpiTect Plus DNA Bisulfite Kit (Qiagen, Cat. #59124). However, only one of the two LoxP site region flanking exon 4 of Sept7 could be amplified using primers specifically designed for bisulfite-treated DNA (https://www.zymoresearch.de/pages/bisulfite-primer-seeker). In addition, the sequencing quality of the resulting amplicons was insufficient to allow definitive conclusions regarding potential differences in the methylation status of regions surrounding the LoxP sites. Therefore, additional experiments will be required to fully address this question. While these analyses may provide important mechanistic insight into the regulation of Cre-mediated recombination at the Sept7 locus, their thorough investigation would require extensive additional work and is beyond the scope and revision timeline of the current manuscript.

12. Overall, a direct evidence of the importance of Sept7 in early stages of hematopoiesis is missing. The conclusions are drawn mostly on the basis of expression of Sept7 in a heterogeneous BM cell population despite an efficient Vav-iCre mediated deletion.

Answer: "Previous studies have shown that the Cdc42-Borg4-Septin7 axis is essential for maintaining polarity in HSCs, and that Septin7-deficient HSCs exhibit reduced engraftment potential along with characteristics of aged HSCs (Kandi et al. 2021). Short-term homing assays using HSCs derived from Vav1-iCre::Sept7flox/flox mice demonstrated that homing to the bone marrow is not impaired, indicating that defective homing is unlikely to account for the failure to establish donor chimerism (Kandi et al, 2021). In the current study, we provide evidence that indirectly supports an essential role for SEPT7 in HSC function, while demonstrating that SEPT7 is dispensable for in vivo lymphoid development and ex vivo proliferation of Hoxb8-immortalized progenitors. We show that SEPT7-expressing escapees arise as early as embryonic day 14.5, and that their proportion among lineage-negative hematopoietic progenitors increases during ontogenesis, indicating strong selection pressure against SEPT7 deficiency in hematopoietic progenitors. The inefficiency of Vav1-iCre–mediated Sept7flox/flox recombination, which could not be overcome by additional Cre expression, suggests that HSCs evade Cre-mediated deletion of Sept7. Accordingly, the

reduced engraftment of HSCs from Vav1-iCre::Sept7flox/flox mice (Kandi et al., 2021) likely reflects exhaustion of repopulating potential during selection against Sept7 loss." We now extended the discussion to better explain our conclusions.

13. The fundamental question remains if Septin7 was linked to cytokinesis at all in hematopoiesis. If the proliferation of HSCs lacking Sept7 expression is normal what seems to be the function of Sept7 in general? Can the authors comment on how it connects to the differentiation process?

Answer: In our previous study, we demonstrated that SEPT7 plays an important role in HSC polarity and function under stress conditions (Kandi et al., 2021). In the present study, we show that SEPT7 is dispensable for cytokinesis in Hoxb8-immortalized myeloid and lymphoid progenitors and is not essential for lymphoid development under either steady-state or physiological stress induced by oncogenic K-Ras–driven tumorigenesis. We propose that although SEPT7 is critical for maintaining cell polarity and HSC function (Kandi et al, 2021), it is dispensable for the proliferation of unpolarized progenitors such as Hoxb8-immortalized HSPCs. We have now expanded the Introduction and "Generation of Hoxb8 immortalized Sept7 KO HSPCs" sections accordingly.

Minor comments:

1. In the introduction section, it will be informative if the authors could add the genotype of the mouse lines for conditional and cell-specific deletion of septin 7.

Answer: We thank the reviewer for this point. We introduced the description of genotype of the mouse lines for conditional and cell-specific deletion of Sept7 in the introduction section.

2. Results presented in many figures lack quantification and statistical testing. Information such as number of repeats is missing in most of the results.

Answer: We thank the reviewer for this comment. We now indicate the number of independent experiments in the corresponding figure legends. In addition, we have included statistical analyses in the Supplementary Information.

3. The HoxB8 cells are not the primitive HSCs so the results must be seen in that light.

Answer: We agree with the reviewer and now state in the main text, within the section "Generation of Hoxb8-immortalized Sept7 KO HSPCs," that stable transduction of HSPCs with a Hoxb8-expressing construct promotes the proliferation of myeloid and lymphoid progenitors. In addition, new Figure S8B demonstrates that Hoxb8-immortalized HSPCs lack asymmetric distribution of polarity-associated proteins such as CDC42, indicating impaired cellular polarity, which is closely linked to the self-renewal capacity of primitive HSCs.

4. Can some of the results presented point towards compensation from other septin proteins?

Answer: This is an important point. As loss of SEPT7 is accompanied by a marked reduction in septins from all three remaining subgroups—SEPT2, SEPT6, and SEPT9—we conclude

that SEPT7 function is not compensated by other septins. We have now included the SEPT6 blot in the revised Figure 6B.

Reviewer #2 (Comments to the Authors (Required)):

This manuscript investigates the role of the cytoskeletal protein Septin7 in hematopoietic development and lineage-specific cytokinesis. Using conditional knockout mouse models (Vav1-iCre and hCD2-iCre) and an in vitro Hoxb8-immortalized progenitor system, the authors demonstrate that while Septin7 is essential for HSPCs and engraftment, it becomes redundant in later lymphoid and myeloid lineages. Through the observation of escaper clones and the use of inducible deletion models, they propose that the requirement for Septin7 is context-dependent, potentially linked to the mode of cell division. While the findings challenge the dogma that Septin7 is an obligate requirement for mammalian cytokinesis, the conclusions are weakened by the lack of mechanistic evidence regarding the escaper selection, insufficient exploration of compensatory Septin members, and the absence of direct visualization of polarity defects in HSCs. Additional biochemical profiling and high-resolution imaging of the cytoskeleton are needed to substantiate the proposed transition from essentiality to redundancy. Overall, the study provides significant insights into tissue-specific cytoskeletal requirements but requires major revision to support its core mechanistic claims.

We thank reviewer #2 for the overall positive assignment of our manuscript and the helpful comments to improve the manuscript.

Major comments

1. Figures 1-3: The authors show that Septin7-null HSCs fail to establish chimerism, and in the Vav1-iCre model, escaper clones completely dominate the hematopoietic system. However, is this because they cannot reach the niche, or because they fail at the first division post-entry? The authors should provide short-term homing assays (16-24h) and temporal flow cytometry data (Annexin V/7-AAD and Hoechst/Pyronin Y) on early HSPCs to determine if Septin7 is essential for the initial seeding of the bone marrow or for the subsequent cytokinesis and survival of the stem cell pool.

Answer: We thank the reviewer for raising this point. We performed short-term homing assays using HSPCs derived from our Vav1-iCre::Sept7flox/flox mice in a previous study (Kandi et al., 2021) and showed that homing to the bone marrow is not impaired, indicating that defective homing is unlikely to account for the failure to establish donor chimerism. We have now included this point in the Discussion.

2. The authors attribute the engraftment defect primarily to cytokinesis failure, but septins are also key regulators of cortical tension and membrane mechanics. Given that primary HSCs must undergo extreme deformation during homing, impaired deformability or mechanical fragility could equally explain the failure

of Septin7-null cells to engraft. The authors should directly test the biomechanical properties of mutant HSCs by using small-pore transwell migration assays or microfluidic deformation assays.

Answer: This is an interesting point. To address it, we performed three independent transwell migration assays using 8 µm and 3 µm pore sizes with Hoxb8-immortalized HSPCs (Sept7fl/fl versus Sept7 knockout). In the 8 µm assay, we observed a trend toward higher migration efficiency in Sept7-expressing cells compared with Sept7-deficient cells, although this difference did not reach statistical significance (30.0% ± 15.3% vs. 18.8% ± 6.8% of initially seeded cells, respectively). Unexpectedly, in the 3 µm pore assay, Sept7-expressing Hoxb8-HSPCs showed significantly lower migration efficiency than Sept7 knockout cells (0.01% ± 0.007% vs. 0.06% ± 0.021%, respectively; P = 0.002 in two-tailed Welch's t-test). At present, we do not have a mechanistic explanation for this inverse effect in the smaller pore size condition. Given the preliminary nature of these findings and the lack of mechanistic clarity, we decided not to include these data in the current manuscript.

3. The authors conclude that Septin7 is essential for early HSCs but redundant for lymphoid and myeloid lineages. The conclusion is based on steady-state observations (based on hCD2-iCre). However, cytoskeletal defects often only manifest under physiological stress. The authors should challenge the hCD2-iCre; Sept7 fl/fl mice with a 5-FU treatment or a viral challenge. Does the redundancy still hold when T or B cells are forced into rapid, stress-induced expansion? A competitive bone marrow transplantation assay using hCD2-iCre donors would also be a much more sensitive assay for subtle fitness defects.

Answer: We thank the reviewer for this important suggestion. To mimic physiological stress in lymphoid progenitors, we employed a mouse model with selective activation of oncogenic K-Ras in hCD2-iCre–expressing cells. Expression of oncogenic K-Ras-G12D in hCD2-iCre–expressing cells induced splenomegaly in both K-Ras-G12D::hCD2-iCre::Sept7flox/flox and K-Ras-G12D::hCD2-iCre::Sept7wt/flox mice (new Figure S5A). Nevertheless, K-Ras-G12D::hCD2-iCre::Sept7flox/flox mice showed efficient depletion of SEPT7 in both T and B cells (new Figures S5B and S6A). However, we did not observe any differences in T- or B-cell development between K-Ras-G12D::hCD2-iCre::Sept7flox/flox and K-Ras-G12D::hCD2-iCre::Sept7wt/flox mice, either in the thymus or in the spleen (new Figures S5C and S6C). This observation led us to conclude that SEPT7 is not essential for lymphoid cell development, even under conditions of physiological stress. We have now included these data in the supplementary section and discuss them in the main text of the manuscript in the section "SEPT7 is effectively eliminated in T-cells from hCD2-iCre::Sept7flox/flox mice".

4. The discrepancy between the in vitro success of Hoxb8 cells and the in vivo failure of HSCs needs a mechanistic explanation. The authors hypothesize that Septin7 might be specifically required for asymmetric cell division in HSCs, which is less critical in the symmetric expansion of Hoxb8 cells. To support this, the authors should perform immunofluorescence staining on Hoxb8 cells or primary HSPCs to examine the localization of polarity markers (e.g., CDC42, Scribble, or Numb) during mitosis.

Answer: We thank the reviewer for this valuable suggestion. Since we were unable to achieve efficient Sept7 knockout in primary HSPCs, we analyzed the distribution of the polarity marker CDC42 in Hoxb8-immortalized HSPCs. The self-renewal capacity of hematopoietic stem cells (HSCs) is closely linked to cell polarity, which is characterized by the asymmetric distribution of polarity-associated proteins such as CDC42 (Florian et al., 2018). Previous studies have shown that Hoxb8-immortalized HSPCs lose self-renewal capacity and megakaryocyte/erythroid differentiation potential while retaining myeloid and lymphoid potential (Redecke et al., 2013). Consistent with these findings, both SEPT7-deficient and SEPT7-expressing Hoxb8-immortalized HSPCs exhibited a diffuse distribution of CDC42, including during cell division (new Figure S8B). Based on these observations, we propose that although SEPT7 is critical for maintaining cell polarity and HSC function (Kandi et al., 2021), it is dispensable for the proliferation of unpolarized progenitors such as Hoxb8-immortalized HSPCs. We have now included these data in the supplementary material and incorporated their discussion into the main text in the section entitled "Generation of Hoxb8-immortalized Sept7 KO HSPCs."

5. Figures 3: The in vitro proliferation of Septin7-null Hoxb8 cells challenges the dogma of Septin-dependent cytokinesis. Is this due to compensatory mechanisms? The authors show that Septin2 and Septin9 levels are reduced (Fig. 6), but they do not rule out the compensatory upregulation of other Septins. The authors should perform RNA-seq or proteomic profiling on Septin7-null vs. WT Hoxb8 cells to check for the upregulation of Septin6, Septin8, or other cytoskeletal regulators. More importantly, they should use high-resolution microscopy to check if any filamentous Septin structures remain in Septin7-null Hoxb8 cells.

Answer: We thank the reviewer for this suggestion. We performed additional analyses of SEPT6 and SEPT8 expression. Unfortunately, using the polyclonal goat anti-SEPT8 antibody (sc-48937), we were unable to detect SEPT8 expression in any of the analyzed cell lines except HEK293 cells. Consistent with previous observations (Menon et al., 2014), loss of SEPT7 in Hoxb8-HSPCs was accompanied by a marked reduction in all septins representing the remaining major septin subgroups, including SEPT2, SEPT6, and SEPT9 (modified Figure 6B). These findings indicate that SEPT7 deficiency is not compensated by upregulation or stabilization of members of other septin subgroups. We are highly interested in identifying the compensatory mechanisms that permit proper cell division in the absence of SEPT7. However, elucidating these mechanisms will require extensive additional investigation and is beyond the scope and revision timeline of the current manuscript.

6. Figures 4-5: The escaper phenomenon in the Vav1-iCre model strongly suggests a lethal selective pressure, but the timing of this pressure is not well-defined. The authors should provide a more granular temporal analysis of the recombination efficiency across different progenitor stages (MPP, CMP, CLP).

Answer: We performed the analyses of the bone marrow composition in Vav1-iCre::Sept7flox/flox mice in our earlier study (Kandi et al. 2021). We observed normal frequencies of B-,T-, and myeloid cells with a slightly elevated frequency of ST-HSCs but not LT-HSCs, and a reduced frequency of LMPPs and elevated frequency of CLPs (Figs 4E, F and EV5C, Appendix Fig S1I in Kandi et al. 2021). Additionally we performed new experiments

where we compared peripheral blood cell composites between Vav1-iCre::Sept7flox/flox and Vav1-iCre::Sept7wt/flox mice. Intracellular SEPT7 protein expression assessed by flow cytometry demonstrated, that most of peripheral blood cells from Vav1-iCre::Sept7flox/flox mice still contain SEPT7 (modified Figure 1C and new Figure S1). Analysis of peripheral blood revealed no differences in blood cell composition between Vav1-iCre::Sept7flox/flox and Vav1-iCre::Sept7wt/flox mice, suggesting that either SEPT7 is dispensable for the maintenance of steady-state hematopoiesis under homeostatic conditions or that hematopoiesis is progressively dominated by cells that have escaped Sept7 deletion (new Figure S2).

7. The authors rule out a role for Septin7 in T-cell development based on the hCD2-iCre model, but does Septin7 regulate TCR signaling or immunological synapse formation? Cytoskeletal proteins are often crucial for the spatial organization of receptors. Even if T-cell numbers are normal, their functionality might be impaired. The authors should include data on T-cell activation (CD69/CD25 upregulation after anti-CD3/CD28 stimulation) and synapse formation.

Answer: We thank the reviewer for this important suggestion. We analyzed the ex vivo proliferation of T cells isolated from hCD2-iCre::Sept7flox/flox mice following stimulation with T-Cell Activator CD3/CD28 for T-cell activation and expansion. Compared with hCD2-iCre::Sept7wt/flox controls, hCD2-iCre::Sept7flox/flox T cells displayed delayed proliferation. PCR genotyping, western blot analyses and immunofluorescence staining demonstrated that this impaired expansion was associated with the selective outgrowth of SEPT7-expressing cells (new Figure S7). These findings are consistent with the study by Mujal et al. 2016, which demonstrated that SEPT7 is required for cytokine-driven autonomous ex vivo *T-cell proliferation, but is dispensable for contact-dependent division mediated by antigen-presenting cells. We have now included these data in the supplementary material and incorporated their discussion into the main text in the section entitled "Generation of Hoxb8-immortalized Sept7 KO HSPCs." SEPT7 is effectively eliminated in T-cells from hCD2-iCre::Sept7flox/flox mice".*

Minor comments
1. Figure 6 & S1: The Western Blot data shows a significant reduction in Septin2 and Septin9 proteins upon Septin7 deletion. The authors should clarify if this is due to protein instability or transcriptional downregulation by providing RT-qPCR data.

Answer: We thank the reviewer for this insightful comment and have revised the manuscript text to clarify these points. The mutual stabilization of septins from different groups through protein–protein interactions is well established, and we now cite the relevant previous studies to better explain the findings shown in modified Figure 6b.

2. Figure S2: The frequencies of thymocyte subsets (DN3a, DN3b, DN4) are shown, but the absolute cell numbers are missing. Since Septin7 is implicated in cytokinesis, a subtle reduction in total cellularity might be masked by looking only at percentages.

Answer: We thank the reviewer for this valuable suggestion. Unfortunately, absolute cell numbers were not determined in the original experiments. In addition, due to the limited time available for revision of the manuscript, we were unable to repeat the full set of animal experiments to address this point. We now mention in the discussion of these results that "subtle changes in total cellularity may be masked when analyzing percentages alone".

3. Some experiments appear to have a small sample size (n=2 in Figure 1, S1, S2). For biological replicates, an n of at least 5 is generally expected in hematopoietic studies to account for individual mouse variability. The authors should justify the small n or supplement these datasets.

Answer: We agree with the reviewer that a higher number of experimental animals increases the statistical robustness of the results. For each experimental condition, we performed at least three independent experiments. The figures show representative data from one of these experiments, each involving varying numbers of littermates, with consistent outcomes across replicates. As we repeatedly obtained comparable results, we conclude that the observed effects are reliable. We have now included additional statistical information in the figure legends.

Estimated timeframe: The additional in vivo and mechanistic experiments requested above would likely require several months (approximately 3-6 months) to complete.

Reviewer #3 (Comments to the Authors (Required)):

Septin7 is essential in early hematopoiesis, but redundant at later stages
Natalia Ronkina, Nikita A. Verheyden, Prerna Gambhir, Kathrin Laaß, Tatiana Yakovleva, Anneke Dörrie, Megha Abbey, Manoj B. Menon, Anton Selich, Melanie Galla, Michael Rothe, Axel Schambach, Andreas Krueger, Alexey Kotlyarov, Matthias Gaestel

The manuscript investigates the consequences of Sept7 loss on early haematopoietic progenitors and downstream lymphoid populations.
I consider it important to report negative and unexpected data, as these findings add transparency and context to a study's conclusions. However, many phenotypes associated with Sept7 disruption have been reported previously. To strengthen the rationale and overall impact, the study would benefit from a clearer articulation of why further interrogation of Sept7 was necessary, and-crucially-what novel insights this work provides beyond existing knowledge. Highlighting these specific contributions would help clarify the unique value of the present study.

We thank Reviewer 3 for the positive evaluation of our manuscript and for the constructive comments, which have helped us to improve the quality of the manuscript.

The authors should clarify whether the samples presented in Figure 1c (splenocytes, thymocytes, and bone marrow) were collected from the same individual mice.

Answer: We thank the reviewer for this advice. Splenocytes, thymocytes and bone marrow cells were isolated from Vav1-iCre::Sept7wt/flox and Vav1-iCre::Sept7flox/flox littermates. The same samples were divided into three portions and used for PCR genotyping, Western blot analysis, and flow cytometry (FACS), respectively. Two mice per genotype were initially used. This information has now been included in the corresponding figure legends. During the revision process, we performed additional experiments including further mice. We have modified the Figure 1c and expanded the figure legends to include these additional statistical details. We have also included all statistical data in the supplementary figures.

The manuscript states that thymocytes show normal T cell development. Are these thymocytes expressing SEPT7? How do the authors confirm that the thymic cells analysed are derived from Sept7-null progenitors rather than WT cells?

Answer: We thank the reviewer for highlighting this important point.. Given that Sept7 is virtually completely deleted at the DN3b stage of T-cell development in hCD2-iCre::Sept7flox/flox mice (Fig. 2d and S4B) and no substantial frequencies of escapees emerged in this experimental model, we conclude that SEPT7 is not essential for proliferation during T-cell development at or beyond the DN3b stage.

The western blot in Figure 1b appears cropped. The authors should explain the rationale for cropping, provide full blots in supplementary material, and clearly indicate all included lanes.

Answer: We have now included all full blots in the Supplementary Information and indicated the lanes shown in the figures.

Has Sept7 deletion efficiency been quantified for each mouse shown in Figure 1d? If so, these data should be presented or referenced.

Answer: The efficiency of Sept7 deletion was not quantified in the mice used for Fig. 1d and 2c. These mice were genotyped by tail biopsy and used for thymocyte analyses, including surface expression of CD4, CD8, TCRβ, CD69, and CD25, as well as intracellular expression of TCRβ and SEPT7 (Supplementary Fig. S3 and S4).

Figures throughout the manuscript require more coherent annotation. When multiple mice are shown, the corresponding rows or plots should be labelled with mouse identifiers. Axis labels should be clearer, and the authors should define terms such as "normalised to mode" and "max %". For transparency, actual values (e.g., percentages, cell numbers) should be provided.

Answer: We thank the reviewer for this important suggestion. We have unified the color coding across figures and included the corresponding statistical data in the Supplementary Information. We have also clarified the term 'max %' in the Materials and Methods section ('Flow cytometry analysis') as follows: "Data are presented as total counts (count) or as percentage of maximum (% Max), where the highest peak (mode) of each sample is set to 100%, allowing easier visual comparison of distribution shapes between samples with differing total cell counts.'"

Figures 1e and 2d do not appear to add value to the central narrative and can be removed without loss of clarity.

Answer: We agree with the reviewer that Figures 1e and 2d do not add substantial value to the central narrative. Nevertheless, we prefer to retain these figures as they may help readers better understand the main experimental observations.

The two cell populations within Figure 5e should be displayed using distinguishable colors rather than grey and white, which are difficult to differentiate. Even if the conclusion is that the populations are similar, this should be visually apparent from the figure.

Answer: We thank the reviewer for this helpful suggestion. We have revised the color scheme to better distinguish between the two cell populations.

Axis labels (Figure 5e) should include the fluorophore names rather than only wavelengths to improve interpretability.

Answer: We thank the reviewer for this insight and have included the name of the cell tracker in Figure 5e.

In the Methods section (lines 326, 330, and 333), "Floxt-3L" should be corrected to "Flt-3L".

Answer: We thank the reviewer for this helpful comment and have now corrected the error.

The Hoxb8-Fl system typically supports multilineage potential. Have the authors characterised the Hoxb8-Septflox/flox cells to confirm whether they retain multilineage output, or have they inadvertently selected for cells with lymphoid bias?

Answer: We thank the reviewer for this important and helpful correction. We have revised the description of Hoxb8-HSPCs accordingly: "Previous studies have shown that Hoxb8-immortalized HSPCs lose self-renewal capacity and megakaryocyte/erythroid differentiation potential while retaining myeloid and lymphoid potential (Redecke et al., 2013)." This statement has now been incorporated into the main text of the manuscript in the section "Generation of Hoxb8-immortalized Sept7 KO HSPCs.".

The manuscript would benefit from overall simplification and clarification. Several sections are unnecessarily complex and obscure the main findings.

Answer: We have revised the Introduction and Discussion to simplify and clarify the main finding.

Several claims are speculative and not supported by the presented data. Examples include:

"Incomplete deletion of SEPT7 from hematopoietic cells might indicate impaired repopulation activity... and selective advantages of SEPT7 expressing cells."

No evidence is provided for repopulation defects or selective advantage. Alternative explanations (e.g., cell death or failure to proliferate) are equally plausible.

Answer: This is a critical point that we addressed in our previous study (Kandi et al., 2021). Competitive repopulation experiments using Vav1-iCre::Sept7flox/flox mice demonstrated that hematopoietic stem cells (HSCs) derived from these mice fail to engraft and establish donor chimerism after transplantation, indicating that SEPT7 is essential for HSC function under stress conditions. In the present study, we provide evidence that indirectly supports an essential role for SEPT7 in HSCs, while showing that SEPT7 is dispensable for in vivo lymphoid development and ex vivo proliferation of Hoxb8-immortalized progenitors. The inefficient Vav1-iCre–mediated recombination of Sept7flox/flox, which could not be overcome by additional Cre expression, suggests that HSCs may evade complete Sept7 deletion. Accordingly, the reduced engraftment capacity of HSCs from Vav1-iCre::Sept7flox/flox mice (Kandi et al., 2021) likely reflects exhaustion of repopulating potential due to selection against Sept7-deficient cells. We have now clarified and more precisely discussed these observations in the Discussion section.

"These observations suggest a requirement of SEPT7 for early stages of hematopoiesis and redundancy at later stages..."

The authors examine primarily lymphoid cells, which can differentiate without extensive cell cycling. The statement should be made population specific rather than applying to "later stages of hematopoiesis" broadly.

Answer: In our current study we focused our analysis on intrathymic T-cell development. We hypothesized that periodic strong proliferative bursts followed by cell-cycle inactivity during somatic recombination of antigen-receptor genes might reveal proliferation-driven selection pressure. Overall development of T cells from Vav1-iCre::Sept7flox/flox and hCD2-iCre::Sept7flox/flox mice was unimpaired. Given that DN3b and DN4 are heavily proliferating under normal circumstances we concluded that Sept7 is dispensable for their proliferations. Otherwise T-cell development would result in strong competition in favor of Sept7-positive cells, which we do not observe: SEPT7-negative cells are maintained even through stages of high proliferative activity (DN3b and DN4) (Figure 2c and new Figure S4B). We also did not observe any differences in T- or B-cell development between K-Ras-G12D::hCD2-iCre::Sept7flox/flox and K-Ras-G12D::hCD2-iCre::Sept7wt/flox mice, neither in the thymus nor in the spleen (new Figures S5C and S6C). This observation led us to conclude that SEPT7 is not essential for lymphoid cell development, even under conditions of

physiological stress. Furthermore, ex vivo proliferation of Hoxb8-immortalized HSPCs—which lose self-renewal capacity and megakaryocyte/erythroid differentiation potential while retaining myeloid and lymphoid potential (Redecke et al., 2013)—supports the conclusion that SEPT7 is dispensable not only for lymphoid cells, but also for myeloid cells at later stages of hematopoiesis, when self-renewal capacity is no longer required. We have included new data in the revised version of our manuscript that further strengthen our conclusion regarding the requirement of SEPT7 during early hematopoiesis. Specifically, we show that SEPT7-expressing escapees arise as early as embryonic day 14.5, and that their proportion among lineage-negative hematopoietic progenitors increases during ontogenesis (new Figures 6F, 6G, S9 and S10), supporting the existence of selective pressure against SEPT7 deficiency, potentially due to impaired long-term maintenance of SEPT7-deficient progenitors. We have incorporated this discussion into the main text in the section entitled "Generation of Hoxb8-immortalized Sept7 KO HSPCs."

"The distinct kinetics of HSC self-renewal across life stages may explain the essential role of SEPT7 in early but not later hematopoiesis."
This statement is not connected to the preceding discussion and appears inconsistent with the data. The authors do not examine fetal hematopoiesis, nor do they detect Sept7 deletion in adult bone marrow cells (Figure 1), making this interpretation unsupported.

Answer: We thank the reviewer for the constructive criticism. To further support our conclusions, we performed additional experiments: "To test our hypothesis that SEPT7 is required during early hematopoiesis, we examined its expression in nucleated hematopoietic (CD45-positive) fetal liver cells. Vav1-iCre is strongly expressed and active in the fetal liver from embryonic day 9.5 (E9.5) to approximately E11.5 and later, enabling efficient Cre-mediated recombination in hematopoietic stem cells and all downstream hematopoietic lineages (He et al, 2008). At E14.5, we observed a marked reduction, although not a complete loss, of SEPT7 expression in CD45-positive fetal liver cells (new Figure 6F). Notably, approximately 20% of hematopoietic (CD45-positive) fetal liver cells remained SEPT7-positive at this stage, suggesting incomplete SEPT7 deletion in a subset of hematopoietic cells during embryonic development (new Figure S9). Interestingly, in the bone marrow of adult mice, approximately 46% of lineage-negative HSPCs remained SEPT7-positive, indicating a substantial increase in the proportion of SEPT7-expressing escapees among hematopoietic progenitors during ontogenesis (new Figures 6G and S10). Notably, the proportion of SEPT7-positive escapees further increased to 86.5% in the overall bone marrow cell population (modified Figure 1C and new Figure S1C), suggesting progressive enrichment of SEPT7-positive cells during hematopoiesis. This observation supports the existence of selective pressure against SEPT7 deficiency, potentially due to impaired long-term maintenance of SEPT7-deficient progenitors." We have incorporated this data into the main text in the section entitled "Generation of Hoxb8-immortalized Sept7 KO HSPCs."

"At later stages... the role of SEPT7 in cell division becomes redundant."
No evidence is provided to support redundancy of SEPT7 in proliferating hematopoietic populations. Hematopoietic cells continue to divide to maintain homeostasis. The authors should clarify that they are referring to specific cell types rather than the hematopoietic system as a whole.

Answer: Our results demonstrate that Cre-induced deletion of the floxed Septin7 allele is inefficient during the early stages of hematopoiesis, suggesting strong selection pressure against Septin7 deficiency at this stage. Moreover, we observe a substantial increase in the proportion of SEPT7-expressing escapees among hematopoietic progenitors during ontogenesis (see our response to the previous question). In contrast, Septin7 deletion at later stages of hematopoiesis—specifically at the common lymphoid progenitor stage—as well as in Hoxb8-immortalized myeloid and lymphoid progenitors, was highly efficient and does not lead to detectable defects in cell division or to an increased frequency of SEPT7-expressing escapees. Furthermore, we show that Septin7 is dispensable for lymphoid development even under conditions of physiological stress induced by oncogenic K-Ras–driven tumorigenesis. Taken together, these findings indicate that Septin7 is essential during early hematopoiesis but becomes dispensable at later stages of hematopoietic development.

We would like to thank the reviewers again for taking the time to review our manuscript.

June 22, 2026

RE: Life Science Alliance Manuscript #LSA-2026-03637-TR

Dr. Natalia Ronkina
MHH
Biochemistry
Carl-Neuberg-Straße 1
Hannover, Lower Saxony 30625
Germany

Dear Dr. Ronkina,

Thank you for submitting your revised manuscript entitled "Septin7 is essential in early hematopoiesis, but redundant at later stages". Your revised manuscript was assessed by all the original reviewers, whose comments are included below. As you will read, the reviewers note that most of their previous concerns have been addressed in the revised version and have also positively commented on the improved revised version. Reviewers 2 and 3 have indicated some pending minor concerns.

In line with the reviewers' evaluation, we would be happy to publish your paper in Life Science Alliance pending resolution of the reviewers' minor points in your ms text and final revisions necessary to meet our formatting guidelines.

MANUSCRIPT ORGANIZATION AND FORMATTING:

To avoid unnecessary delays in the acceptance and publication of your paper, please read the following information carefully. Full guidelines are available on our Instructions for Authors page, https://www.life-science-alliance.org/authors

Submission of a paper that does not conform to Life Science Alliance guidelines will delay the acceptance of your manuscript.
-We encourage you to make the scale bars more prominent (Figures 4, 5, 6 and S7). Please include scale bar size and information for S8B.
-Please include size markers for gels shown in Fig 1A, 2A, 3A, E, 4C, 5A, and 6A
-Kindly provide a source or citation for Sep 7 transcript variant 2 (NM_001205367.1) cDNA.
-Please provide concentration of primary antibodies used in experiments.
-Please expand on imaging details to include information on objectives used and excitation/emission filters.
-You have the option of using Figure 7 as a Graphical Abstract in which case you would have to remove this figure file and upload it instead as a graphical abstract file, and edit the text accordingly.
-Please add the X and Bluesky handles of your host institute/organization, as well as your own, and/or one of the authors, in our system.
-Please remove track changes from the manuscript and upload a clean version. This version that highlights the changes, please upload as a related manuscript file.
-Please rename "Competing interest statement" to "Conflict of Interest".
-Please move your main and supplementary figure legends to after the references section.
-Please add callouts for Figures S1A-C; S6B; S7A-C and S9A-B to your main manuscript text.
-The "Data Availability" section should be placed after the Materials & Methods section. Please consult our guidelines at https://www.life-science-alliance.org/manuscript-prep#format.
-Please be sure that the authorship listing and order is correct

We encourage our authors to provide original source data, particularly uncropped/-processed electrophoretic blots and spreadsheets for the main figures of the manuscript. If you would like to add source data, we would welcome one PDF/Excel-file per figure for this information. These files will be linked as supplementary "Source Data" files.

It is Life Science Alliance policy that if requested, original data images must be made available to the editors. Failure to provide original images upon request will result in unavoidable delays in publication. Please ensure that you have access to all original data images prior to final submission.

If you are planning a press release on your work, please inform us immediately to allow informing our production team and scheduling a release date.

We welcome submissions of potential cover images for the issue of LSA in which your work would appear. If you have high quality images associated with this work, please feel free to email these, with a caption, to the journal office.

LSA encourages authors to provide a 30-60 second video where the study is briefly explained. These videos will be appear embedded with the manuscript online at Life Science Alliance, and on social media to promote the published paper and authors (for examples, see https://docs.google.com/document/d/1-UWCfbE4pGcDdcgzcmiuJl2XMBJnxKYeqRvLLrLSo8s/edit?usp=sharing). Corresponding or first-authors are welcome to submit the video. Please submit only one video per manuscript. The video can be emailed to contact@life-science-alliance.org

FINAL FILES:

To upload the final version of your manuscript, please log in to your account: https://lsa.msubmit.net/cgi-bin/main.plex
You will be guided to complete the submission of your revised manuscript and to fill in all necessary information. Please get in touch in case you do not know or remember your login name.

The following items are required for acceptance.

-- An editable version of the final text (.DOC or .DOCX) is needed for copyediting (no PDFs).

-- High-resolution figure, supplementary figure and video files uploaded as individual files: See our detailed guidelines for preparing your production-ready images, https://www.life-science-alliance.org/authors

-- Summary blurb (enter in submission system): A short text summarizing in a single sentence the study (max. 200 characters including spaces). This text is used in conjunction with the titles of papers, hence should be informative and complementary to the title. It should describe the context and significance of the findings for a general readership; it should be written in the present tense and refer to the work in the third person. Author names should not be mentioned.

The license to publish form must be signed before your manuscript can be sent to production. A link to the license to publish form will be available to the corresponding author only. Please take a moment to check your funder requirements.

Reviews, decision letters, and point-by-point responses associated with peer-review at Life Science Alliance will be published, alongside the manuscript. If you do want to opt out of having the reviewer reports and your point-by-point responses displayed, please let us know immediately.

Thank you for your attention to these final processing requirements. Please revise and format the manuscript and upload materials as soon as you are able.

Thank you for this interesting contribution to the literature. We look forward to publishing your paper in Life Science Alliance.

Sincerely,

Sarita Hebbar, PhD
Scientific Editor
Life Science Alliance
http://www.lsajournal.org

Reviewer #1 (Comments to the Authors (Required)):

This manuscript is a revised version of an earlier submission by Ronkina N et al. This study examines the role of Septin 7 in hematopoiesis. Septin7 has been shown to play crucial role in cytokinesis in many cell types with Septin7-null mice showing embryo lethality. However, in hematopoietic, this study shows that it does not play any significant role in cytokinesis. The authors had built on their previously published reports and show the lineage-specific roles of the protein. Major concerns raised by me were linked with incomplete deletion of the gene and the resulting paradoxical observations. The authors have added explanations and have additional data addressing other concerns that I had raised. The results on the fetal liver derived HSCs are specially valuable. I have gone through the changes and additions incorporated in the manuscript and am satisfied with the revisions brought about in the manuscript.

Reviewer #2 (Comments to the Authors (Required)):

This manuscript examines the role of SEPT7 during hematopoiesis using several conditional knockout models and Hoxb8-immortalized progenitors. The authors show that SEPT7 appears to be essential during early hematopoietic development and HSPC function, while later lymphoid development and proliferation of Hoxb8 progenitors can occur efficiently in its absence. Overall, the revised manuscript is significantly improved and the additional experiments address most of the major concerns raised during the initial review.

In particular, the added K-Ras stress model, fetal liver analyses, and ex vivo T-cell proliferation experiments strengthen the conclusion that SEPT7 requirement is context- and stage-dependent. The manuscript also provides some observations regarding the emergence and enrichment of SEPT7-positive escapee populations during hematopoietic development. Although the mechanistic basis for the differential requirement of SEPT7 between early HSPCs and later progenitors remains incompletely understood, I do not think this substantially weakens the main conclusions of the paper. Further mechanistic work would certainly be interesting, but likely goes beyond the scope of the current study.
I only have a few minor suggestions for the revised manuscript:
1. Some conclusions should be phrased more carefully. In particular, statements suggesting that SEPT7 is dispensable for cytokinesis may be too strong based on the current data. The authors directly demonstrate continued proliferation of Hoxb8-immortalized progenitors in the absence of SEPT7, but not necessarily completely normal cytokinesis.
2. The number of biological replicates and statistical tests should be clearly indicated throughout all figure legends.

Reviewer #3 (Comments to the Authors (Required)):

The manuscript investigates the consequences of Sept7 loss on early haematopoietic progenitors and downstream lymphoid populations.

The authors have addressed all my comments from the first round of review. Upon the following very minor revisions I believe the manuscript is suitable for acceptance.

Line 53 - spelling mistake "Sept7fox/flox"
Line 100 - additional full stop after deletion
Line 637 - figure legend for Figure S2 should be separated onto it's own line.
Line 633 - spelling mistake "peroferal"
Axis of all facs plots should be labelled with corresponding antibody and fluorophore.
Line 644 - remove additional bracket after CD8-
Line 663 - change compare to "in comparison"
Line 676 - remove additional bracket after CD8-

July 1, 2026

RE: Life Science Alliance Manuscript #LSA-2026-03637-TRR

Dr. Natalia Ronkina
MHH
Biochemistry
Carl-Neuberg-Straße 1
Hannover, Lower Saxony 30625
Germany

Dear Dr. Ronkina,

Thank you for addressing our formatting requests and submitting your Research Article entitled "Septin7 is essential in early hematopoiesis, but redundant at later stages". It is a pleasure to let you know that your manuscript is now accepted for publication in Life Science Alliance. Congratulations on this interesting work.

The final published version of your manuscript will be deposited by us to PubMed Central upon publication.

Your manuscript will now progress through copyediting and proofing. It is journal policy that authors provide original data upon request.

Your article will publish open access upon publication under a CC-BY license.

Reviews, decision letters, and point-by-point responses associated with peer-review at Life Science Alliance will be published, alongside the manuscript. If you do want to opt out of having the reviewer reports and your point-by-point responses displayed, please let us know immediately.

IMPORTANT: If you will be unreachable at any time, please provide us with the email address of an alternate author. Failure to respond to routine queries may lead to unavoidable delays in publication.

Scheduling details will be available from our production department. You will receive proofs shortly before the publication date. Only essential corrections can be made at the proof stage so if there are any minor final changes you wish to make to the manuscript, please let the journal office know now.

DISTRIBUTION OF MATERIALS:
Authors are required to distribute freely any materials used in experiments published in Life Science Alliance. Authors are encouraged to deposit materials used in their studies to the appropriate repositories for distribution to researchers.

You can contact the journal office with any questions, contact@life-science-alliance.org

Again, congratulations on a very nice paper. I hope you found the review process to be constructive and are pleased with how the manuscript was handled editorially. We look forward to future exciting submissions from your lab.

Sincerely,

Sarita Hebbar, PhD
Scientific Editor
Life Science Alliance
http://www.lsajournal.org
